# Comparative RNA-Sequencing Analysis Reveals High Complexity and Heterogeneity of Transcriptomic and Immune Profiles in Hepatocellular Carcinoma Tumors of Viral (HBV, HCV) and Non-Viral Etiology

**DOI:** 10.3390/medicina58121803

**Published:** 2022-12-07

**Authors:** Liliana Paslaru, Gabriela Bindea, Anca Nastase, Andrei Sorop, Cristian Zimbru, Vlad Herlea, Doina Hrehoret, Vlad Brasoveanu, Radu Zamfir, Simona Dima, Irinel Popescu

**Affiliations:** 1 Fundeni Clinical Institute, 022328 Bucharest, Romania; 2 Institut National de la Santé et de la Recherche Médicale (INSERM), Laboratory of Integrative Cancer Immunology, 75006 Paris, France; 3 Centre de Recherche des Cordeliers, Sorbonne Université, Université de Paris, 75006 Paris, France; 4 Equipe Labellisée Ligue Contre le Cancer, 75006 Paris, France; 5 Department of Automation and Applied Informatics, Politehnica University Timisoara, 300223 Timisoara, Romania

**Keywords:** hepatocellular carcinoma, HBV, HCV, RNA-seq, immune infiltrate, biomarkers

## Abstract

*Background and Objectives*: Hepatocellular carcinoma (HCC), the most common type of primary liver cancer, is the leading cause of cancer-related mortality. It arises and progresses against fibrotic or cirrhotic backgrounds mainly due to infection with hepatitis viruses B (HBV) or C (HCV) or non-viral causes that lead to chronic inflammation and genomic changes. A better understanding of molecular and immune mechanisms in HCC subtypes is needed. *Materials and Methods*: To identify transcriptional changes in primary HCC tumors with or without hepatitis viral etiology, we analyzed the transcriptomes of 24 patients by next-generation sequencing. *Results*: We identified common and unique differentially expressed genes for each etiological tumor group and analyzed the expression of SLC, ATP binding cassette, cytochrome 450, cancer testis, and heat shock protein genes. Metascape functional enrichment analysis showed mainly upregulated cell-cycle pathways in HBV and HCV and upregulated cell response to stress in non-viral infection. GeneWalk analysis identified regulator, hub, and moonlighting genes and highlighted *CCNB1*, *ACTN2*, *BRCA1*, *IGF1, CDK1*, *AURKA*, *AURKB*, and *TOP2A* in the HCV group and *HSF1*, *HSPA1A*, *HSP90AA1*, *HSPB1*, *HSPA5*, *PTK2*, and *AURKB* in the group without viral infection as hub genes. Immune infiltrate analysis showed that T cell, cytotoxic, and natural killer cell markers were significantly more highly expressed in HCV than in non-viral tumors. Genes associated with monocyte activation had the highest expression levels in HBV, while high expression of genes involved in primary adaptive immune response and complement receptor activity characterized tumors without viral infection. *Conclusions*: Our comprehensive study underlines the high degree of complexity of immune profiles in the analyzed groups, which adds to the heterogeneous HCC genomic landscape. The biomarkers identified in each HCC group might serve as therapeutic targets.

## 1. Introduction

Liver cancer is the sixth most commonly diagnosed cancer and the third most common cause of cancer deaths worldwide [1] (GLOBOCAN 2020 report (https://gco.iarc.fr/today (accessed in May 2021)); Table 1).

Hepatocellular carcinoma (HCC) is the major type of primary liver cancer. It is a very aggressive and challenging cancer with a dismal prognosis. In recent decades, incidence rates have increased in different countries [2,3]. The sequential use of sorafenib (front line), regorafenib, and, most recently, ramucirumab (second line) provides a survival benefit in advanced HCC [4,5], but actually these drugs only prolong survival in the range of months, while prognosis for advanced stages remains poor. Consequently, new targets for therapeutic development are needed. The lack of a more robust response to systemic therapies may be due to the heterogeneous nature of HCC, which is related to numerous etiological factors [6,7,8], such as hepatitis B and C viruses (HBV, HCV), autoimmune hepatitis, chronic alcohol abuse, aflatoxins, hemochromatosis, fatty liver disease, androgenic steroid use, obesity, diabetes mellitus, etc. [9].

HCC might originate in mature liver cells or in progenitor cells. Hence, the molecular basis of HCC progression may differ depending on diverse factors and, therefore, a number of mechanisms might be involved [10].

The majority of cases develop underlying cirrhosis, while a smaller number of patients do not develop cirrhosis [11].

The mechanisms by which these multifactorial etiologies lead to cirrhosis and HCC are not well understood.

Hepatic carcinogenesis is likely due to both direct effects of underlying liver insult and indirectly to hepatocyte inflammation and regeneration.

HBV is considered a carcinogenic virus, which induces chronic necroinflammatory disease.

Viral HBV DNA is commonly integrated into the genome, promoting mutations in liver cells and leading to HCC [12]. HBV increases the risk of HCC even in the absence of cirrhosis [13].

Chronic HCV infection is another well-established factor that increases the risk of HCC (by 10–20-fold).

HCV is an RNA virus that does not integrate into the host’s genome and is not a primary initiator of tumorigenesis. More likely, HCV promotes tumorigenesis as a consequence of associated cirrhosis, producing repetitive damage, regeneration, and fibrosis. Patients with advanced fibrosis or cirrhosis are at increased risk for carcinogenesis because chromosomal alterations that occur in fibrotic tissue are associated with tumor formation [14,15,16].

Hepatitis B virus (HBV) and hepatitis C virus (HCV) contribute to HCC directly by modulating pathways that promote the malignant transformation of hepatocytes and indirectly by promoting long-term liver damage, chronic inflammation, cell death, regeneration, and oxidative DNA damage [17,18].

Different studies have revealed that nearly every carcinogenic pathway is altered to some degree in HCC [19,20].

The high heterogeneity of HCC tumors, characterized by genomic instability, microenvironmental changes, and molecular and pathway aberrations, is a major contributor to the high lethality rate. It has an impact on patients’ poor prognoses and lack of response to standard therapies. The heterogeneity also complicates patient stratification and response prediction.

Owing to the frequency of late-stage diagnosis, and in spite of the multiple treatment methods available, such as systemic therapy, liver resection, percutaneous ethanol injection, microwave ablation, arterial chemoembolization, and liver transplantation, the prognosis for HCC remains poor (a reported 5-year survival rate of only 7%) [21].

In order to optimize the outcome of patients, it is essential to study and establish the most common etiological factors that generate tumoral heterogeneity [22].

To define those patients who may truly benefit from systemic therapy, HCC clinical trials should include a definite stratification of patients according to one of the clinical prognostic scoring systems (e.g., Child–Pugh, etc.) and stratification by disease etiology (for example, HBV-related, HCV-related, or other) [20].

The aim of our comparative study was to monitor transcriptome changes by whole transcriptome sequencing and to analyze the gene expression profiles in three groups of patients and show the role of viral (HBV, HCV) or non-viral etiologies in HCC tumoral heterogeneity.

## 2. Materials and Methods

### 2.1. Patient Selection and Sample Collection

Twenty-four patients with primary HCC who underwent a curative liver resection in the General Surgery Department at the Fundeni Clinical Institute, Bucharest, Romania, were selected for whole-transcriptome sequencing analysis.

The patients were divided into 3 groups: 8 with HBV, 8 with HCV, and 8 without viral infection. The patients’ demographic and clinical features are listed in Supplementary Table S1, and representative histology (hematoxylin–eosin staining) images are presented in Supplementary Figure S1. The mean age was 57 years for the HBV group, 64 years for the HCV group, and 62 years for the non-viral group, thus indicating no significant differences between the average ages for these HCC groups (*p* = 0.4251). The differences between serum AFP levels were statistically significant for all 3 HCC groups (*p* = 0.0451), and PIVKA (Protein Induced by Vitamin K Absence-II) markers showed increased levels in the non-viral group compared with the HBV group.

Forty-eight liver tissues samples consisting of tumor and adjacent non-tumor samples (8 pairs from HBV-positive patients, 8 pairs from HCV-positive patients, and 8 pairs from viral-negatives patients) were collected at the time of surgery in RNA later stabilizing solution (Sigma, St. Louis, MO, USA) and stored at − 80 °C until the collection of all samples.

The study conformed to the ethical guidelines of the 1975 Declaration of Helsinki and was approved by the Ethics Committee of the Fundeni Clinical Institute (29435/21.07.2016). All patients signed a written informed consent form. Follow-up was completed in March 2021. The period of follow-up was defined from the date of surgery to the date of the patient’s death or the last follow-up point.

### 2.2. RNA Isolation

Total RNA from HCC and paired non-tumoral tissue samples was isolated using TRIzol reagent, according to the manufacturer’s instructions (Invitrogen, Carlsbad, CA, USA). Isolated RNA was eluted using RNase-free water and stored at −80 °C.

### 2.3. RNA Quantification and Quality Assessment of Isolated RNA

RNA purity and concentration were measured with a NanoDrop™ ND-1000 spectrophotometer (Thermo Fisher Scientific, Waltham, MA, USA). For further assessment of RNA quality and relative size, the Eukaryote total RNA assay kit and the Agilent 2100 Bioanalyzer (Agilent Technologies, Santa Clara, CA, USA) were used to calculate RIN (RNA integrity number) values. In our study, an RNA integrity number (RIN) value > 9 indicated a high-quality sample satisfactory for downstream sequencing.

### 2.4. Total RNA Library Preparation and Sequencing

RNA-sequencing (RNA-seq) libraries were generated using an Illumina TruSeq Stranded Total RNA LT sample preparation kit (with RiboZero Gold) (Illumina, part nos. RS-122–2301 and RS-122–2302), following the manufacturer’s protocol specifications (part no. 15031048 Rev. E October 2013). Ribosomal depletion was performed with 1 µg of total RNA using Ribo-Zero Gold before a heat-fragmentation step that targeted generating libraries with insert sizes ranging from 120 to 200 bp; thus, the remaining RNA was purified, fragmented, and primed for cDNA synthesis. The purified and fragmented RNA was then used to generate cDNA using SuperScript II Reverse Transcriptase (Invitrogen, catalog no. 18064) and random primers. The synthesized cDNA was then transformed into double-stranded DNA incorporating dUTP in place of dTTP to prevent subsequent amplification of the second strand and therefore improve the library’s strand specificity. Libraries were subjected to 15 cycles of PCR after 3′ adenylation and adaptor ligation steps to generate selectively enriched RNA-Seq libraries suitable for sequencing.

The RNA-seq libraries were evaluated prior to sequencing using an Agilent BioAnalyzer 2100 System and an Agilent DNA 1000 kit (Agilent, part no. 5067-1504) to determine the quality and distribution of DNA fragments. Next, the final library concentration was determined using the Qubit dsDNA HS assay (Thermo Fisher Scientific).

The RNA-seq libraries were standardized to 10 nM, denatured with 0.2 N NaOH, then diluted to 20 pM for downstream sequencing. Sequencing of denatured libraries was carried out in accordance with the manufacturer’s standard (Illumina, document no. 15048776 v04, May 2018) using a NextSeq500 platform and NextSeq 500 High Output Kit v2 (150 cycles; up to 400M reads) kits (Illumina, San Diego, CA, USA, catalog no. FC-404-2002).

### 2.5. Analysis of Sequencing Data—Identification of Differentially Expressed Genes (DEGs)

The analysis of sequencing data was performed in collaboration with Illumina by quantifying gene expression against the human genome (version GRCh37 (hg19)) in the Illumina BaseSpace Platform workflow (version 2.1.0), according to the Tuxedo pipeline (Supplementary Figure S2), which included the following versions of open-source software: RNA-Seq Alignment (BaseSpace Workflow v2.1.0), Isis (Analysis Software v2.6.25.18), TopHat (Aligner v2.1.0), Isaac (Variant Caller v2.3.13-31-g3c98c29-dirty), IONA (Annotation Service v1.0.10.37), Bowtie2 (Aligner v 2.2.6), BEDTools (v2.17.0), Cufflinks (v2.2.1), and BLAST (v2.2.26+).

The CummeRbund program was used for initial data exploration, analysis, and visualization [23].

### 2.6. Functional Enrichment Analysis

Functional enrichment analysis was performed using METASCAPE [24]. For each gene list, pathway and process enrichment analyses were carried out using: the KEGG Pathway, GO Biological Processes, Reactome Gene Sets, Canonical Pathways, Cell Type Signatures, CORUM, TRRUST, DisGeNET, PaGenBase, Transcription Factor Targets, WikiPathways, PANTHER Pathway, and COVID. All genes in the genome were used as the enrichment background. Terms with a *p*-value < 0.01, a minimum count of 3, and an enrichment factor >1.5 (the ratio between the observed counts and the counts expected by chance) were collected and grouped into clusters based on their membership similarities. More specifically, *p*-values were calculated based on cumulative hypergeometric distributions, and *q*-values were calculated using the Benjamini–Hochberg procedure to account for multiple testing. Kappa scores were used as the similarity metrics when performing hierarchical clustering of the enriched terms, and sub-trees with a similarity of >0.3 were considered as clusters. The most statistically significant term within a cluster was chosen to represent the cluster [24]. Relevant gene functional analysis was performed using GeneWalk, PMID: 33526072 [25]. Genes of interest (hub genes or moonlighting genes) were selected using the following thresholds: global_padj < 0.1; ncon_gene ≥ 50; and ncon_go ≥ 50 [25].

### 2.7. Protein–Protein Interaction (PPI) Network Analysis of DEGs

Interactions of 3 group of DEGs were shown in the STRING online database (http://string-db.org (accessed on 5/6 December 2021)) [26]. Network nodes represented proteins and edges represented protein–protein associations.

### 2.8. Immune Infiltrate Analysis

The immune infiltrates in HCC samples were investigated using Immunome [27]—a compendium of immune cell markers preferentially expressed in the majority of immune subtypes infiltrating tumors.

The raw count data for tumors and normal samples were analyzed. The quality control revealed that there was no batch effect on the samples. Genes with fewer than 10 counts were removed. Matrices with raw counts were log2-normalized using the TMM method “edgeR”. Differential expression analysis was performed with Limma-Voom. Enrichment analyses were performed with Cytoscape Apps [28], ClueGO [29], and CluePedia [30].

The Cancer Genome Atlas (TCGA) hepatocellular carcinoma cohort was downloaded as HTseq raw counts using R (biomaRt) [31].

### 2.9. Validation of Target mRNA Levels Using Quantitative Real-Time PCR

We carried out validation by quantitative reverse transcriptase (RT) real-time PCR for a selected group of genes (*BIRC5* and *SLC22A1* in the HCV group; *CLEC1B* in the HBV group; *FGFR4*, *HSF1*, *RNF187*, *HSP90AB1*, and *HSPB1* in the group without viral infection; and *HGF*, *COLEC10*, and *CYP17A* as genes common to all groups) to validate our next-generation sequencing (NGS) results. These genes were selected based on their up- or downregulation in our HCC samples.

A High-Capacity cDNA Reverse Transcription Kit (Applied Biosystems, Thermo Fisher Scientific, Waltham, MA, USA) was used to synthesize 2000 ng cDNA by incubation as follows: 25 °C for 10 min, 37 °C for 120 min, 85 °C for 5 min, and 4 °C for 5 min. The amplification steps were performed using SYBR Green PCR Master Mix (Applied Biosystems, Thermo Fisher Scientific) with the following thermocycler protocol: 95 °C for 10 min + (95 °C for 15 s; 60 °C for 1 min) for 40 cycles. The ABI PRISM 7300 Detection System (Applied Biosystems, Thermo Fisher Scientific) was used to analyze the relative expression all target genes normalized to b-actin. The expression levels of all target genes were related as fold changes 2^−ΔΔCt^. The primers were designed and synthesized by Kaneka Eurogentec S.A. Liège (Supplementary Table S2).

### 2.10. Gene Expression Validation

We verified our gene expression results by qPCR by comparing them with curated databases, such as HCCDB [32], UALCAN [33], CTdatabase [34], and TCGA, and data curated from published research articles.

## 3. Results

### 3.1. Differentially Expressed Gene (DEG) Analysis

Although initially the number of differentially expressed genes (DEGs) was very high after setting the filtering threshold of the *q*-value to 0.05, the number of DEGs significantly dropped.

A first observation was that in each group the gene expression in tumoral tissues was significantly different from that in non-tumoral tissues.

In addition, the total number of DEGs varied between the three groups.

Table 2 shows the number of up- and downregulated genes in the HBV, HCV, and non-viral (non-B, non-C) groups.

The highest numbers of upregulated and downregulated genes were identified in the Total HCV group (with 465 upregulated and 226 downregulated) and the lowest number of DEGs was identified in the HBV group (120 upregulated and 102 downregulated).

### 3.2. Identification of “Common” and “Unique” Genes

**Figure 1:**
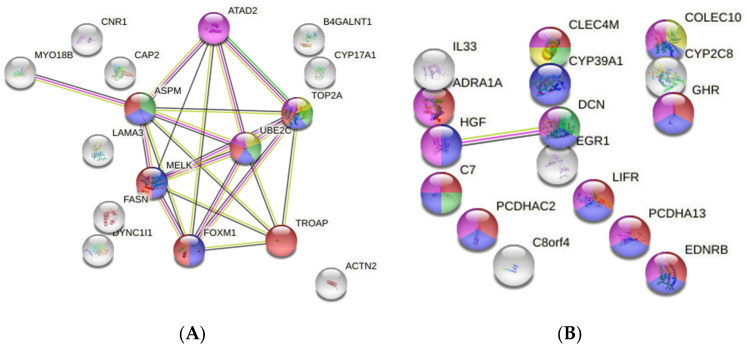
Functional enrichment gene ontology (STRING) local network clusters, color-coded. (**A**) 3 tumor groups with common upregulated DEGs. Blue: mitotic cytokinesis and gastric cancer network 1; red: mitotic spindle checkpoint and mitotic nuclear division; green: G2/M DNA replication checkpoint and gastric cancer network 1; yellow: G2/M DNA replication checkpoint and DNA topoisomerases; purple: Wiki Pathways—gastric cancer network 2. (**B**) 3 tumor groups with common downregulated DEGs. Red: integral component of plasma membrane; blue: signal; green: complement system; yellow: mannose binding; purple: glycoproteins.

Further comparative analysis revealed that there were DEGs present in all three groups or just in two out of three, which we called *“common”/overlapped* (though they presented variable Log2 ratios/fold changes between tumor groups), as follows: 26 upregulated and 17 suppressed genes were found to be common to all three groups (Table 3, common/overlapped HBV, HCV, and non-B, non-C). Figure 1 shows the GO functional enrichment by STRING.

The HBV group had 36 upregulated and 23 downregulated genes that were common to/overlapped with the HCV groups (Supplementary Table S3, data common to HBV and HCV) and another 14 upregulated and 9 downregulated genes common to the non-B, non-C group (Supplementary Table S4, data common to HBV and non-B, non-C).

The HCV group had 59 upregulated and 37 downregulated genes common to/overlapped with the non-B, non-C group (Supplementary Table S5, data common to HCV and non-B, non-C). The HBV, HCV, and non-viral gene lists are shown in Supplementary Tables S6–S8.

Besides the “common”/overlapped genes, in every group we identified also genes that we called “*unique”* because they were differentially expressed in only one of the etiological groups (Table 3).

**Figure 2:**
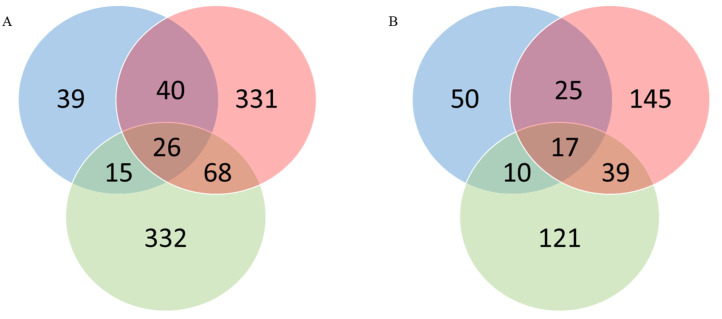
Venn diagrams for (**A**) the upregulated genes in the analyzed etiologies and (**B**) the downregulated genes in the analyzed etiologies. Blue circles: HBV patients; red circles: HCV patients; green circles: non-B, non-C patients).

The overlaps between differentially expressed genes (DEGs—upregulated and downregulated) among tumor types are shown in Figure 2A,B as Venn diagrams.

**Figure 3:**
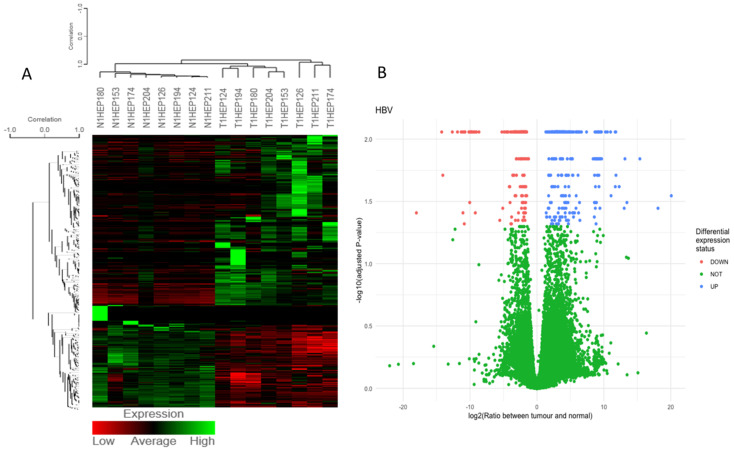

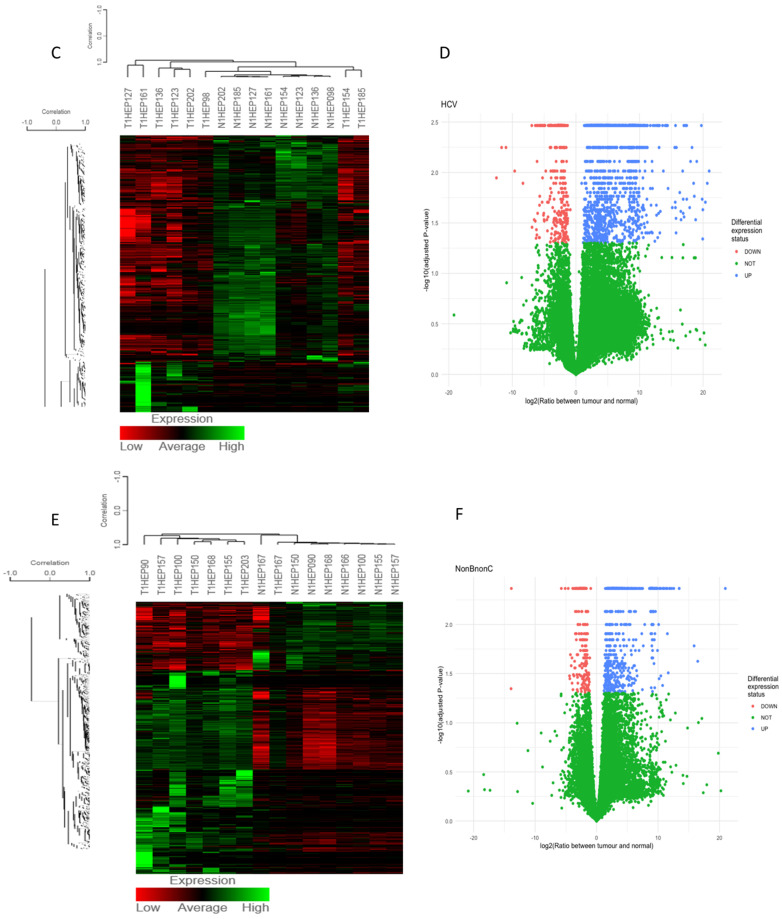
Heatmaps of DEGs in non-tumoral and tumoral tissues and volcano plots showing statistically significant up- (blue) and downregulated genes (red) for: (**A**,**B**) HBV-related tumors, (**C**,**D**) HCV-related tumors, and (**E**,**F**) non-viral-infected tumors. In all volcano plots, genes that did not pass the statistical threshold are shown in green.

For the visualization of gene expression across the samples from the RNA-Seq results, heatmaps and volcano plots were generated (for each tumor group) (Figure 3A–F).

### 3.3. Validation by RT-PCR

**Figure 4:**
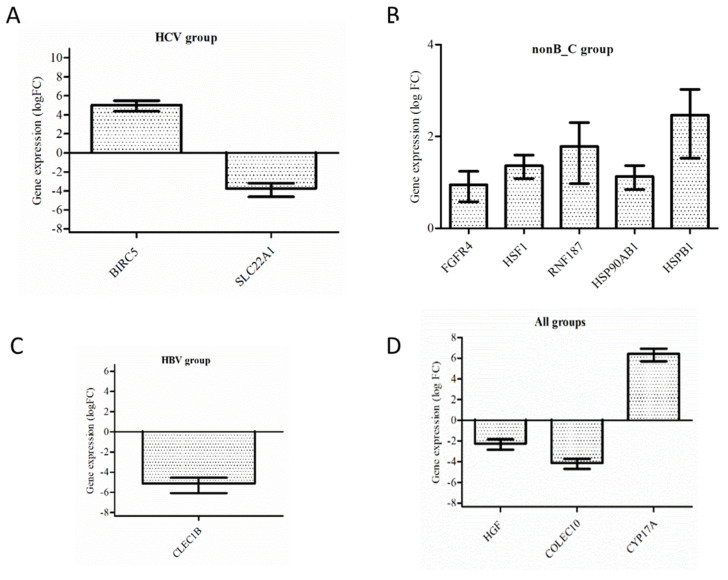
Expression levels for (**A**) *BIRC5* (logFC = 5.02) and *SLC22A1* (logFC = −3.74) in the group of patients with HCV infection; (**B**) *FGFR4*, *HSF1*, *RNF187*, *HSP90AB1*, and *HSPB1* specific to the group of patients without viral infection; (**C**) *CLEC1B* (logFC = −5.12) specific to the group of patients with HBV infection; and (**D**) the common genes *HGF* (log FC = −2.26), *COLEC10* (logFC = −4.13), and *CYP17A* (logFC = 6.42) among all 3 groups. Gene expression values were normalized to b-actin and paired non-tumoral tissues. The relative levels of expression were calculated by the 2^−ΔΔCT^ method (bars indicate standard errors of the means (±SEMs)).

Validation of the gene expression levels obtained by RNA-seq specific to the HCV group (*BIRC5* and *SLC22A1*), the HBV group (*CLEC1B*), and the group without infection (*FGFR4*, *HSF1*, *RNF187*, *HSP90AB1*, and *HSPB1*) was performed using the real-time PCR technique. In addition, common genes among all groups, such as *HGF*, *COLEC10*, and *CYP17A*, were validated. The results indicated that the gene expression data obtained by RNA-seq were consistent with the expression data determined by real-time PCR (Figure 4A–D).

### 3.4. Differential Gene Expression of Solute Carrier Transporters (SLC), ATP Binding Cassette, and Cytochrome Genes

In our comparative study, we identified consistent differential expression (fold change) levels for various SLC, ATP binding cassette, and Cytochrome 450 genes in our three tumor groups (Table 4 and Table 5).

The liver is the basic organ of drug and xenobiotic metabolism, transport, and excretion, and it expresses a variety of enzymes and transporters involved in these processes.

The expression of genes encoding these enzymes can be influenced by liver pathologies, such as viral infection, alcoholic liver disease, primary sclerosis, cholangitis, non-alcoholic fatty liver disease, and hepatocellular carcinoma (HCC) [35]. In the processes associated with carcinogenesis, some of the principal classes of macromolecules (carbohydrates, proteins, lipids, and nucleic acids, etc.) could be modified. Consequently, some genes involved in transport and metabolism might be differentially expressed in tumoral tissues. For example, in our study, the expression of many solute carriers (SLCs) and cytochromes was modified, with different fold changes presented in the three groups of tumors.

Solute carriers (SLCs) represent a major and important class of cellular transporters. They could become valuable targets in cancer therapeutic strategies (e.g., by blocking or activating them) [36,37].

We present here some of the differentially expressed SLCs and cytochromes identified in our groups of tumors compared with data in the literature.

- **SLC44A5** is an intermediate-affinity choline transporter; high expression of *SLC44A5* demonstrates its important role in the development and progression of HCC [38].
- **SLC26A6** belongs to an anion transporter family [39]. *SLC26A6* expression is an independent prognostic factor for HCC, and its upregulation is correlated with poor prognosis [40].
- **SLC38A4** transporter is found predominantly in the liver and transports both cationic and neutral amino acids. Low expression of *SLC38A4* is associated with poor prognosis of HCC patients [41].
- ***SLC22A1*** codes for one of the three organic cation transporters, OCT1, an integral transmembrane protein involved in metabolic processes and detoxification. OCT1/SLCCA1 transports a wide range of substances, such as catecholamines, toxins, and anticancer drugs, and is of pharmaceutical interest [42]. In HCC, the expression of OCT1 is significantly reduced and associated with tumor progression and worse patient survival [43].
- ***CYP17A1*** codes for an enzyme involved in the synthesis of steroid hormones, mineralocorticoids, and glucocorticoids [44]. CYP17A1 is significantly increased in human HCC. *CYP17A1*, as well as *CYP19A1*, is targeted by inhibitors in cancer treatments.
- ***CYP39A1*** studies revealed that total bile acid, total bilirubin, and direct bilirubin were significantly increased in patients with low CYP39A1, and survival analysis of HCC patients indicated that lower *CYP39A1* expression was associated with poorer overall survival. The downregulation of *CYP39A1* is associated with HCC carcinogenesis, tumor differentiation, and poor overall survival. *CYP39A1* may serve as a tumor suppressor gene and a novel biomarker for HCC patients [45].
- ***CYP2C9*** codes for one of the most important drug metabolizing enzymes in humans. Substrates for CYP2C9 include fluoxetine, losartan, phenytoin, tolbutamide, torsemide, S-warfarin, numerous NSAIDs, etc. [46]. In the TCGA database, low expression of *CYP2C8*, *CYP2C9*, and *CYP2C19* in tumor tissue was associated with short median survival [47]. *CYP2C9* could be used as a new biomarker for diagnosis.
- **CYP2C8** plays an important role in oxidative metabolism; the enzyme metabolizes certain chemicals that contain steroids, arachidonic acids, and retinoids and the anionic parts of some drugs.

*CYP2C8*, *CYP2C9*, and *CYP2C19* are downregulated in HCC.

- **CYP2C19** is an enzyme that metabolizes many drugs, such as as clopidogrel (Plavix), omeprazole, mephenytoin, proguanil, diazepam, tamoxifen, amitriptyline, citalopram, lomipramine, etc.

Polymorphism in this gene is associated with variable ability to metabolize drugs. CYP2C19 influences metabolism (particularly the detoxification of carcinogens) as a tumor suppressor [48]. *CYP2C19* is downregulated in HCC [44] and, consequently, detoxification processes are lower and exposure to carcinogens is higher. As a result, carcinogenesis and proliferation easily occur, leading to aggressive manifestations and poor prognosis in HCC [48].

- **CYP3A4** is an important mono-oxygenase that metabolizes xenobiotics (drugs, toxins, etc.) to eliminate them from the body. The enzyme is predominantly found in the liver but also in the intestines. Dowregulation of *CYP3A4* in HCC is associated with poor prognosis. It may be a novel biomarker for HCC [49]. In our study, we identified *CYP3A4* as being dowregulated only in the HCV tumor group.

In our study, the expression of cytochromes was variable between groups, and the most affected was the group of HCV-related tumors.

- **ABC transporters** are mostly exporters; they transport a large variety of molecules using the energy generated by hydrolysis of ATP against their electrochemical gradient. They regulate cellular levels of lipids, ions, xenobiotics, and other small molecules. Studies have revealed that the members of the ABCA subfamily are significantly involved in membrane lipid trafficking (ABCA1, A3, A5, and A9 are detected in almost all tissues) and in cholesterol homeostasis and they have been associated with some inherited diseases [50]. In hepatocellular cancer, *ABCA8* and *ABCA9* are downregulated, and HCC patients had significantly shorter survival times [51].

Overexpression of many ABC transporters mediates multidrug resistance (MDR) in cancer. In hepatocellular carcinoma, MDR is mediated by ABCB1, ABCB5, ABCC1, ABCC2, and ABCG2 [52]. Beyond the augmentation of the capacity to efflux various therapeutic cytotoxic drugs, the ABC dysregulated genes are being increasingly associated with cancer development and evolution processes (angiogenesis, apoptosis, proliferation, invasion, metastasis, etc.) [50]. *ABCB5* has been reported to be overexpressed in HCC and as being associated with chemoresistance, cancer stemness properties, and poor recurrence-free survival [53].

A recent study revealed the upregulation of *ABCF1* in drug-selected chemoresistant HCC cells. ABCF1 is a hepatic oncofetal protein that modulates migration, epithelial–mesenchymal transition (EMT), and cancer stemness properties and is considered a novel potential therapeutic target for HCC treatment [54].

### 3.5. Differential Expression of Cancer-Testis-Specific Genes

A series of CT genes are upregulated in hepatocellular carcinoma, involved in cell-cycle regulation, cancer progression, and signaling pathways and can serve as biomarkers for diagnostics, prognostics, and treatment [55,56].

In our study, we identified a series of dysregulated cancer testis DEGs (Table 6).

- **The Melanoma Antigen Gene** (MAGE) family was reported to participate in the progression of multiple cancers in humans, including HCC [57].
- **ACTL8** is a member of the sugar kinase/heat shock protein 70/actin superfamily. It is upregulated and contributes to invasion and metastasis in many cancers [58,59,60]. *ACTL8* could be involved in epithelial cell differentiation and may be a potential prognostic marker and novel therapeutic target.
- **ATAD2** (ATPase family AAA domain-containing 2) participates in carcinogenic processes. *ATAD2* is overexpressed in various human malignancies, including HCC; it is a potential proliferation marker for liver regeneration and is a poor prognostic marker for hepatocellular carcinoma after curative resection [61,62].

### 3.6. Differential Expression of Heat Shock Proteins and Heat Shock Factors

The heat shock proteins (HSPs) or stress proteins are cellular constitutive, ubiquitous, highly evolutionarily conserved molecules that have cytoprotective properties; they are the main basic elements of the cellular proteoprotection system. HSPs have multiple functions in physiological conditions: chaperoning functions, disaggregation and possibly even refolding of damaged proteins, and, more important, protection of newly synthesized proteins and help with their folding (in an ATP-dependent manner) into functional forms. HSP expression might be induced by many stress factors, e.g., thermic stress (heat shock), chemical stress (heavy metals), oxidative stress (free radicals), denatured proteins, antibiotics, immunosuppressive drugs, hypoxia, nutritional inadequacy, and pathological conditions (inflammation, fever, viral and bacterial infections, carcinogenesis) [63,64,65]. Deregulation of stress gene expression is associated with various human diseases, including malignancies. Heat shock proteins (HSPs) are found to be overexpressed in tumor cells, where they protect oncogenic proteins. Stress induction of HSPs plays a crucial role in tumorigenesis, metastasis, and therapeutic resistance [66].

Our analysis identified significant dysregulation of stress proteins and heat shock factors, thus confirming perturbed metabolic homeostasis in HCC (Table 7).

- **HSF4 (Heat Shock Transcription Factor 4)**

HSF4 is a member of the heat shock transcription factor family and is expressed in human tissues. Dysregulation of HSF4 expression might induce carcinogenesis. *HSF4* was found to be upregulated in HCC tissues and, more important, elevated in primary HCC tissues derived from recurrent patients; consequently, *HSF4* was considered an independent poor-prognosis predictor after resection [67,68]. Our analysis identified *HSF4* as being upregulated only in the HCV tumor group.

- HSF1, HSP70, HSP90, and HSPB1 are further described as hub genes.

### 3.7. Functional Enrichment Analysis of DEGs

**Figure 5:**
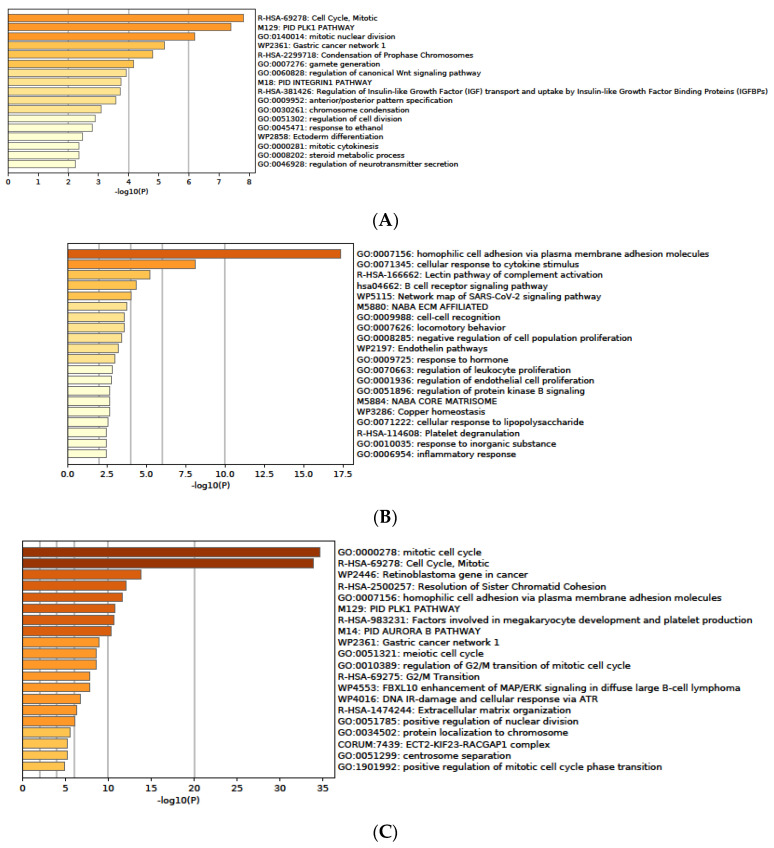

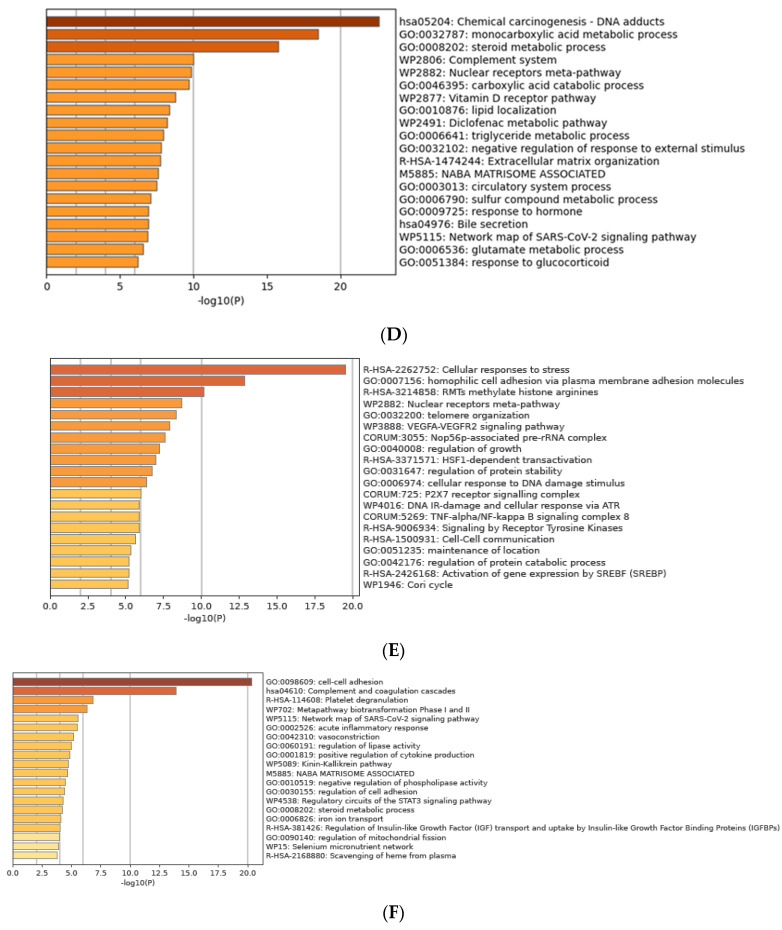
Enrichment heat maps for selected GO DEGs: (**A**) **upregulated in the HBV** tumor group; (**B**) **downregulated in the HBV** tumor group; (**C**) **upregulated in the HCV** tumor group; (**D**) **downregulated in the HCV** tumor group; (**E**) **upregulated in the non-B, non-C (non-viral)** tumor group; and (**F**) **downregulated in the non-B, non-C (non-viral**) tumor group. Bar graphs of enriched terms across input gene lists colored according to *p*-values. The terms shown in each plot are enumerated in the text below. (**A**) ***Selected GO processes and pathways enriched with DEGs upregulated in the HBV-related tumor group*:** R-HSA-69278: Cell cycle, Mitotic; M129: PID PLK1 PATHWAY; GO:0140014: Mitotic nuclear division; WP2361: Gastric cancer network 1; R-HSA-2299718: Condensation of prophase chromosomes; GO:0007276: Gamete generation; GO:0060828: Regulation of canonical Wnt signaling pathway; R-HSA-381426: Regulation of insulin-like growth factor (IGF) transport and uptake by insulin-like growth factor binding proteins (IGFBPs); GO:0030261: Chromosome condensation; GO:0051302: Regulation of cell division; GO:0045471: Response to ethanol; GO: WP2858: Ectoderm differentiation; GO: 0000281: Mitotic cytokinesis; GO:0008202: Steroid metabolic process. (**B**) ***Selected GO processes and pathways enriched with DEGs downregulated in the HBV-related tumor group***: GO:0007156: Homophilic cell adhesion via plasma membrane adhesion molecules; GO:0071345: Cellular response to cytokine stimulus; M5885: NABA MATRISOME ASSOCIATED; R-HSA-166662: Lectin pathway of complement activation; hsa04662: B cell receptor signaling pathway; WP5115: Network map of SARS-CoV-2 signaling pathway; GO:0009988: Cell–cell recognition; GO:0007626: Locomotory behavior; GO:0008285: Negative regulation of cell population proliferation; WP297: Endothelin pathways; GO:0009725: Response to hormone and others. (**C**) ***Selected GO processes and pathways enriched with DEGs upregulated in the HCV-related tumor group*:** GO:0000278: Mitotic cell cycle; WP2446: Retinoblastoma gene in cancer; R-HSA-2500257: Resolution of sister chromatid cohesion; GO:0007156: Homophilic cell adhesion via plasma membrane adhesion molecules; M129: PID PLK1 PATHWAY; R-HSA-983231: Factors involved in megakaryocyte development and platelet production; M14: PID AURORA B PATHWAY; WP2361: Gastric cancer network 1; GO:0051321: Meiotic cell cycle; GO:0010389: Regulation of G2/M transition of mitotic cell cycle; R-HSA-69275: G2/M Transition; R-HSA-1474244: Extracellular matrix organization; GO:0051785: Positive regulation of nuclear division; CORUM:7439: ECT2-KIF23-RACGAP1 complex; GO:0051299: Centrosome separation; GO:1901992: Positive regulation of mitotic cell cycle phase transition; WP2363: Gastric cancer network 2; WP3888: VGFA-VGFR signaling pathway and others. (**D**)*** Selected GO processes and pathways enriched with DEGs downregulated in the HCV-related tumor group*:** hsa05204: Chemical carcinogenesis DNA adducts; GO:0032787: Monocarboxylic acid metabolic process; GO:0008202: Steroid metabolic process; WP2806: Complement system; WP2882: Nuclear receptors meta-pathway; GO:0046395: Carboxylic acid catabolic process; GO:0010876: Lipid localization; GO:0006641: Triglyceride metabolic process; GO:0032102: Negative regulation of response to external stimulus; R-HSA-1474244: Extracellular matrix organization; GO:0003013: Circulatory system process; GO:0006790: Sulfur compound metabolic process; GO:0009725: Response to hormone; hsa04976: Bile secretion; WP5115: Network map of SARS-CoV-2 signaling pathway; GO:0006536: Glutamate metabolic process; GO:0051384: Response to glucocorticoid; GO:0038172: Interleukin-33-mediate signaling pathway; GO:0043549: Regulation of kinase activity; Hsa00650: Butanoat metabolism; HSA9006934: Signaling by tyrosine kinases; GO:0031638: Zymogen activation; Hsa00232: Caffeine metabolism; Hsa05200: Pathways in cancer; GO:0098609: Cell–cell adhesion; GO:0015909: Long-chain fatty acid transport; WP1533: Vitamin B12 metabolism; GO:0015849: Organic acid transport; GO:0016042: Lipid catabolism process; GO:0008285: Negative regulation of cell population proliferation; GO:0002697: Regulation of immune effector process and others. (**E**) ***Selected GO processes and pathways enriched with DEGs upregulated in the non-viral-infected (non-B, non-C) tumor group*:** R-HSA-2262752: Cellular responses to stress; GO:0007156: Homophilic cell adhesion via plasma membrane adhesion molecules; WP2882: Nuclear receptors meta-pathway; R-HSA-324858: RMTs methylate histone arginine; GO:0032200: Telomere organization; WP3888: VEGFA-VEGFR2 signaling pathway; CORUM:3055: Nop56p-associated pre-rRNA complex; GO:0040008: Regulation of growth; R-HSA-3371571: HSF1-dependent transactivation; GO:0031647: Regulation of protein stability; GO:0006974: Cellular response to DNA damage stimulus; WP4016: DNA IR damage and cellular response via ATR; R-HSA-9006934: Signaling by receptor tyrosine kinases; GO:0051235: Maintenance of location; GO:0042176: Regulation of protein catabolic process; GO:0033365: Protein localization to organelle; WP1946: Cori cycle; WP314: Fas ligand pathway and stress induction of heat shock proteins; GO:0010506: Regulation of autophagy; R-HSA-1428517: TCA cycle and respiratory electron transport; R-HSA-1428517: TCA cycle and respiratory electron transport; GO:0010506: Regulation of autophagy; R-HSA-2426168: Activation of gene expression by SREBF (SREBP); GO:0000278: Mitotic cell cycle; R-HSA-382551: Transport of small molecules; R-HSA-5653656: Vesicle-mediated transport; GO:0042176: Regulation of protein catabolic process and others. (**F**) ***Selected GO processes and pathways enriched with DEGs downregulated in the non-viral-infected (non-B, non-C) tumor group***: GO:0098609: Cell–cell adhesion; hsa04610: Complement and coagulation cascades; R-HSA-114608: Platelet degranulation; WP702: Meta-pathway biotransformation Phase I and II; WP5115: Network map of SARS-CoV-2 signaling pathway; GO:0002526: Acute inflammatory response; GO:0001819: Positive regulation of cytokine production; GO:0060191: Regulation of lipase activity; M5885: NABA MATRISOME ASSOCIATED; GO:0030155: Regulation of cell adhesion; GO:0008202: Steroid metabolic process; GO:0006826: Iron ion transport; R-HSA-381426: Regulation of insulin-like growth factor (IGF) transport and uptake by insulin-like growth factor binding proteins (IGFBPs); GO:0072593: Reactive oxygen species metabolic process; GO:002920: Regulation of humoral immune response; Hsa05200: Pathways in cancer; WP4538: Regulatory circuits of the STAT3 signaling pathway; WP5089: Kinin–kallikrein pathway and others.

Here, we present the most relevant GO processes and pathways enriched with DEGs as found in our study for the HBV (Figure 5A,B), HCV (Figure 5C,D), and non-B, non-C groups respectively (Figure 5E,F).

### 3.8. Identification of Regulator Genes, Hub Genes, and Moonlighting Genes

Regulator genes (or regulatory genes) are genes that regulate the expression of one or more structural genes. Their function is to ensure that gene products, such as enzymes, structural proteins, and RNA molecules, are synthesized when they are needed and in the proper amounts. Regulator genes also allows cells to react quickly to changes in their environments [69].

Moonlighting proteins comprise a class of multifunctional proteins which singly perform multiple physiologically relevant biochemical or biophysical functions that are not due to gene fusion, multiple RNA splice variants, or pleiotropic effects (e.g., soluble enzymes that also bind to DNA or RNA to regulate translation or transcription) [70,71].

Numerous studies have demonstrated that individual proteins can moonlight, meaning that they can have multiple functions based on their cellular or developmental contexts.

Moonlighting may be particularly relevant in the context of human disease, especially in cancer [72].

**Figure 6:**
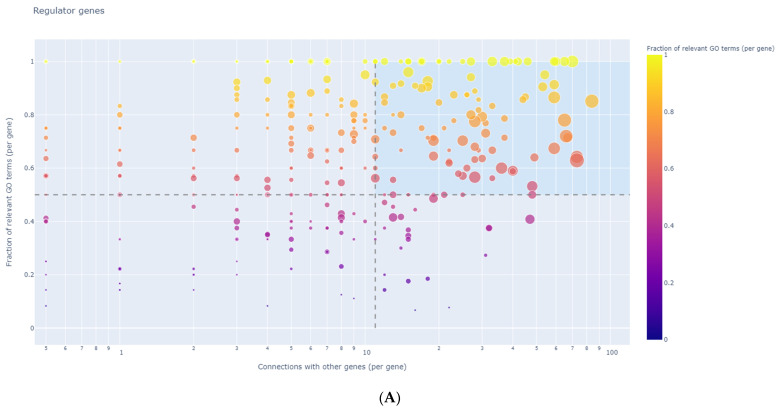

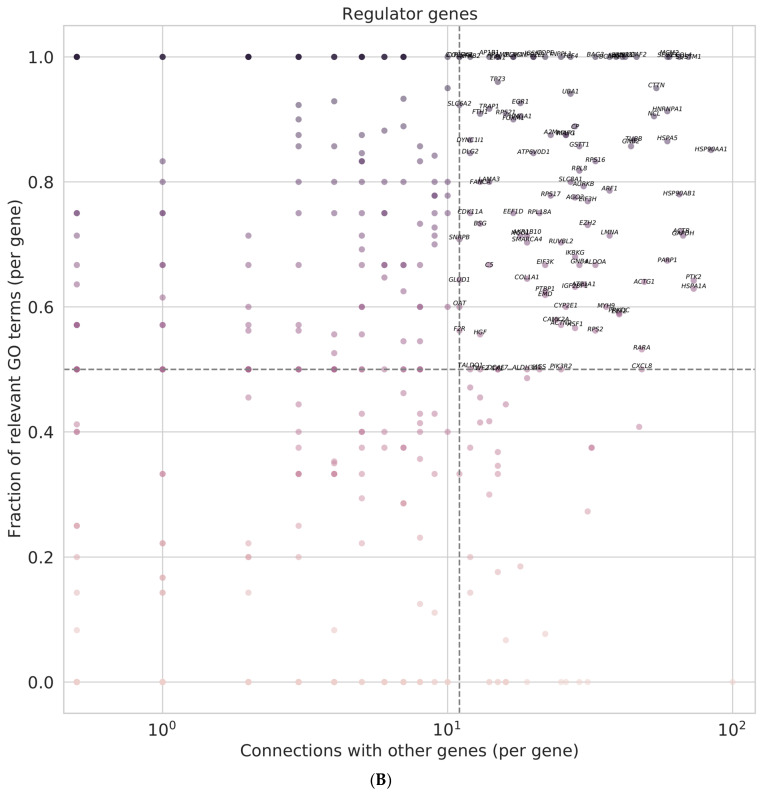

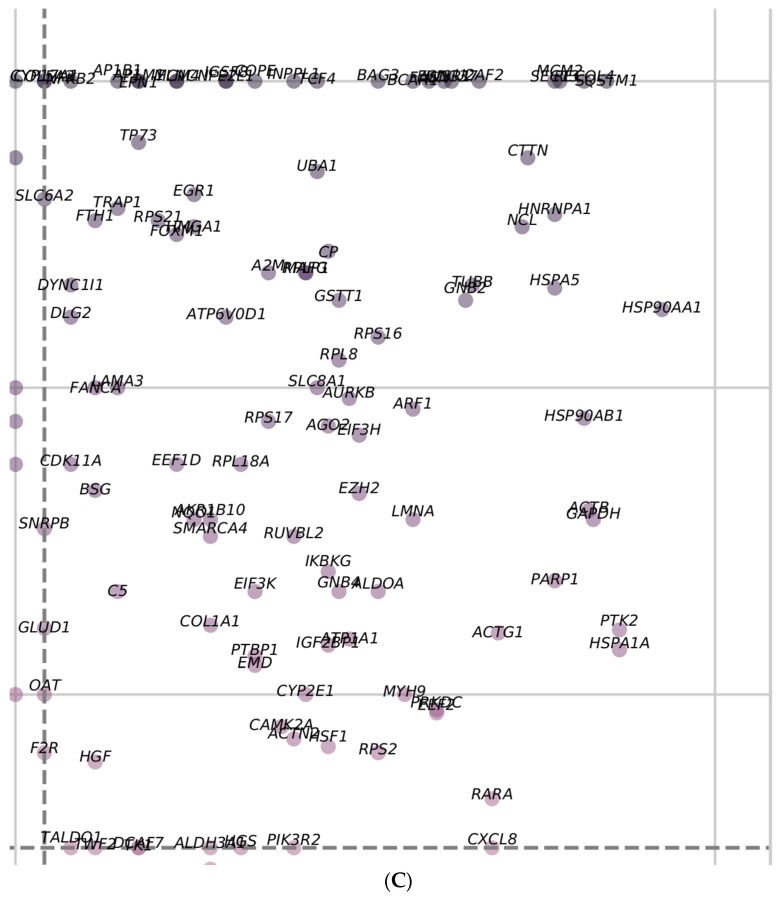
Non-viral regulator genes. (**A**) Scatter plot with DE genes as data points showing the GeneWalk fraction of relevant GO terms over the total number of connected GO terms. These have a large gene connectivity and a high fraction of relevant GO annotations. Circle size indicates differential expression significance strength; [−log_10_ (*p*-adjust)] and color hue with min_f. (**B**) Print-screen of GeneWalk (**C**) Magnification of upper-right corner of image B. (The complete gene list is given in Supplementary Table S10).

**Figure 7:**
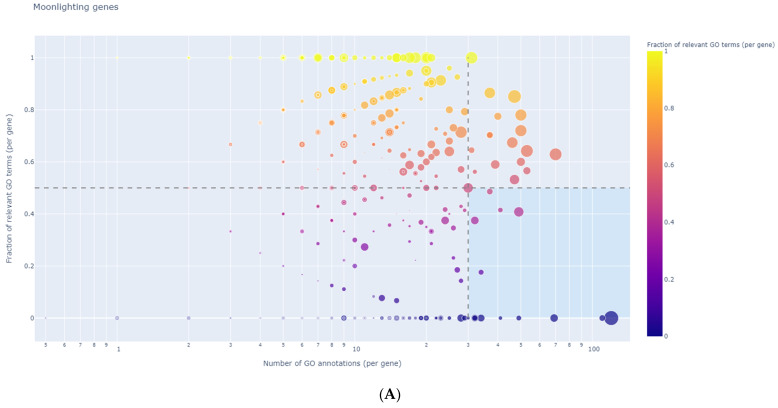

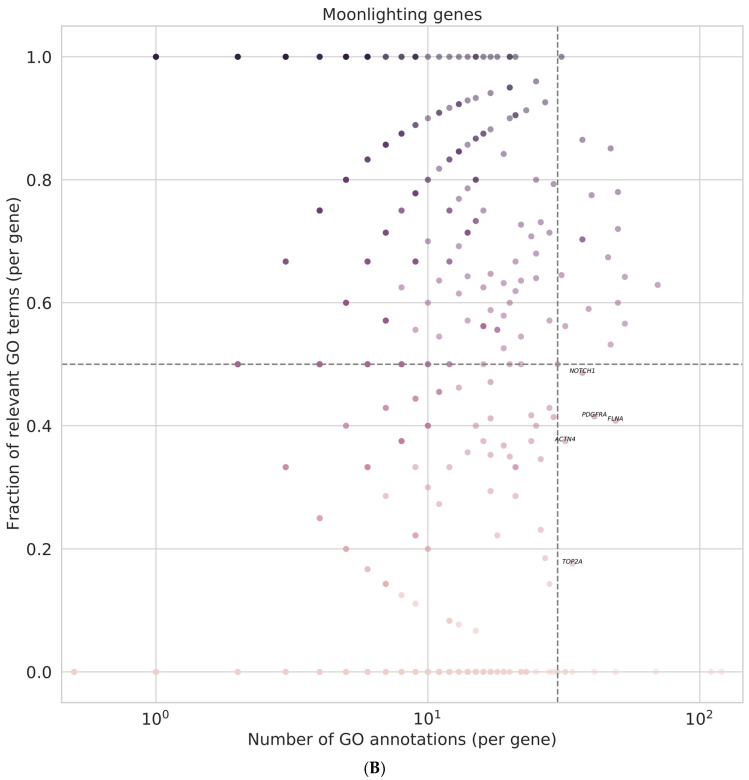

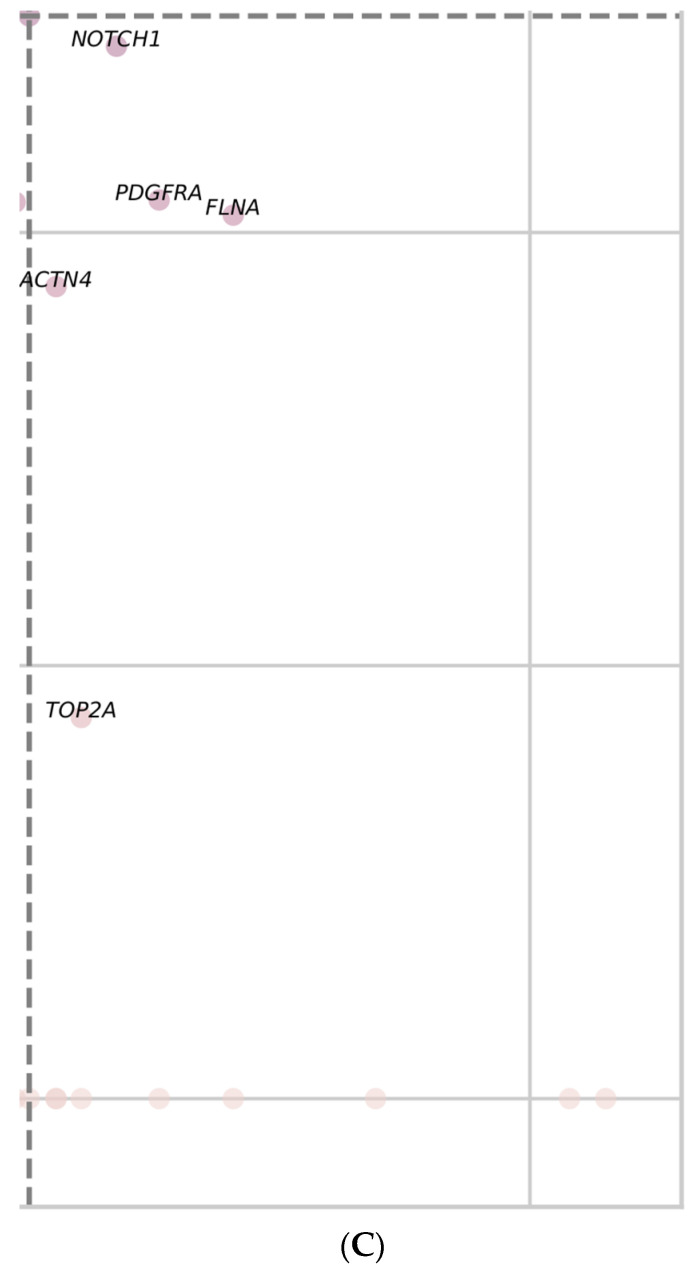
Non-viral moonlighting genes. (**A**) Scatter plot. (**B**) Scatter plot of genes located in the bottom-right area. (**C**) Magnification of lower-right corner of image B.

In our study, the DEG analysis using GeneWalk showed 145 regulator genes in the HCV-related tumor group and 1 moonlighting gene (*ECT2*). In the non-viral-infected group of tumors, 106 regulator genes (graphic representations in Figure 6A,B) and 5 moonlighting genes (*ACTN4*, *FLNA*, *NOTCH1*, *TOP2A*, and *PDGFRA*) were detected (Figure 7A,B). The regulator genes were identified as those with a wide connectivity to other input genes and high fractions of relevant GO annotations. The list of regulator genes is available in Supplementary Table S9 (HCV group) and Supplementary Table S10 (non-viral group). Moonlighting genes were identified as those with many GO annotations of which only a small fraction are relevant [25]. No significant regulatory or moonlighting genes were identified in our HBV-related tumor group.

From the regulator gene list, we further selected the most significant hub genes using the following thresholds: global_padj < 0.1; ncon_gene ≥ 50; and ncon_go ≥ 50. The hub genes and their connectivity degrees are presented in Table 8 and Supplementary Figure S4, respectively, for the non-viral group and in Table 9 and Supplementary Figure S3, respectively, for the HCV group.

We further describe the functional role and involvement of some identified hub genes in normal and pathological conditions.

#### 3.8.1. Non-Viral Group HUB Genes and Proteins

- HSF1 (Heat Shock Transcription Factor 1). In vertebrates, the prototype of heat shock transcription factor is HSF1, which mediates the induction of heat shock gene expression in response to environmental stress [73].

As a mitotic regulator, HSF1 is a major contributor to cancer morbidity. It allows a series of cell-level tumorigenic processes (deregulation of cell-cycle progression, increased cell survival, etc.) and modulates tumor-level tumorigenic features (invasion, angiogenesis, and metastasis) [74]. HSF1 participates in the initiation, development, and progression of various cancers, including hepatocellular carcinoma. HSF1 exhibits high expression in HCC and in other malignancies [75,76,77,78].

- **HSPB1/HSP27** is a stress-inducible chaperone which belongs to the small heat shock protein family [79]. HSPB1 has multiple functions and regulates many cellular processes, such as cytoskeleton organization, maintenance of cellular proteostasis, inhibition of apoptosis, modulation of autophagy induction of resistance to anticancer drugs, etc. [80,81,82]. Numerous studies have revealed that HSPB1 promotes tumorigenesis [66,83,84] and is dysregulated in different malignancies. *HSPB1* is upregulated in HCC, and it was identified as a hub gene [85]. The overexpression of *HSPB1* was associated with a worse prognosis in HCC patients and it was considered a possible target of immunotherapy in HCC [86].
- **HSP90AA1:** The heat shock protein 90 (HSP90) family perform a large number of cellular regulatory functions in normal and pathological processes. In vertebrates, the two major paralog isoforms are HSP90AA1 and HSPAB1 [87].

Hsp90 is an essential element for malignant transformation and progression as a cancer supporter that assists and interacts with oncogenic proteins [88]. HSP90 acts as an important regulator of autophagy that leads to inhibited apoptosis and increased drug resistance [89]. HSP90AA1 suppression results in increased sensitivity to chemotherapy [90].

Hepatocellular HSP90 is positively involved in HCC development by increasing liver cancer cell invasion, inhibiting cancer stem cells, apoptosis, etc. [90]. It is considered a potential biomarker for detection/screening, prognostics, and supervision of human hepatocarcinogenesis [91] and a valuable target in cancer therapy [92].

- ***HSPA1A*** gene codes for molecular chaperons proteins, belonging to the HSP70 Heat Shock Protein Family A. HSPA1A is a major stress-induced member, having crucial roles in protein homeostasis and cell survival [93].

In oncogenesis, HSPs play an essential, facilitating role through the accumulation of overexpressed and mutated oncogenes through their cytoprotective functions (inhibition of apoptosis, as well as HSP27) [81,94] and have multiple implications for the hallmarks of cancer [95].

*HSP70* is overexpressed in different types of cancers; it was identified as a molecular marker of early hepatocellular carcinoma [96,97,98,99,100] and correlated with unfavorable overall survival in HCC patients [81].

- **HSPA5** (GRP78, BiP) is a chaperone protein constitutively expressed in the endoplasmatic reticulum (ER); GRP78 maintains normal ER functions and is the principal regulator of cellular response to ER stress [101,102]. A series of studies have demonstrated that GRP78/HSPA5 is anti-apoptotic and has a critical cytoprotective role in oncogenesis (protects tumor cells from ER stress) [103].

It was demonstrated that GRP78 is a novel obligatory component of autophagy in mammalian cells [103,104,105]. GRP78 is involved in tumor proliferation, survival, tumor angiogenesis, metastasis, and drug resistance, and overexpression of GRP78 was observed in the progression of many human cancers, including hepatocellular carcinoma [105,106,107].

- **ACTB** (Beta-actin) is a highly conserved cytoskeleton structural protein generally upregulated and involved in the development and metastasis of various cancers, including HCC [108,109].
- **ALDO A** (Aldolase A) is an important member of the glucose metabolism enzyme family. Glucose metabolism dysfunction is one of the most important characteristics of cancers. High expression of *ALDOA* is associated with the initiation and progression of many cancers. *ALDOA* contributes to moonlighting functions; under hypoxia, ALDO A regulates cell proliferation, invasion, and apoptosis, being an essential driver in HCC [110,111].
- **GAPDH** (Glyceraldehyde-3-phosphate dehydrogenase) is an essential regulator of glycolysis overexpressed in numerous cancers, including HCC, and enabling tumor progression. GAPDH is functionally active in the nucleus, cytoplasm, and plasma membrane and also carries out numerous, non-glycolytic ‘‘moonlighting’’ functions. Glycolytic enzymes have gained increasing attention as potential anticancer therapeutic targets.
- **CTTN** (Cortactin) is an important actin-binding and assembly protein involved in cytoskeletal regulation. It is found at sites of dynamic actin assembly, in cellular protrusions, such as invadopodia, and is associated with cell motility and invasion. CTTN enhances cell migration, invasion, and tumor cell metastasis and is overexpressed in many cancers, including HCC [112,113].

#### 3.8.2. HCV Group HUB Genes and Proteins

- **ACTN2**-Alpha actinin is an actin-binding cytoskeletal protein. The alpha actinin isoform, which is concentrated in the cytoplasm, is thought to be involved in metastatic processes.

Studies have revealed that *ACTN2* overexpression in HCC stimulates invasion abilities by enhancing cellular motility, demonstrating a pro-metastatic role in tumorigenesis [114]. ACTN2 was also cited as an HCC hub gene in other studies [115].

- **ANXA** 2-Annexin A2 belongs to a protein family (annexins) whose members bind anionic phospholipids in a calcium-dependent manner and have the ability to aggregate membranes.

*ANXA 2* is overexpressed in many human cancers, including hepatocellular carcinoma (HCC), and has multiple regulatory roles and is correlated with proliferation, cell migration, adhesion, angiogenesis, apoptosis, etc. [116].

Upregulated *ANXA2* in HCC plays an important role in tumor immune escape and is proposed as a target in cancer treatment [117,118].

- **AURKA** (Aurora Kinase A) is a serine/threonine kinase that plays essential roles in regulating cell division during mitosis. Abnormal activity of *AURKA* promotes tumorigenic progression [119] and is highly expressed in various cancers, including HCC [120]. It might be a reliable predictor of early-stage HCC, a crucial biomarker for HCC development, and a reliable target for cancer therapy [121,122,123].
- **AURKB** (Aurora Kinase B) is a serine/threonine fundamental kinase (as is AURKA) involved in the regulation of cell mitosis, especially in chromosomal segregation [124]. The dysregulation of aurora kinase genes has been reported in many cancers. The expression of AURKB was found to be higher in HCC than in a control and was consistently correlated with patient tumor stage [125].
- ***BRCA1*** (BRCA1 DNA Repair Associated) encodes a nuclear phosphoprotein that plays an important role in the correct repair of damaged DNA and maintaining genomic stability. *BRCA1* is overexpressed in many type of cancers, including HCC, where its expression correlates with immune cell infiltration [125,126].
- **CCNB1** (Cyclin B1) belongs to the cyclin family. Eukaryotic cell-cycle progression is regulated by cyclin-dependent kinases (Cdks) and their regulatory cyclin subunits. Cyclin/Cdk complexes activate transcription, enable DNA replication, and catalyze mitosis [127,128]. Overexpression of *CCNB1* can promote proliferation in human HCC cells and was identified as a hub gene in HCC in others studies as well [129].
- **CDK1** (Cyclin Dependent Kinase 1) is a member of the serine/threonine protein kinase family and has a crucial role in cell proliferation initiating mitosis [127,128]. Overexpression of *CDK1* has been observed in different type cancers, including hepatocellular carcinoma [130,131], where it is correlated with poor OS. Moreover, expression levels of *CDK1*, *CCNB1*, and *CCNB2* were positively correlated with infiltrating levels of CD4^+^ T cells, CD8^+^ T cells, neutrophils, macrophages, and dendritic cells in HCC [132,133]. Other studies also identified *CDK1* and *CCNB1 as Hub genes for HCC* [129].
- ***CYP3A4*** (Cytochrome P450 Family 3 Subfamily A Member 4) codes for an important mono-oxygenase, metabolizing xenobiotics (drugs, toxins, etc.) to eliminate them from the body [134]. The enzyme is predominantly found in the liver but also in the intestines. Dowregulation of *Cyp3A4* in HCC was associated with poor prognosis [135].
- **CYP1A2** (Cytochrome P450 family 1 Subfamily A Member 2) is the major hepatic isoform of the human CYP1A subfamily. It is involved in the clearance mechanisms for important drugs (tizanidine, theophylline, clozapine, caffeine, etc.) and participates in the biotransformation processes of different procarcinogens. *CYP1A2* is markedly decreased in primary HCC tumors and is an independent predictor for post-surgical recurrence in early-stage HCC patients [136,137].
- **EGF** (Epidermal Growth Factor) is a growth factor secreted by tumors and inflammatory cells in the tumor microenvironment. EGF binds to a transmembrane glycoprotein, its receptor EGFR (epidermal growth factor receptor), and activates/triggers regulatory signal transduction pathways of proliferation, differentiation, survival, and migration.

Overexpression of *EGF* was reported in many human cancers, including HCC. *EGF* is highly expressed in HCC and facilitates DNA synthesis, regeneration, tumor growth and progression, and promotes metastasis [138,139].

- **TERT** (Telomerase reverse transcriptase) is the catalytic subunit of telomerase. In early stages of cancer, because of the increased cell proliferation, telomeres are shortened, but with tumor progression telomerase is reactivated and the capacity for infinite cell division (immortalization) is gained [140]. TERT upregulation is a critical event in hepatocarcinogenesis. It has been shown that TERT expression increases in hepatocyte cultures after overexpression of HCV core protein as compared to normal human liver and uninfected cells [141]. The HCV core protein is a transcriptional activator of a number of host genes [142] and it has been suggested that it interferes with telomerase expression and might be essential for malignancy [141].
- ***TOP2A*** (DNA Topoisomerase II Alpha) is a gene that encodes a nuclear enzyme which catalyzes the transient breaking and rejoining of two strands of duplex DNA (which allows the strands to pass through one another). It is involved in processes that occur during DNA transcription and replication, such as relief of torsional stress, chromosome condensation, and chromatid separation [143]. *TOP2A* is involved in many cancers, being a prognostic biomarker and potential therapeutic target for bladder cancer, lung adenocarcinoma, prostate cancer, colon cancer, breast cancer, and HCC [144,145].

### 3.9. Analysis of Tumor Immune Infiltrate

Based on the transcriptomic differences shown between etiological tumor groups analyzed in our study, we further examined the tumor immune infiltrate transcriptomic profiles.

In general, in HCC the immune landscape of tumoral tissue is significantly altered compared to the normal one; consequently, the evaluation of immune infiltration patterns contributes to the establishment of new HCC immunotherapy strategies in personalized medicine.

The immune infiltrate in HCC samples was investigated using Immunome [27]—a compendium of immune cell markers preferentially expressed in the majority of immune subtypes infiltrating tumors.

We first compared the tumoral tissues with the normal samples, regardless of HBV and HVC infection, and obtained genes significantly differentially expressed (adjusted *p*-value < 0.005) with a fold change greater than 2.5. Among these genes, markers preferentially expressed in Th2 cells and eosinophils had significantly higher expression in tumors compared to normal samples (Supplementary Figure S5A). In contrast, in normal tissue markers of B cells, Th1, TFH, iDC, NK cells, mast cells, and macrophages had significantly higher expression than in tumors. Similar results were observed in the TCGA cohort (*n* = 421 patients; Supplementary Figure S5B).

**Figure 8:**
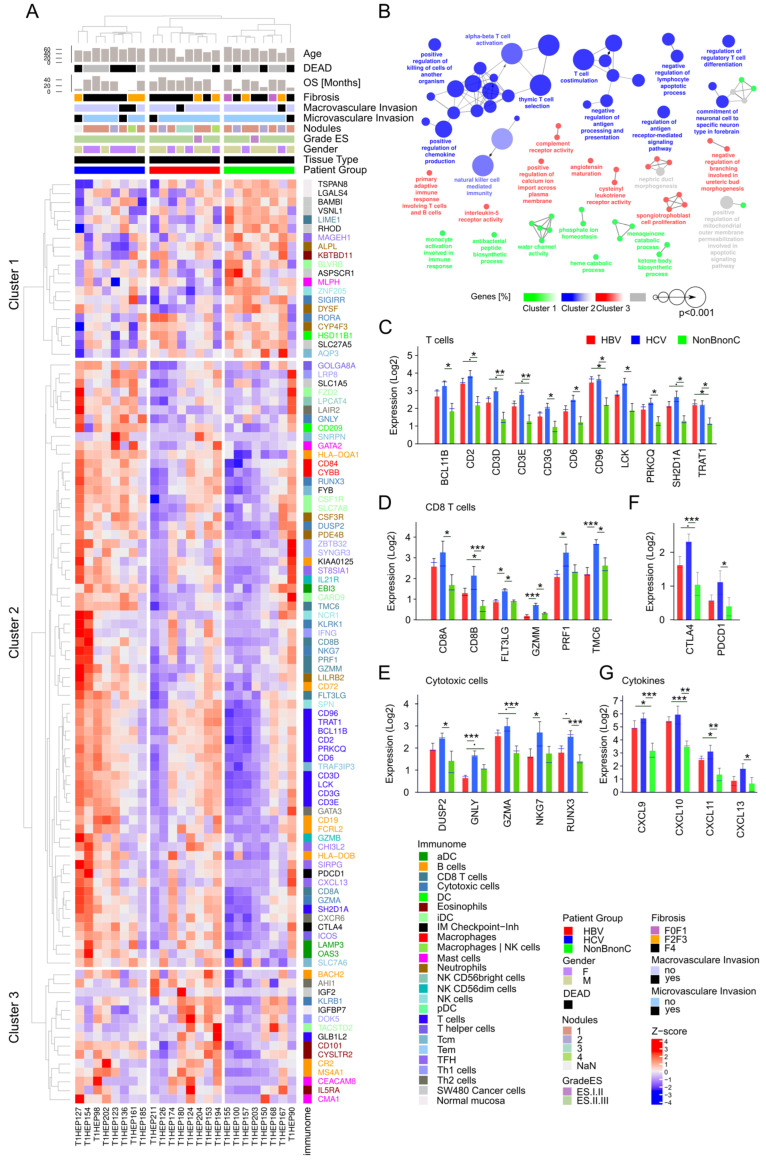
Immune profile analysis. (**A**) Immunome of HBV (red), HCV (blue), and non-B, non-C (green) tumors. Genes with a fold change >1 and adjusted *p*-value < 0.05 are shown. Data were Z-score-normalized and hierarchically clustered (Euclidean distance). Z-score spans between −4 (blue) and 4 (red). A color code was used to mark genes preferentially expressed according to immune cell types. Clinical parameters: Age, DEAD, OS (Months), Fibrosis, Macrovascular invasion, Microvascular invasion, Nodules, Grade ES, Gender, Tumor type are indicated at the top. (**B**) ClueGO functional analysis of gene clusters from (**A**): Cluster 1 with high expression in non-B, non-C tumors (green), Cluster 2 (HCV, blue), and Cluster 3 (HBV, red). GO terms from levels 3-8 were included (Cluster 2: 1 gene, 6%; Cluster 1 and Cluster 3: 1 gene, 4%). Fusion was applied. Network shows GO terms after multiple testing correction. The size of the nodes shows the significance of the terms. Nodes are colored based on the proportions of associated genes from Cluster 1, Cluster 2, or Cluster 3. Equal proportions of genes from the three clusters are shown in gray. Bar charts showing the expression levels of (**C**) T cells (**D**) CD8 T cells (**E**) cytotoxic cells, (**F**) immune checkpoint markers, and (**G**) cytokine markers in HBV (red), HCV (blue), and non-B, non-C (green) tumors. Differential expression analysis was performed with Limma-Voom. Significance levels are shown as: * *p* < 0.05, ** *p* < 0.01, *** *p* < 0.005.

In the next step, we analyzed the Immunomes from tumors with HBV, HCV, and tumors without viral infection. Immune gene data were extracted and clustered (Figure 8A, Supplementary Table S11) and the biological roles of genes with the highest expression in HBV (Cluster 3), HCV (Cluster 2), or tumors without viral infection (Cluster 1) were investigated with ClueGO [29] and CluePedia [30]. An over-representation of T-cell-, cytotoxic-, and natural-killer-cell-related GO terms was observed for Cluster 2 genes (Figure 8B, Supplementary Table S11). Many of these genes are known to be involved in protein–protein interactions leading to the activation or inhibition of expression or to immune cell activation, while others are chemokine–receptor binding pairs (Supplementary Figure S5C). Cluster 3 genes were associated with GO terms involved in primary adaptive immune response and complement receptor activity, while Cluster 1 genes were associated with monocyte activation involved in immune response and other metabolism-related terms.

As previously reported [146], a similar immune profile was observed for HBV and HCV tumors (Figure 8C). Many immune genes had significantly higher expression in these tumors than in tumors without viral infection, as was illustrated for markers of T cells, CD8 T cells, and cytotoxic cells (Figure 8C–E).

The immune checkpoint inhibitors PDCD1 and CTLA4 were also part of Cluster 2 and had significantly higher expression in HCV compared to non-B, non-C tumors (Figure 8F). A similar trend was observed for HBV tumors.

The expression of cytokines, such as CXCL9, CXCL10, CXCL11 and CXCL13, that attract specific immune cells at the tumor site was significantly higher in HBV and HCV tumors than in non-B, non-C tumors (Figure 8G).

## 4. Discussion

Our study analyzed three original NGS whole-transcriptome datasets and revealed consistent differential gene expression between non-tumoral and tumoral tissues, including 222 DEGs (120 upregulated and 102 downregulated) in HBV-related tumors, 691 DEGs (465 upregulated and 226 downregulated) in HCV-related tumors, and 628 DEGs (441 upregulated and 187 downregulated) in non-viral-infected tumors. In the HBV group, a smaller number of DEGs were consistently identified. We identified *common* (overlapped) DEGs (present in all three etiological groups or in two of three) and *unique* DEGs (present only in one of the three groups) in all three analyzed groups.

Further analysis showed variable fold change values for common DEGs between the tumor groups.

In the HCV-related group, we identified a higher number of dysregulated DEGs as SLC (solute carrier), cytochrome p450, cancer testis, oncogenes, tumor suppressor genes, etc.

SLC and cytochrome (CYP) genes are involved in drug absorption, distribution, metabolism, and excretion (ADME). Their correlated actions control liver drug metabolism and clearance and, consequently, the efficacy of therapy. In pathological conditions (such as viral infection, alcohol abuse, HCC, etc.), the expression and activity of these genes are modified.

The solute carriers (SLCs) are important cellular carriers, having consistent roles in different metabolic processes and tumorigenesis, and may become cellular targets for new therapeutic agents [36,37]. The analysis of SLC DEGs identified a series of dysregulated genes in the three tumor groups.

Our results are in accordance with data reported in the literature concerning the upregulation/overexpression of SLC genes in HCC, including *SLC44A5* and *SLC26A6* [38,40], and the downregulated expression of *SLC38A4* and *SLC22A1* [41,43]. A recent study evidenced *SLC26A6* as a novel oncogene in HCC [147].

Cancer testis (CT) genes are restrictedly expressed in normal tissues except for the testis and are aberrantly expressed in tumor tissues and often trigger humoral or cellular antitumor responses in patients with cancer. Expressed testis-derived antigens might be immunogenic because they have never been encountered by the immune system. Consequently, cancer testis (CT) genes have been indicated as a group of potential targets for TAA-specific immunotherapy [148].

A series of studies have demonstrated that CT antigens are regulators of cancer hallmarks, e.g., sustaining proliferative signaling, resisting cell death, and evading growth suppressors [149].

The MAGE and GAGE families are groups of tumor-specific antigens that can be used as molecular markers for early diagnosis but are also appropriate targets for vaccine-based cancer immunotherapy in human HCC [56,57].

Recent studies have revealed the involvement of members of the MAGE family in stress response pathways. CT MAGE-B2 is normally expressed in the testis but is highly expressed in tumors. It was reported that MAGE-B2 enhances cellular stress threshold by suppressing stress granule (SG) assembly and promoting cellular stress tolerance. This allows cancer cells to continue to grow even in stressful conditions [150].

Our analysis identified high expression of *MAGEB2*, *MAGEC3*, and *MAGEB17* only in the non-viral-infected tumor group, in which we observed the overexpression of genes involved in stress response. The overexpression of other CT genes, including *MAGEA1*, *PAGE4*, *PAGE5*, *GAGE2A*, *BAGE*, *BAGE3*, *BAGE4*, *BAGE5*, *ACTL8*, *TPTE*, *HSPB9*, *ATAD* and *DSCR8*, was shown in the three groups. Our results for the CT gene expression analysis are in accordance with data reported in the literature.

The next step in the analysis releveled common pathways and GO terms upregulated in the HCV and HBV groups, such as cell-cycle, mitotic, and PLK1 pathways. These results support the idea that hepatitis viruses deregulate the cell cycle in infected cells to promote an environment that can sustain viral replication. The persistence of viral infection and ineffective T cell responses maintain an inflammatory state in the liver that probably culminates in the onset of fibrosis, cirrhosis, and HCC.

In the non-viral-infected group, functional enrichment analysis revealed a significant upregulation of cellular stress response.

In HCV-related tumors, the downregulated genes were mainly enriched in “carcinogenesis-DNA adducts KEEG pathway” but also in “monocarboxylic acid metabolism” and “steroid metabolic processes” (demonstrating a reprogramed metabolism).

Considering that the liver is the site of major metabolic processes, it is not surprising that we identified altered metabolic pathways in our samples, as these may be highjacked by tumors in order to help them survive and proliferate, especially against a background of viral infection. It has been shown that HCV infection may have a direct effect on lipid metabolism and that cholesterol metabolism is decreased in HCV-infected HCC through the downregulation of genes involved in cholesterol synthesis, absorption and transport, and bile acid synthesis [151].

Immunome analysis revealed the immune expression patterns of the three patient groups that were analyzed. For example, the HCV tumors showed significant upregulation of T cell genes, as well as of genes with cytotoxic properties, markers of CD8 T cells, and cytotoxic cells, compared with tumors without viral infection. The HBV tumors showed a similar trend. High expression of such immune markers was previously reported to be associated with prolonged survival in many cancer types, such as colorectal cancer [27,152,153,154]. A similar expression pattern was observed for cytokines that modulate intratumoral immune infiltrate and also for immune checkpoint CTLA4 and PD-1 (*PDCD1*).

*CD96*, among T cell genes, is considered a novel immune checkpoint receptor target [155], and higher expression was associated with a poorer clinical outcome [156], while low *LCK* expression has been identified as a potential prognostic biomarker for immunotherapy in HCC [157]; however, the study did not segregate patients based on viral infection. In our study, we found *LCK* to be over-expressed in the HCV group but to have a lower expression in the non-B, non-C (non-viral) group.

We would like to acknowledge one limitation of the study, namely, the small number of patients. However, we wanted to give a close representation of real-world data by including the most common viral infections that represent risk factors for HCC and further compare these with data from non-viral-infected patients.

Further validation with bigger cohorts is needed, but we were able to determine both differences as well as commonalities between the three different HCC groups analyzed in our study. The HBV group was characterized by the smallest number of genes. Metascape analyses showed the upregulation of the cell-cycle pathway, similar to the HCV group. Additionally, the HCV group showed overexpression of a series of immune cells (including CTLA-4 and PDCD1) and downregulation of Cyp and SLC. The third group of patients (non-B, non-C/non-viral) showed the presence of upregulated Heat Shock Factor 1 and a series of HSPs as hub genes (upregulation of response to stress pathway), possibly pointing to HSF1/HSPs as modulators of apoptosis/autophagy in this group.

## 5. Conclusions

Our comparative RNA-seq analysis of liver cancer tumors revealed heterogeneity among HBV, HBV, and non-B, non-C (non-viral) tumors with regard to transcriptomic and immune profiles and contributes to a better understanding of the pathogenesis and progression mechanism of HCC.

## Figures and Tables

**Table 1:** Estimated age-standardized incidence rates (world) in 2020, liver, both sexes, all ages (Romania).

Parameter	Number
Number of Incident Cases	3615
Crude rate	18.8
ASR (world) per 100,000	8.8
Cumulative risk (0–74)	2.1

**Table 2:** Differentially expressed genes (DEGs) in the analyzed groups.

Group/Groups	Upregulated	Downregulated
Total HBV	120	102
Total HCV	465	226
Total non-B, non-C	441	187
Unique HBV	39	50
Unique HCV	331	145
Unique non-B, non-C	332	121

**Table 3:** Common up- and downregulated genes in all 3 tumor groups (HBV, HCV-related, and non-viral-infected tumors). Green background shows up-regulated genes; red background shows down-regulated genes.

Crt. No.	Differentially Expressed Genes (DEGs)	Tumor Group Etiology		
		HBV Log 2 (Ratio)	HCV Log 2 (Ratio)	Non-Viral (Non-B, Non-C) Log 2 (Ratio)
1	*LINC00383*—Long Intergenic Non-Protein Coding RNA 383	6.08	7.56	7.19
2	*LVCAT8*—Liver cancer-associated transcript 8	5.91	3.82	3.87
3	*ACTN2*—Actinin Alpha 2	5.48	2.73	4.26
4	*RAB9BP1 RAB9B*—Member RAS Oncogene Family Pseudogene 1	4.08	7.82	5.64
5	*MYO18B*—Myosin XVIIIB	3.88	6.74	5.49
6	*UBE2C*—Ubiquitin Conjugating Enzyme E2 C	3.79	5.13	3.7
7	*LVCAT5*—Liver cancer-associated transcript 5	3.54	8.85	6.62
8	*TROAP*—Trophinin Associated Protein	3.46	5.07	4.1
9	*CYP17A1*—Cytochrome P450 17A1	3.35	5.87	4.41
10	*TOP2A*—DNA Topoisomerase 2-Alpha	3.35	4.72	2.59
11	*DYNC1I1*—Dynein Cytoplasmic 1 Intermediate Chain 1	3.25	2.85	3.72
12	*LINC01344*—Long Intergenic Non-Protein Coding RNA 1344 *ZNF648*—Zinc Finger Protein 648	3.21	2.4	2.92
13	*CNR1*—cannabinoid receptor 1	3.19	2.77	3.95
14	*MELK*—Maternal Embryonic Leucine Zipper Kinase	3.01	5.07	3.16
15	*B4GALNT1*—Beta-1,4-N-Acetyl-Galactosaminyltransferase 1	2.84	4.95	3.94
16	*HIST1H2AI*—*Histone H2A type 1* *HIST1H3H*—Histone H3.1/Histone cluster 1, H3h	2.83	3.86	3.92
17	*ASPM*—Assembly Factor For Spindle Microtubules	2.81	4.92	2.92
18	*HIST1H3A*—*H3 Clustered Histone 1* *HIST1H3C*—H3 Clustered Histone 3	2.71	3.83	3.33
20	*FOXM1* Forkhead Box M1	2.66	3.69	3.66
21	*CAP2*—Cyclase Associated Actin Cytoskeleton Regulatory Protein 2	2.46	2.62	2.8
22	*TEX41*—Testis Expressed 41	2.13	4.7	4.13
23	*ATAD2*—ATPase Family AAA Domain Containing 2	1.89	2.19	1.58
24	*LINC0115*—Long Intergenic Non-Protein Coding RNA 1151 *LOC105375734*—Uncharacterized LOC105375734	1.86	3.56	3.63
25	*FASN*—Fatty acid synthase	1.74	2.25	3.73
26	*LAMA3*—Laminin Subunit Alpha 3	1.74	1.85	2.98
27	*CCDC162P*—*Coiled-coil domain containing 162, pseudogene* *LOC100996634*—Uncharacterized	1.49	1.77	−1.81
28	*CDKN2B-AS1*—CDKN2B Antisense RNA 1	1.43	2.21	1.48
29	*NR2F2-AS1*— Nuclear Receptor Subfamily 2 Group F Member 2 Antisense RNA 1	−1.41	−2.09	−1.51
30	*PCDHA1*—Protocadherin alpha-1;10;11;12;13… *PCDHA10; PCDHA11; PCDHA12; PCDHA13; PCDHA2; PCDHA3; PCDHA4; PCDHA5; PCDHA6; PCDHA7; PCDHA8; PCDHA9; PCDHAC1; PCDHAC2*	−1.54	1.57	2.07
31	*HGF*—Hepatocyte Growth Factor	−1.69	−2.22	−1.96
32	*IL33*—Interleukin 33	−1.79	−2.09	−2.44
33	*ADRA1A*—Adrenoceptor Alpha 1A	−1.81	−2.16	−1.55
34	*EDNRB*—Endothelin Receptor Type B	−1.96	−1.49	−1.79
35	*EGR1—Early Growth Response 1*	−1.98	−2.38	−1.98
36	*AFM*—Afamin *LOC728040*—Uncharacterized LOC728040	−2.11	−2.58	−2.11
37	*DCN*—Decorin	−2.22	−2.67	−3.05
38	*CYP39A1*—Cytochrome 39A1	−2.26	−2.28	−2.12
39	*ADAMTS9-AS2*— ADAM Metallopeptidase With Thrombospondin Type 1 Motif 9Antisense RNA2	−2.27	−2.75	−2.23
40	*C8orf4*—Thyroid Cancer Protein 1	−2.49	−2.76	−1.94
41	*C7*—Complement *C7*	−2.71	−3.84	−2.82
42	*LIFR*—LIF Receptor Subunit Alpha	−2.72	−2.78	−2.77
43	*LOC100506869*—Uncharacterized LOC100506869 *LOC101927653*—Uncharacterized LOC101927653	−2.96	−2.9	−3.11
44	*GHR*—Growth Hormone Receptor	−2.98	−2.7	−2.09
45	*COLEC10*—Collectin Subfamily Member 10	−3.52	−3.33	−3.05
46	*CLEC4M*—C-Type Lectin Domain Family 4 Member M	−5.56	−4.33	−3.29
47	*CYP2C8*—Cytochrome 2C8	−2.8	−4.89	−2.68

**Table 4:** Differentially expressed cytochromes.

Crt. No.	Differentially Expressed Genes (DEGs)	Tumor Group Etiology		
		HBV Log 2 (Ratio) Fold Change	HCV Log 2 (Ratio) Fold Change	Non-Viral (Non-B, Non-C) Log 2 (Ratio) Fold Change
1	CYP17A1	3.35	5.87	4.41
2	CYP7A1	3.03	3.12	
3	CYP39A1	−2.26	−2.28	−2.12
4	CYP2C8	−2.4	−4.89	−2.68
5	CYP2C9		−2.59; 8.14	
6	CYP2B6		−3.01	
7	CYP2B7P		−3.01	
8	CYP3A43		−2.94	−1.36
9	CYP2C18		−2.59	
10	CYP2C19		−2.59	
11	CYP3A4		−2.38	
12	CYP3A5		−2.38	
13	CYP3A7		−2.38	
14	CYP3A51P		−2.38	
15	CYP2E1		−1.95	−1.77
16	CYP4V2			−1.67
17	CYP1A2		−3.98	

**Table 5:** Differentially expressed human solute carriers (SLCs) and ABC transporters.

DEG HBV	Log 2 (Ratio) Fold Change	DEG HCV	Log 2 (Ratio) Fold Change	DEG Non-Viral	Log 2 (Ratio) Fold Change
SLC5A6	2.47				
		SLC5A1	−4.75		
		SLC22A1	−5.05		
				SLC25A36	−2.14
				SLC14A1	−3.46
				SLC8A1	−2.01
				SLC7A2	−1.98
				SLC4A2	2.14
		SLC9A3	1.76		
		SLC22A1	−3.12		
		SLC29A4	1.76		
				SLC6A9	3.26
		SLC26A6	2.14	SLC26A6	3.02
		SLC7A9	−2.47		
		SLC38A4	−2.05	SLC38A4	−1.61
		SLC44A5	2.77		
		SLC27A2	−1.47		
		SLC38A2	−1.41		
				SLC25A39	2.21
				SLC22A18	3.32
				SLC52A2	3.44
				SLC6A2	6.91
				SLC22A12	12.56
				ABCF1	1.95
				ABCB8	2.74
		SLC28A2	3.8		
		SLC2A5	3.81		
		SLC01C1	4.38		
		ABCB 5	5.57		
		ABCA 5	−1.19		
		ABCA 6	−1.19		
		ABCA 8	−1.19		
		ABCA 9	−1.19		
		ABCA10	−1.19		

**Table 6:** Differentially expressed CT genes.

Cancer Testis Antigenes and Related Genes			
Genes CTDatabase/Code	HBV	HCV	Non-B, Non-C
ATAD (ATPase Family AAA Domain)/CT 137	1.89	2.19	1.58
BUB1B (BUB1 Mitotic Checkpoint Serine/Threonine Kinase B)		4.52	
PBK (PDZ Binding Kinase)/CT 84		5.12	
MAGEA1 (MAGE Family Member A1)/CT 1.1	4.74		
MAGEC3 (MAGE Family Member C3)/CT 7.2			4.13
MAGEB17 (MAGE Family Member B17)			5.99
MAGEB2 (MAGE Family Member B2)/CT 3.2			9.05
PAGE 4 (Prostate-associated *gene 4*)/CT 16.4	9.58		
PAGE 5 (Prostate-associated *gene 5*)/CT 16.1	4.21		
GAGE2A/CT 4.1			11.86
BAGE (B Melanoma Antigen)/CT 2.1 BAGE 3 (B Melanoma Antigen 3)/CT 2.3 BAGE4 (B Melanoma Antigen 4)/CT 2.4 BAGE5 (B Melanoma Antigen 5)/CT 2.5	5.46	6.93	5.82
HSPB9 (Heat shock protein B family member 9/CT 51	4.7		
TPTE Transmembrane Phosphatase With Tensin Homology)/CT 44		6.93	5.82
ACTL8 (Actin like 8)/CT 57		11.23	9.12
FAM133A (Family With Sequence Similarity 133 Member A/CT 115			5.22
TEX41 (Testis Expressed 41)	2.13	4.7	4.13
TTK (MPS1—Serine/threonine-protein kinase/CT 96		4.71	

**Table 7:** Differentially expressed heat shock proteins and factors.

No.	Differentially Expressed Genes	Tumor Group Etiology		
		HBV Log 2 (Ratio) Fold Change	HCV Log 2 (Ratio) Fold Change	Non-Viral (Non-B, Non-C) Log 2 (Ratio) Fold Change
1	HSP90AA1 Heat Shock Protein 90 Alpha Family Class A Member 1			1.44
2	HSP90AB1, Heat Shock Protein 90 Alpha Family Class B Member 1			1.75
3	HSPA1A, Heat Shock Protein Family A (HSP70) Member 1A			2.33
4	HSPA5, Heat Shock Protein Family A (HSP70) Member 5			1.51
5	HSPB1 Heat Shock Protein Family B (HSP27) Member 1			5.65
6	HSPA1B Heat Shock Protein Family A (HSP70) Member B			2.33
7	HSPB9 Heat Shock Protein Family B (small HSP) Member 9	3.70		
8	HSF1 Heat Shock Factor 1			3.67
9	HSF4 Heat Shock Factor 4		1.84	
10	CDC37 Cell Division Cycle 37, HSP90 Cochaperone			1.54
11	BAG3 BAG family molecular chaperone regulator 3			1.98

**Table 8:** HUB genes in the non-viral group. Connectivity degrees were computed using GeneWalk.

Gene Symbol	Gene Description	Connectivity Degree (Ncon_Gene)
ACTB	Actin Beta	116
ACTG1	Actin Gamma 1	74
AGO2	Argonaute RISC Catalytic Component 2	68
ALDOA	Aldolase, Fructose-Bisphosphate A	54
ARF1	ADP Ribosylation Factor 1	51
ATP1A1	ATPase Na+/K+ Transporting Subunit Alpha 1	52
AURKB	Aurora Kinase B	59
BAG3	BAG Cochaperone 3	54
BCAR1	BCAR1 Scaffold Protein, Cas Family Member	57
CDC37	Cell Division Cycle 37, HSP90 Cochaperone	57
CTTN	Cortactin	74
CXCL8	C-X-C Motif Chemokine Ligand 8	78
EEF2	Eukaryotic Translation Elongation Factor 2	57
EZH2	Enhancer Of Zeste 2 Polycomb Repressive Complex 2 Subunit	57
GAPDH	Glyceraldehyde-3-Phosphate Dehydrogenase	95
GNB2	G Protein Subunit Beta 2	58
HNRNPA1	Heterogeneous Nuclear Ribonucleoprotein A1	82
HSF1	Heat Shock Transcription Factor 1	81
HSP90AA1	Heat Shock Protein 90 Alpha Family Class A Member 1	131
HSP90AB1	Heat Shock Protein 90 Alpha Family Class B Member 1	115
HSPA1A	Heat Shock Protein Family A (Hsp70) Member 1A	143
HSPA5	Heat Shock Protein Family A (Hsp70) Member 5	96
HSPB1	Heat Shock Protein Family B (Small) Member 1	56
LMNA	Lamin A/C	51
MCM2	Minichromosome Maintenance Complex Component 2	78
MYH9	Myosin Heavy Chain 9	86
PTK2	Protein Tyrosine Kinase 2	126
RARA	Retinoic Acid Receptor Alpha	95
RECQL4	RecQ Like Helicase 4	85
RUVBL2	RuvB Like AAA ATPase 2	62
SEC13	Homolog, Nuclear Pore And COPII Coat Complex Component	76
SLC8A1	Solute Carrier Family 8 Member A1	52
SMARCA4	SWI/SNF-Related, Matrix Associated, Actin DependentRegulator Of Chromatin, Subfamily A, Member 4	56
SQSTM1	Sequestosome 1	101
TUBB	Tubulin Beta Class I	60
U2AF2	U2 Small Nuclear RNA Auxiliary Factor 2	61

**Table 9:** HUB genes in the HCV group. Connectivity degrees were computed using GeneWalk.

Gene Symbol	Gene Description	Connectivity Degree (Ncon_Gene)
ACTN2	Actinin Alpha 2	60
ANXA2	Annexin A2	71
AURKA	Aurora Kinase A	63
AURKB	Aurora Kinase B	71
BRCA1	BRCA1 DNA Repair Associated	121
CCNB1	Cyclin B1	63
CDK1	Cyclin Dependent Kinase 1	99
CFTR	CF Transmembrane Conductance Regulator	78
CHEK1	Checkpoint Kinase 1	56
CYP1A2	Cytochrome P450 Family1 Subfam A Member 2	52
CYP3A4	Cytochrome P450 Family 3 Subfamily A Member 4	60
EGF	Epidermal Growth Factor	68
ENO1	Enolase 1	56
EZH2	Enhancer Of Zeste 2 Polycomb Repressive Complex 2 Subunit	62
HMGA2	High Mobility Group AT-Hook 2	63
IGF1	Insulin Like Growth Factor 1	104
MCM2	Minichromosome Maintenance Complex Component 2	69
NME1	Nucleoside Diphosphate Kinase 1	52
PCK1	Phosphoenolpyruvate Carboxykinase 1	52
PKM	Pyruvate Kinase M1/2	57
PRKDC	Protein Kinase, DNA-Activated, Catalytic Subunit	72
RECQL4	RecQ Like Helicase 4	68
SLC27A2	Solute Carrier Family 27 Member 2	52
SLC5A1	Solute Carrier Family 5 Member 1	51
SQSTM1	Sequestosome 1	72
TERT	Telomerase Reverse Transcriptase	58
TOP2A	DNA Topoisomerase II Alpha	52

## Data Availability

Data is contained within the article or supplementary material (as Supplementary Tables S3–S10).

## Supplementary Materials

The following supporting information can be downloaded at: https://www.mdpi.com/article/10.3390/medicina58121803/s1, Figure S1: Histology (hematoxylin-eosin staining) for the HCC patients (non-viral, HBV and HCV etiology), Figure S2: Tuxedo pipeline, Figure S3: HCV HUB and moonlighting genes enrichment, Figure S4: nonBnonC HUB and moonlighting genes enrichment, Figure S5: Immunome analysis in tumor tissues versus non-tumoral tissues and in TCGA cohort; Table S1: Patients’ features, Table S2: Primer sequences, Table S3: Data Common HBV & HCV, Table S4: Data Common HBV& nonBnonC, Table S5: Data Common HCV& nonBnonC, Table S6: List of up and down-regulated genes in HBV related HCC, Table S7: List of up and down-regulated genes in HCV related HCC, Table S8: List of up and down-regulated genes in non-viral HCC, Table S9: List of regulatory genes in HCV related group, Table S10: List of regulatory genes in non-viral group, Table S11: Clustering of immune gene data by ClueGO.
