# Supplementary material for: Comparative RNA-Sequencing Analysis Reveals High Complexity and Heterogeneity of Transcriptomic and Immune Profiles in Hepatocellular Carcinoma Tumors of Viral (HBV, HCV) and Non-Viral Etiology
Source: Medicina (Kaunas). 2022 Dec 7;58(12):1803. doi: 10.3390/medicina58121803 (PMC9785216; doi:10.3390/medicina58121803)
Supplement: Supplementary file 1 [file medicina-58-01803-s001.zip › medicina-2035589-supplementary.pdf]

Comparative RNA-sequencing analysis reveals high complexity and heterogeneity of transcriptomic and immune profiles in hepatocellular carcinoma tumors of viral (HBV, HCV) and non-viral etiology

Supplementary data

Contents

Supplementary Table 1. Patients` features 2

Supplementary Table 2. Primer sequences 3

Supplementary Table 3. Data Common HBV & HCV 4

Supplementary Table 4. Data Common HBV& nonBnonC 5

Supplementary Table 5. Data Common HCV& nonBnonC 6

Supplementary Table 6. List of up and down-regulated genes in HBV related HCC 8

Supplementary Table 7. List of up and down-regulated genes in HCV related HCC 10

Supplementary Table 8. List of up and down-regulated genes in non-viral HCC 19

Supplementary Table 9. List of regulatory genes in HCV related group 29

Supplementary Table 10. List of regulatory genes in non-viral group 30

Supplementary Table 11. Clustering of immune gene data by ClueGO 31

Supplementary Figure 1. Histology (hematoxylin-eosin staining) for the HCC patients (non-viral, HBV and HCV etiology) 33

Supplementary Figure 2. Tuxedo pipeline 34

Supplementary Figure 3. HCV HUB and moonlighting genes enrichment 35

Supplementary Figure 4. nonBnonC HUB and moonlighting genes enrichment 44

Supplementary Figure 5. Immunome analysis in tumor tissues versus non-tumoral tissues and in TCGA cohort 59

Supplementary Table 1. Patients` features

Variables	HBV group (n=8)	HCV group (n=8)	Non infection group (n=8)	*P* -Value
Age (mean ± SD)	57 ± 13	64 ± 6	62 ± 9	0.4251
Gender (n, %)				
Male % (n)	62.5% (5)	37.5% (3)	75% (6)	0.3012
Female % (n)	37.5% (3)	62.5% (5)	25% (2)	
Tumor size cm (mean ± SD)	3.1 ± 1.6	4.4 ± 2.4	5.9 ± 3.58	0.1257
Macrovascular invasion				
Yes	12.5 % (1)	25 % (2)	12.5 % (1)	0.7408
No	87.5 % (7)	75 % (6)	87.5 % (7)	
Microvascular invasion				
Yes	0%	25 % (2)	12.5 % (1)	0.3189
No	100% (8)	75 % (6)	87.5 % (7)	
Stages				
I % (n)	50 % (4)	50 % (4)	75 % (6)	0.3964
II % (n)	37.5 % (3)	37.5 % (3)	0% (0)	
III % (n)	12.5 % (1)	12.5 % (1)	25% (2)	
Overall Survival (months) (mean ± SD)	37.63 ± (13.87)	28.13 ± (19.34)	30.88 ± (13.89)	0.5846
PIVKA mAU/ml (mean ± SD)	925.4 ± 1419	6706 ± 8295	11980 ±13042	0.0969
Serum AFP (ng/ml) (mean ± SD)	198.6 ±412.5	833.3 ± 812.1	134.5 ± 349.9	***0.0451***

The *p* values are from the One-way ANOVA /Chi-square (and Fisher's exact) test; SD, standard deviation.

Supplementary Table 2. Primer sequences

Oligo name	Sequence
COLEC10_F	ATA-GCC-GTC-CTA-CCG-CTG-AA
COLEC10_R	TGA-TCT-CCC-ATA-TCA-CCC-AGT-TC
HGF_F	GCT-ATC-GGG-GTA-AAG-ACC-TAC-A
HGF_R	CGT-AGC-GTA-CCT-CTG-GAT-TGC
CLEC1B_F	GAC-AAC-CGG-AAC-ATT-GTG-GAG
CLEC1B_R	ACT-TCT-GGC-GAG-ATA-ATC-CGA
CYP17A1_F	TAT-GGC-CCC-ATC-TAT-TCG-GTT
CYP17A1_R	GCG-ATA-CCC-TTA-CGG-TTG-TTG
HSF1_F	CCA-TGA-AGC-ATG-AGA-ATG-AGG-C
HSF1_R	CTT-GTT-GAC-GAC-TTT-CTG-TTG-C
BIRC5_F	AGG-ACC-ACC-GCA-TCT-CTA-CAT
BIRC5_R	AAG-TCT-GGC-TCG-TTC-TCA-GTG
HSP90AB1_F	AGA-AAT-TGC-CCA-ACT-CAT-GTC-C
HSP90AB1_R	ATC-AAC-TCC-CGA-AGG-AAA-ATC-TC
SLC22A1_F	TGT-CAC-CGA-AAA-GCT-GAG-CC
SLC22A1_R	TCC-GTG-AAC-CAC-AGG-TAC-ATC
HSPB1_F	ACG-GTC-AAG-ACC-AAG-GAT-GG
HSPB1_R	AGC-GTG-TAT-TTC-CGC-GTG-A
RNF187_F	GTG-ATG-GAC-CGT-AGG-AAG-AAG-G
RNF187_R	GTG-ACC-TGA-ACC-GCT-CAG-TG
FGFR4_F	GAG-GGG-CCG-CCT-AGA-GAT-T
FGFR4_R	CAG-GAC-GAT-CAT-GGA-GCC-T

Supplementary Table 3. Data Common HBV & HCV

Crt. No.	Differentially expressed genes (DEGs)	Tumor group etiology		
		HBV log 2 (ratio)	HCV log 2 (ratio)	Non viral (non-B, non-C) log 2 (ratio)
**Up-regulated genes**				
1	MTNR1B	6.2	7.42	0
2	AFP	5.93	5.02	0
3	COL7A1;	5.43	7.24	0
4	RGSL1	4.85	3.72	0
5	PDE7B	4.83	5.75	0
6	FMO2	4.25	2.52	0
7	MDK	3.81	3.27	0
8	EGF	3.8	6.23	0
9	HAGLR	3.76	4.26	0
10	LDB2	3.72	4.57	0
11	EPS8L3	3.63	4.31	0
12	ANLN	3.53	5.62	0
13	KIF18B	3.48	5.65	0
14	CDC25C	3.4	4.85	0
15	CENPF	3.37	5.08	0
16	CPNE7	3.32	3.14	0
17	DEPDC1	3.31	5.64	0
18	EXO1	3.18	4.8	0
19	CYP7A1	3.03	3.12	0
20	MKI67	3.01	4.59	0
21	CDK1	2.98	4.35	0
22	CENPE	2.92	3.77	0
23	HMMR	2.91	3.92	0
24	C2orf48; RRM2	2.84	4.06	0
25	CCNB1	2.71	3.99	0
26	KCCAT333;	2.69	1.92	0
27	PRC1	2.64	3.61	0
28	TTC39A	2.63	3.37	0
29	SPP1	2.3	3.69	0
30	ARFGEF3	2.24	2.61	0
31	GREB1	2.21	1.84	0
32	NUSAP1	2.21	3.07	0
33	TPX2	2.15	3.6	0
34	SNAP25-AS1	1.93	1.88	0
35	GDNF-AS1	1.79	2.43	0
36	DPY19L2P2	1.78	1.58	0
**Down-regulated genes**				
1	MASP1	-1.66	-1.67	0
2	SERPINA11	-1.69	-2.1	0
3	C8B	-1.73	-2.27	0
4	NRG1	-1.76	-1.89	0

5	RCAN1	-1.83	-1.7	0
6	FOS	-1.86	-2.25	0
7	RND3	-1.9	-1.97	0
8	WDR17	-2	-1.78	0
9	NPR3	-2.08	-2.05	0
10	OLFML3	-2.26	-1.87	0
11	SEMA6D	-2.31	-2.57	0
12	TNXB	-2.31	-1.96	0
13	COL25A1	-2.36	-3.37	0
14	IGFBP3	-2.51	-1.99	0
15	STEAP4	-2.56	-2.72	0
16	CXCL12	-2.76	-2.97	0
17	PHACTR3	-2.76	-3.9	0
18	ANKRD18DP	-3.07	-3.23	0
19	DNASE1L3	-3.13	-2.87	0
20	HMGCLL1	-3.27	-4.07	0
21	CD5L	-3.41	-2.16	0
22	NDST3	-3.96	-4	0
23	NOL4	-4.35	-3.21	0

Supplementary Table 4. Data Common HBV& nonBnonC

Crt. No.	Differentially expressed genes (DEGs)	Tumor group etiology		
		HBV log 2 (ratio)	HCV log 2 (ratio)	Non viral (non-B, non-C) log 2 (ratio)
**Up-regulated genes**				
1	ODAM	6.14	0	9.05
2	CPLX2	5.32	0	10.41
3	SULT4A1	5.21	0	4.19
4	PAPPA2	5.02	0	3.13
5	FAM133A	4.7	0	5.22
6	MEOX2; MEOX2-AS1	4.17	0	3.55
7	NBPF22P	3.94	0	5.19
8	THBS4	3.91	0	2.58
9	GPC3	3.58	0	5.24
11	SMPX	3.14	0	4.03
12	NKD1	2.9	0	3.33
13	BRDTP1	2.3	0	3.19
14	RDM1	1.99	0	2.76
**Down-regulated genes**				
1	IL1RAPL2	-1.52	0	-2.18
2	VSIG4	-1.68	0	-2.18
3	MRC1	-1.76	0	-2.44

4	CYP2C8	-2.4	0	-2.68
5	SPP2	-2.51	0	-2.75
6	KCNJ16	-3.14	0	-3.2
7	MXD4	-3.47	0	-3.83
8	TDRD1	-4.33	0	-4.62
9	LRRC75A-AS1; SNORD49B	-4.62	0	4.58

Supplementary Table 5. Data Common HCV& nonBnonC

Crt. No.	Differentially expressed genes (DEGs)	Tumor group etiology		
		HBV log 2 (ratio)	HCV log 2 (ratio)	Non viral (non-B, non-C) log 2 (ratio)
**Up-regulated genes**				
1	GRINA	0	1.22	3.48
2	GLI4; ZFP41	0	1.35	3.74
3	TUBB	0	1.38	1.26
4	BSG	0	1.39	3.15
5	RNF187	0	1.45	2.35
6	SNRPB	0	1.46	2.04
7	PABPC1	0	1.48	1.93
8	HGS	0	1.52	2.13
9	MROH1	0	1.56	2.41
10	GLMP; TMEM79	0	1.68	1.98
11	PVT1	0	1.74	2.08
12	PRKDC	0	1.77	1.63
13	TMEM150B	0	1.79	3.31
14	ALDOA	0	1.83	2.97
15	MSTO1; STO2P	0	1.89	2.18
16	SCRIB	0	1.96	3.92
17	SQSTM1	0	1.99	2.24
18	TKT	0	2.07	3.3
19	SLC26A6	0	2.14	3.02
20	EZH2	0	2.22	2.57
21	NDRG1	0	2.3	2.08
22	ASPH	0	2.38	2.87
23	TXNRD1	0	2.38	2.44
24	COL5A3	0	2.4	3.27
25	CDRT1; TRIM16	0	2.45	3.99
26	HIST1H2AD; HIST1H3D	0	2.46	2.91
27	HMGA1	0	2.46	3.29
28	SRXN1	0	2.5	2.69
29	FBXW10; RIM16L	0	2.74	3.17
30	MCM4	0	2.91	2.3
31	LUCAT1	0	3.09	4.16

32	CABYR	0	3.12	4.94
33	ROBO1	0	3.2	2.06
34	HIST1H2AL; IST1H2BM; HIST1H2BN; HIST1H2BO; OR2B6		3.31	3.64
35	MCM2	0	3.47	2.7
36	RAD54L	0	3.61	3.29
37	RECQL4	0	3.65	4.71
38	SPINK5	0	4.47	3.2
39	HIST1H2AK; HIST1H2BL	0	3.78	4.89
40	TICRR	0	3.88	2.73
41	NQO1	0	3.93	4.14
42	DIAPH3	0	3.94	2.32
43	POLQ	0	4.0	2.35
44	PTPRQ	0	4.03	7.35
45	ZFPM2-AS1	0	4.03	3.48
46	HIST1H2AL	0	4.07	3.86
47	TLX1	0	4.07	3.09
48	FBXO43	0	4.22	2.9
49	SERINC2	0	4.37	5.48
50	CNTNAP4;	0	4.78	4.1
51	SNX29	0	4.87	5.28
52	AKR1B10; KR1B15	0	5.33	5.08
53	ALDH3A1	0	5.96	8.89
54	IGF2BP1	0	6.37	4.36
55	BAGE; BAGE3; BAGE4; BAGE5; TPTE	0	6.93	5.82
56	LSP1P3	0	7.33	5.94
57	RIMS2	0	7.46	5.29
58	SPINK4	0	10.18	13.45
59	ACTL8	0	11.23	9.12
**Down-regulated genes**				
1	PKHD1	0	-3.99	-2.76
2	LINC01093	0	-3.96	-3.19
3	GPM6A	0	-3.86	-3.22
4	ADGRB3	0	-3.85	-2.62
5	HHIP	0	-2.96	-2.57
6	CYP3A43	0	-2.94	-1.36
7	ATOH7	0	-2.79	-2.28
8	HBB; HBD	0	-2.79	-2.57
9	EPHA3	0	-2.73	-2.48
10	PDGFRA	0	-2.71	-3.16
11	C8A	0	-2.68	-1.44
12	ASIC5; TDO2	0	-2.66	-2.47
13	SOCS2	0	-2.5	-3.25
14	MMRN1	0	-2.48	-2.01
15	SLC7A2	0	-2.47	-1.99
16	JCHAIN	0	-2.45	-2.67

17	TDRD15	0	-1.99	-3.02
18	AMDHD1	0	-2.4	-1.73
19	HPX	0	-2.35	-1.8
20	NAT1; NAT2	0	-2.35	-2.41
21	MACC1	0	-2.33	-3.23
22	IL1RAP	0	-2.24	-1.89
23	PKHD1L1	0	-2.19	-2.19
24	SH3YL1	0	-2.19	-2.37
25	GLYATL1	0	-2.16	-2.06
26	SLC38A4	0	-2.05	-1.61
27	CYBRD1	0	-2.01	-1.77
28	DAB1	0	-1.99	-1.72
29	CYP2E1	0	-1.95	-1.77
30	APOF; STAT2	0	-1.91	-1.81
31	PLA2R1	0	-1.89	-1.75
32	A2M; PZP	0	-1.81	-1.73
33	ACAA2	0	-1.76	-1.64
34	SOCS2-AS1	0	-1.67	-1.63
35	PLG	0	-1.52	-1.75
36	PLSCR4	0	-1.52	-2.07
37	ID2	0	-1.47	-1.8

Supplementary Table 6. List of up and down-regulated genes in HBV related HCC

HBV unique upregulated in tumor	FC
SCGB1D2	13.44
DSCR8	11
PAGE4	9.58
ODAM;PRR27	9.41
HOXC9	8.61
SFTA1P	8.57
CSN1S2BP	8.39
MIR552	6.41
BAGE;BAGE3;BAGE4;BAGE5	5.46

HBV unique downregulated in tumor	FC
MEG8;MIR370;MIR411;MIR494;SNHG24;SNORD113-1;SNORD113-2;SNORD113-4;SNORD113-9;SNORD114-1;SNORD114-10;SNORD114-11;SNORD114-13;SNORD114-14;SNORD114-17;SNORD114-26;SNORD114-27;SNORD114-28;SNORD114-29;SNORD114-30;SNORD114-31;SNORD114-4;SNORD114-5;SNORD114-6;SNORD114-7;SNORD114-8	-5.25
CLEC1B	-5.11
FCN2	-4.9
LINCR-0001;PRSS55	-4.47
CRHBP	-4.14
MIR10A	-4.06
THSD7A	-3.79
DSCAM	-3.63
BBOX1	-3.54

PEG10	5.29
LOC101928775	5.24
LOC80078	4.98
MAGEA1	4.74
SPINK1	4.7
CTNNA2;LOC101927987	4.68
MIR4441	4.53
HOXA10;HOXA10-HOXA9;HOXA9	4.43
LOC100421746;PAGE5	4.21
MGAM2	3.74
ALG1L	3.45
LOC101928911;SPACA1	3.31
NOTUM	3.31
KIF4A	3.08
MIR325;MIR325HG	3.04
MIR4454	2.9
LGR5	2.81
IFI27	2.69
MGC32805;SNCAIP	2.58
FAM53A	2.5
SLC5A6	2.47
HOXA3	2.37
PTCHD4	2.28
EGFEM1P	2.28
MROH2A	2.25
ST8SIA3	2.21
LINC01010;LOC101928231;LOC101928304	2.19
LOC101927334	1.92
CDHR3	1.44
ATP6AP1L	1.38

HAO2;HSD3B2	-3.44
PLCXD3	-3.4
LRRC7	-3.28
GBA3;MIR548AJ2	-3.26
ADAMTS13	-3.16
ECM1	-3.12
CDH19	-3.09
FAM47E-STBD1	-3.06
KDM8	-2.81
DPT	-2.53
IL5RA	-2.45
NPY1R	-2.33
ALLC	-2.31
CCL19	-2.29
FGF14;FGF14-IT1	-2.2
KCNE1	-2.18
TDO2	-2.18
TPTE2	-2.16
XDH	-2.14
FCRL3	-2.13
KIAA1456	-2.12
HSD17B13	-2.11
FAM188B;INMT;INMT-FAM188B	-2.1
DTHD1	-2.09
MYO10	-2.02
CXCL2	-1.98
CPEB3	-1.95
IFNG-AS1;IL26	-1.92
CD163	-1.92
RASGEF1B	-1.92
FBP1	-1.87
EMBP1	-1.87
ANXA10	-1.82
PHLDA1	-1.79
LINC00548;LINC00598	-1.75
MT1L;MT2A	-1.71

MS4A6A	-1.69
LILRA6;LILRB2;LILRB3;LILRB5	-1.66
CCDC141;TTN	-1.63
CEP128;TSHR	-1.55
LOC101927156	-1.25

Supplementary Table 7. List of up and down-regulated genes in HCV related HCC

HCV unique upregulated in tumor	FC
REG3A	10.59
ANKRD33	10.38
LINC00162	9.68
TUBA3C	9.17
MIR5007	8.83
KHDC1L	8.48
ALX1	8.29
CYP2C9	8.14
FSTL5	8.05
LINC01142	7.54
CLVS2	7.46
CARD18	7.32
LINC00564	7.3
PRAME	7.3
CDH9	7.28
MIR631	7.25
OR8A1;OR8G1	7.22
LOC101929645	7.2
LINC01419	7.14
GALNT5	7.13
LINC00906;LOC102724958	7.01
NPSR1-AS1	6.95
LINC01611	6.91
LOC102723828	6.89
CD200R1	6.86
LOC101929694	6.84

HCV unique downregulated in tumor	FC
CNTN3	-5.8
GRIN2B	-5.73
FREM2	-5.63
MIR378H	-5.62
TRPV6	-5.53
FAM83F;LOC100130899	-5.36
MUC6	-5.32
CFTR	-5.26
SLC22A1	-3.12
MROH2B	-5.02
GABRB3	-5
ACSM6;CYP2C8	-4.89
GABRP	-4.89
SLC5A1	-4.75
CXCL14	-4.57
IGF2;INS;INS-IGF2	-4.5
GRHL2	-4.49
GLS2	-4.36
VIPR1	-4.12
ADGRG7	-4.09
PROM1	-4.09
LRRTM3	-4.06
CYP1A2	-3.98
CTNNA3	-3.97
PWRN1;PWRN2;PWRN3	-3.96
KCNN2	-3.93

LOC101927081	6.78
CNTNAP2	6.74
LOC102723968	6.63
LOC344887	6.56
CYTL1;LOC101928306	6.55
LOC101929584	6.43
TRIM71	6.36
MMP13	6.32
FEZF1-AS1	6.25
CSMD1	6.13
SKA1	6.13
GRM7-AS3	6.08
CDH7	6.05
ST8SIA6-AS1	5.96
CCDC144NL	5.89
ZIC4	5.88
MYBPC1	5.84
CASC20	5.76
ABCB5	5.57
C12orf56	5.56
NEK2	5.55
OSTN	5.54
BIRC5	5.52
TERT	5.5
DMP1	5.48
NPFFR2	5.45
PPP2R2C	5.44
XIRP2	5.41
LOC101927062	5.31
LINC00942	5.28
IBSP	5.26
LINCR-0002	5.2
CCDC169;CCDC169-SOHLH2;SOHLH2	5.16
KIF2C	5.12
PBK	5.12
HAVCR1	5.1

SYT9	-3.92
MFSD2A	-3.77
BMP10	-3.74
HAND2-AS1	-3.61
AJAP1	-3.55
ESRRG	-3.53
SHBG	-3.53
FENDRR	-3.51
AGBL4	-3.41
LOC100506869	-3.36
GNAO1	-3.3
OR5AK2;OR5AK4P	-3.3
BZRAP1;MIR142	-3.27
MIR1247	-3.26
SCN7A	-3.26
IDO2	-3.23
TIMD4	-3.21
CLSTN2	-3.12
FLJ22763	-3.12
MOGAT2	-3.05
NCAM1	-3.02
CYP2B6;CYP2B7P	-3.01
ASPG	-2.98
UROC1	-2.97
PCK1	-2.93
PGLYRP2	-2.92
BCHE	-2.91
FAM65C	-2.89
GSTA2	-2.82
ACSM3	-2.81
TNFRSF13B	-2.81
COL4A3	-2.73
FAM47E-STBD1;STBD1	-2.69
FAM129C	-2.62
LINC00924;LINC01197	-2.62
CYP2C18;CYP2C19;CYP2C9	-2.59

KIF20A	5.09
E2F7	5.08
CCNYL2	5.04
CNTN6	5.04
C21orf91	5.01
LINC00648	5.01
HMGA2	4.98
DLGAP5	4.97
NCAPG	4.94
GTSF1	4.9
CYP19A1	4.89
ROS1	4.87
CEP55	4.84
IGF2BP3	4.84
ERC2	4.82
TSPEAR	4.8
CDC45	4.76
TTK	4.71
UHRF1	4.68
GPR158	4.67
MATN3	4.66
SH3GL3	4.64
AXDND1	4.62
CDCA2	4.62
CKAP2L	4.62
SULT1C2	4.59
TRIP13	4.59
MCM10	4.57
XIRP1	4.57
ZNF444	4.57
CEACAM20	4.54
RGS13	4.54
WDR75	4.52
BUB1B;PAK6	4.52
LRFN5	4.46
SPTA1	4.45

LIFR-AS1	-2.57
GRAMD1C	-2.56
ANKRD55	-2.49
COL19A1	-2.49
NIPAL1	-2.49
UGT2B10;UGT2B28;UGT2B7	-2.49
LRRC4C	-2.48
HAL	-2.47
FCRL2	-2.45
CETP	-2.44
RDH16;ZBTB39	-2.43
RIC3	-2.42
GLYAT	-2.4
IGF1	2.61
TTR	-2.39
CYP3A4;CYP3A5;CYP3A7;CYP3A7-CYP3A51P	-2.38
HSD17B2	-2.28
FGB	-2.26
OR10J5	-2.25
IL1RL1	-2.24
C9;DAB2;FYB	-2.23
FGFR2	-2.22
NR4A1	-2.2
LOC200772	-2.19
FBLN5	-2.18
PLA2G5	-2.18
AZGP1	-2.17
FXYD1;FXYD7	-2.15
FAM110C	-2.13
PRELP	-2.12
AQP1;FAM188B;INMT;INMT-FAM188B	-2.09
ERRFI1	-2.03
RBMS3	-2.03
SPINT2	-1.96
TAT;ZNF19;ZNF23	-1.96
F11	-1.95

RPL10L	4.42
MIR4426	4.41
SLCO1C1	4.38
KIF14	4.36
TCN1	4.35
MKRN3	4.34
HIST1H3B	4.33
MUC15	4.33
CDKN3	4.32
PPP1R15B	4.3
GTSE1	4.29
GSDMC	4.28
BRSK2	4.23
CASC9	4.21
KIF23	4.13
RFX8	4.11
CACNA1E	4.1
LINC01060	4.1
MIR2052;MIR2052HG;PI15	4.1
MIR3144	4.1
POSTN	4.05
HHLA2	4.04
FAM111B	4.02
LINC00491	3.99
LINC01229	3.99
DUXAP8	3.98
CD200R1L	3.92
LINC01468	3.92
NRCAM	3.9
CALCR	3.86
NEIL3	3.81
SLC2A5	3.81
CDCA3	3.8
SLC28A2	3.8
ZFPM2	3.8
GPSM2	3.78

ALLC;COLEC11	-1.94
CPN1	-1.94
FCRL1	-1.94
GSTA1;GSTA3	-1.89
PTGIS	-1.86
SERPINF2	-1.86
GCH1	-1.85
FABP1	-1.83
LINC01197	-1.82
ADAMTS1	-1.81
ADAMTSL3	-1.81
ID1;MIR3193	-1.78
ITGA9	-1.77
MEF2C-AS1	-1.77
ADRA1B;LOC101927766	-1.76
COL14A1	-1.76
PANK1	-1.7
APOH	-1.69
OGDHL	-1.69
CYR61	-1.65
GADD45B	-1.64
LIPG	-1.63
SIK1	-1.63
SYNPO2	-1.63
C1R;C1RL	-1.57
AGXT	-1.55
MAT1A	-1.55
PEG3;ZIM2	-1.55
ACSL1	-1.53
ABAT	-1.51
BDH2	-1.51
DUSP1	-1.5
MPC1	-1.5
SLC27A2	-1.47
FGL1	-1.45
GABARAPL1	-1.41

TFAP2A	3.78
APCDD1L	3.76
EGFEM1P;LOC100507661	3.76
OSR2	3.76
EBF2	3.74
LOC613266	3.71
CRNDE	3.7
OR2AG1	3.7
PABPC4L	3.69
PYCR1	3.68
RIBC2	3.66
C5orf46	3.64
WDR62	3.61
ILDR2	3.57
HIST1H2AB	3.56
KIAA0101	3.55
PPP1R1B	3.53
GAD1	3.5
KIF11	3.49
PKIA-AS1	3.47
ZWINT	3.47
FXYD3	3.46
AURKA	3.41
LOC339862	3.41
MAEL	3.41
IL31RA	3.4
PRR19	3.4
RACGAP1	3.4
SMKR1;STRIP2	3.4
PRR11	3.39
TMEM74	3.39
TRPC3	3.39

SLC38A2	-1.41
DGAT2	-1.4
THBS1	-1.35
PPP1R3B	-1.34
IRF8	-1.33
MEG8;MIR370;SNHG24;SNORD113-1;SNORD113-2;SNORD113-4;SNORD113-5;SNORD113-6;SNORD114-1;SNORD114-10;SNORD114-14;SNORD114-17;SNORD114-20;SNORD114-21;SNORD114-23;SNORD114-28;SNORD114-30;SNORD114-31;SNORD114-4;SNORD114-5;SNORD114-6;SNORD114-7;SNORD114-8	-1.32
GOT1	-1.31
MUT	-1.3
MYOT	-1.28
ABCA10;ABCA5;ABCA6;ABCA8;ABCA9;PRO1804	-1.19
LINC00987;LOC101930452;LOC642846	-0.89

ADAM23	3.38
CENPM	3.36
LOC101929771	3.36
RMST	3.36
BRDT;EPHX4	3.35
HIST1H3J	3.34
OLFML2B	3.33
CD109	3.32
FOXN4	3.32
MEPE	3.25
ADAM12	3.24
UBE2U	3.23
LINC01301	3.21
E2F2	3.18
PARPBP	3.17
LRP1B	3.16
MSC	3.15
LOC101927592	3.14
HIST1H2BB	3.12
ECT2	3.1
NOX4	3.1
HIST1H3I	3.09
GDNF	3.08
LINGO1	3.08
MIR193A	3.08
PTHLH	3.06
TRPC7	3.06
SPHK1	3.05
SUSD4	3.05
HIST1H2AJ;HIST1H2AK	2.98
HIST1H2AG;HIST1H2AH;MIR3143	2.97
WNT5A	2.96
KPNA2	2.95
LINC01021	2.94
GJC1	2.93
DNAJC6	2.92

BBC3;MIR3191	2.91
RNF144A-AS1	2.9
WFDC21P	2.89
C6orf141	2.88
DUOX2	2.88
LOC101928858	2.87
STIL	2.87
CAPN9	2.86
PPFIA4	2.86
TCF19	2.86
LINC01594	2.84
CELSR3;MIR4793	2.83
DUXAP10;LINC01296	2.81
LOX	2.81
LOC440982;ZIC1	2.79
BCAT1	2.78
LINC01426	2.78
ZNF385D	2.78
SLC44A5	2.77
FCAMR	2.76
HELLS	2.74
HOXC4;HOXC5;HOXC6	2.74
C21orf58	2.69
TMEM232	2.69
BTNL8	2.66
LINC00470	2.62
CENPL	2.58
LOC441666	2.55
ARHGAP11B;LOC100288637	2.53
LOC654342	2.52
LOC101928622	2.51
C5orf45	2.5
FANCI	2.5
DNAH12	2.49
HIST1H2AE;HIST1H2BE;HIST1H2BF;HIST1H2BI	2.47
FAM78B	2.44

GPR35	2.44
ADAMTS14	2.43
CECR7	2.42
LMNB2	2.42
ACSM1	2.41
MGAM	2.4
PLCB1	2.4
ITGB3	2.38
UBD	2.38
DNAH8	2.37
LINC00535	2.33
FABP5	2.31
MAP2	2.31
GOLGA2P7;LOC642423	2.3
FIGNL2	2.28
INCENP	2.27
ADM	2.23
P4HA2	2.23
GAP43	2.22
LMNB1	2.22
TSPEAR;TSPEAR-AS1;TSPEAR-AS2	2.22
AURKB;LINC00324	2.21
BBS7;CCNA2	2.21
MTL5	2.21
TUBA1C	2.2
LOC100507195	2.14
ITGA5	2.12
HN1	2.11
BRIP1	2.1
HIST2H2BC;HIST2H2BE	2.09
MCM3	2.07
MCM6	2.06
SCARA3	2.06
SQLE;ZNF572	2.06
LINC01134	2.03
SMC4	2.02

CSTA	1.99
CDC25B;LOC101929125	1.97
KCNMB2;KCNMB2-AS1	1.97
PKM	1.94
CALCA	1.92
IL4I1;NUP62	1.91
SERPINH1	1.89
SPAG5	1.88
STIP1	1.87
CCT3	1.85
SLC29A4	1.85
FBXL8;HSF4	1.84
ENO1	1.83
MMP14;MRPL52	1.83
BRCA1	1.82
DTNA	1.81
EML6	1.79
GSDMD;ZC3H3	1.79
RRP12	1.78
SOGA1	1.78
LOC100288152;SLC9A3	1.76
ANXA2	1.75
ENAH	1.7
MIR3917;PAQR7;STMN1	1.68
VPS45	1.68
CKAP2	1.67
CKAP4	1.67
DLAT	1.67
GUCY1B2	1.65
LRRC37A8P;RDM1	1.65
H2AFZ	1.64
LINC01515	1.63
ADAMTS6	1.61
LAMC1	1.61
MCM8	1.61
MIR5195	1.61

TDRKH	1.61
NME1;NME1-NME2;NME2	1.59
HK1;HKDC1	1.56
XPOT	1.55
C1orf204;CFAP45;TAGLN2;VSIG8	1.53
LINC01572;PMFBP1	1.5
SCD	1.48
CHD1L	1.47
ACLY	1.46
ONECUT2	1.45
ITGA6	1.42
PSMD4	1.4
SAE1	1.39
TMEM64	1.38
TUBA1A;TUBA1B	1.38
CFL1	1.36
NPLOC4	1.33
LRRC59	1.28
MIR3610;RAD21	1.28
CHEK1;STT3A	1.19
SSRP1	1.14

Supplementary Table 8. List of up and down-regulated genes in non-viral HCC

nonBnonC unique upregulated in tumor	FC
SLC22A12	12.56
GAGE2A	11.86
RPS17	11.66
HOXB13	9.89

nonBnonC unique downregulated in tumor	FC
MEG8;MIR370;SNHG24;SNORD113-1;SNORD113-2;SNORD113-4;SNORD113-5;SNORD113-6;SNORD113-7;SNORD113-9;SNORD114-1;SNORD114-10;SNORD114-11;SNORD114-14;SNORD114-17;SNORD114-2;SNORD114-20;SNORD114-21;SNORD114-23;SNORD114-24;SNORD114-26;SNORD114-27;SNORD114-28;SNORD114-3;SNORD114-30;SNORD114-31;SNORD114-4;SNORD114-5;SNORD114-6;SNORD114-7	-5.74
AP1B1;RFPL1	-5.12
ESRP1	-4.41
VTCN1	-4.35

GIF	9.72
LINC01242	9.46
GIP	9.09
MAGEB2	9.05
CELA3A	8.97
CSN1S2AP;ODAM	8.59
LINC01139	7.5
SNORD93	7.31
SLC6A2	6.91
FLNC	6.36
TGM3	6.35
CCDC136	6.1
LINC01322	6.06
CDH17	6.02
GRPR;MAGEB17	5.99
GP2	5.76
FER1L6	5.68
LOC101927948;LOC101927967	5.68
ENPP7P13	5.66
HSPB1	5.65
DCC	5.6
LOC101929645;LOC101929660	5.42
LDLRAD1	5.17
RHBG	5.12
VCX	4.95
ISX	4.91
LOC653712	4.8
CCDC124	4.71
L1CAM	4.57
ORAOV1	4.53
DIO2	4.51
MYO1A	4.46
CAMK2A	4.45
SP5	4.42

STK32A	-4.26
ATP13A4	-3.59
PTPRZ1	-3.58
BDKRB1;BDKRB2	-3.48
SLC14A1	-3.46
FLG-AS1	-3.41
FLG;HRNR	-3.34
ERP27	-3.3
GALNT3	-3.11
IPW;PWAR1;PWAR5;PWARSN;SNORD109B;SNORD116-10;SNORD116-16;SNORD116-17;SNORD116-18;SNORD116-21;SNORD116-22;SNORD116-23;SNORD116-26;SNORD116-4;SNORD116-6;SNORD116-7;SNRPN;SNURF	-3.04
FLRT2	-2.89
SULT1C4	-2.85
BMP5	-2.82
LINC00161	-2.79
TMEM156	-2.74
SAA2;SAA2-SAA4;SAA4	-2.73
LUM	-2.7
RGS2	-2.68
LRRC17	-2.66
CASC15	-2.57
SCART1	-2.57
RASSF8	-2.56
LINC00844	-2.55
MBOAT2	-2.55
KCNJ15	-2.53
MAPK10	-2.53
AKR1D1	-2.5
PTPN13	-2.46
RXFP1	-2.41
CLEC12A	-2.37
DDX26B	-2.37
OAT	-2.37
VNN1	-2.37
ANKRD18DP;LMLN-AS1	-2.33

CDH12	4.4
TERC	4.38
LINC01484	4.32
HIST1H2AH;MIR3143	4.29
ACHE	4.27
GCNT3	4.27
NECAB2;OSGIN1	4.23
PTP4A3	4.23
MLST8	4.19
AFAP1-AS1	4.17
MAGEC3	4.13
HIST1H4A	4.1
RHPN1	3.9
LINC01124;LOC101926913	3.84
SHANK2	3.83
SCUBE1	3.74
DFNB31	3.71
HSF1	3.67
TK1	3.63
CHTF18	3.61
AURKB	3.6
WNK4	3.6
CEP131	3.58
G6PD	3.57
CLVS1	3.56
LINC01108	3.56
ADCK5	3.54
FAM83H	3.52
SHARPIN	3.51
TONSL	3.51
ALDH1L1	3.49
GPAA1	3.44
SLC52A2	3.44
HIST1H2AM	3.43
KIFC2	3.43
GLUL;LINC00272;RGSL1	3.41

BICC1	-2.32
MIR1273E	-2.31
LRAT;RBM46	-2.26
NNMT	-2.25
DACH1	-2.24
SPACA7	-2.23
ETNPPL	-2.2
CXCL8	-2.14
LECT2	-2.14
SLC25A36	-2.14
ANK3	-2.12
HTR2A;HTR2A-AS1	-2.12
ARHGAP15	-2.11
SLC8A1	-2.1
CP	-2.08
PRICKLE1	-2.07
SRGN	-2.05
F9	-2.04
MIR99AHG	-2.03
CD226	-2.02
DYNC2H1	-2.02
ASS1	-2.01
CCDC3	-2.01
ENDOD1	-1.98
LAMA2	-1.98
TC2N	-1.97
SPIRE1	-1.95
GBA3	-1.94
SLPI	-1.92
C6	-1.91
SLFN12	-1.91
ZFP1	-1.91
LOC101928304	-1.9
NR4A3	-1.88
FAM66C;FAM90A1	-1.87
IGFBP7	-1.87

AXIN2	3.39	SPATA6	-1.87
ABHD1	3.37	DLG2	-1.82
PARP10	3.34	GSTZ1	-1.8
MDGA1	3.33	CREB5	-1.77
RPS16	3.32	SAMSN1	-1.77
SLC22A18	3.32	EPGN;MTHFD2L	-1.76
TP73	3.3	TRIM22	-1.76
SLC6A9	3.26	CDC42EP3	-1.75
COL1A1	3.24	SYTL2	-1.74
CLSPN	3.23	TF	-1.74
LOC100506403;LOC101928269	3.22	ANXA1	-1.73
RPLP1	3.22	WDR72	-1.73
LOC148709	3.21	BMS1P21;MBL1P	-1.72
MIR3917;STMN1	3.21	PDK4	-1.71
BCL2L12	3.17	SCML4	-1.68
MRPL4	3.17	CYP4V2;KLKB1	-1.67
CHRM3	3.14	FSTL1;MIR198	-1.65
ASPSCR1	3.13	LRRK2	-1.65
JOSD2	3.12	UBXN8	-1.64
P2RY11;PPAN;PPAN-P2RY11	3.09	LST1	-1.63
CELSR3;MIR4793;NCKIPSD	3.07	PTPRC	-1.6
PKD1L2	3.04	CRMP1;EVC	-1.59
KIAA1875	3.03	ZNF211	-1.59
MOSPD3	3.02	CPED1	-1.58
PAK4	3	GYS2	-1.58
C19orf47	2.99	FGD4	-1.56
HGH1	2.98	MBL2	-1.53
ACSL6;MEIKIN	2.97	APOH;CEP112	-1.52
LINC01226	2.93	C5;TRAF1	-1.51
HIST1H2BF	2.92	F2R	-1.51
SLCO5A1	2.92	GNB4	-1.51
LINC01604	2.91	APOL6	-1.46
TMEM201	2.9	FGG	-1.43
MIR325HG	2.89	ADGRG6	-1.42
LOC101927136	2.87	TCF4	-1.4
CLCN7	2.85	MAN1A1	-1.34

LINC00639	2.85
NCAPH	2.84
VPS28	2.83
CLK2;SCAMP3	2.81
MIB2	2.81
FGFR4	2.8
FOXP4	2.78
IKBKG	2.75
ABCB8;ASIC3	2.74
SPPL2B	2.74
MROH6;NAPRT	2.73
NDOR1	2.73
ZNF517	2.73
GBA	2.72
RAD23A	2.72
IGSF8	2.7
GRN	2.69
PIF1	2.69
SMARCA4	2.69
ARF1	2.68
NUPR1	2.68
C14orf80	2.67
TCOF1	2.67
SNTG1	2.64
CHCHD10	2.63
ATAD3A;ATAD3B	2.62
EPHX1	2.62
RPL8;ZNF34;ZNF517	2.61
PIK3R2	2.6
GAS2L1	2.58
PKN1	2.57
PMVK	2.57
CCDC9	2.56

PHLDB2;PLCXD2	-1.33
GLUD1	-1.28
PSD3	-1.28
B2M	-1.27
PRDX4	-1.27
CD164	-1.25
ANKAR	-1.24
RPS15A	-1.18
RWDD3;TMEM56;TMEM56-RWDD3	-1.16
TSHR	-1.11
PCDHGA1;PCDHGA10;PCDHGA11;PCDHGA12;PCDHGA2;PCDHGA3;PCDHGA4;PCDHGA5;PCDHGA6;PCDHGA7;PCDHGA8;PCDHGA9;PCDHGB1;PCDHGB2;PCDHGB3;PCDHGB4;PCDHGB5;PCDHGB6;PCDHGB7;PCDHGC3;PCDHGC4;PCDHGC5	-0.95

LRRC14	2.56
CIZ1	2.53
MYH14	2.53
COPE	2.51
CTSD;IFITM10	2.5
TTLL12	2.5
AP1M1	2.49
CLPTM1	2.49
CYHR1	2.49
LMNA	2.49
NRBP2;PUF60	2.47
BREA2;ZNF707	2.45
DBP	2.45
EPPK1;PLEC	2.45
LAMTOR2	2.45
TMEM74B	2.42
TM7SF2;VPS51	2.41
DLG5	2.4
EMD	2.4
PRKCSH	2.4
CLIP3;THAP8	2.39
MIR639;TECR	2.39
GUK1	2.38
IFRD2	2.38
NCLN	2.38
NDUFS6	2.38
ATP6V0D1	2.36
JRK	2.36
EPN1	2.35
FBXO31	2.34
RSPO2	2.34
HSPA1A;HSPA1B	2.33
MTMR11;SF3B4	2.33
VARS	2.33
CTSA	2.31
SYNGR2	2.31

DCST2;LOC100505666	2.3
MAFG;SIRT7	2.29
MIR3672	2.29
NFKB2	2.29
TRIM55	2.29
ZC3H3	2.29
FAM3A	2.26
GDI1	2.25
RARA	2.25
C7orf50;COX19	2.24
FLNA	2.24
ATP13A2	2.23
DDX12P	2.23
FANCA	2.23
MAF1	2.21
SLC25A39	2.21
ZNF730	2.21
NDUFS8	2.2
MRPL38	2.19
MYO15B	2.19
RNPEPL1	2.17
ANAPC11	2.16
ASNA1	2.15
DNPH1	2.15
PLXNA1	2.15
NOTCH1	2.14
SLC4A2	2.14
TAF6	2.14
NDUFB7	2.13
PFDN6	2.13
WBP2	2.13
NIPAL2	2.12
PLXNB2	2.12
PQBP1	2.12
GPR137	2.11
CD74	2.1

GNB2	2.1
LSS	2.1
BCAR1	2.09
D2HGDH	2.09
TRAPPC9	2.09
ABHD4	2.08
SH3BP5L	2.08
TOR2A	2.08
EIF3K	2.07
ZSCAN31	2.07
ACTB	2.06
TRPM3	2.06
CDC42EP4	2.05
NCOA2	2.05
TMEM259	2.04
MYH9	2.03
RNH1	2.03
TSKU	2.03
MED15	2.01
ADHFE1;C8orf46;RRS1	1.99
ATP1A1	1.99
BAG3	1.98
GAA	1.98
FKBP5;LOC285847	1.97
RPP21;TRIM39;TRIM39-RPP21	1.96
ABCF1	1.95
IQCE	1.95
ACTN4	1.94
KDM4B	1.94
DRAP1	1.93
NINJ1	1.92
AGO2	1.91
SGK223	1.91
APOE	1.9
FDPS	1.9
MRPL28	1.9

NDUFA3	1.9
NPRL3	1.9
TMEM8A	1.9
COL18A1	1.89
H1F0	1.89
INPPL1	1.89
ETFB;VSIG10L	1.88
ICK	1.88
LOC100133315;ZNF705E	1.88
FAM20C	1.85
LMAN2	1.85
TWF2	1.85
ZBTB18	1.85
HIST1H2BJ;HIST1H2BK	1.84
ZNF513	1.84
MSH5;MSH5-SAPCD1;SAPCD1	1.83
AP3D1	1.82
09-Sep	1.81
NCL	1.81
NELFB	1.8
LGALS3BP	1.79
GSTT1	1.78
CTTN	1.77
DDX49	1.77
HNRNPA1;HNRNPA1P10	1.77
SEC13	1.76
ANKMY1	1.75
DPAGT1;H2AFX	1.75
HSP90AB1	1.75
MAPKAPK2	1.75
MIR4669;RXRA	1.75
UBA1	1.75
TRAP1	1.74
GTF2F1;PSPN	1.73
MSC-AS1	1.73
PTBP1	1.72

TALDO1	1.72
U2AF2	1.71
RPL18A	1.7
ATP6V1C1	1.69
FTL	1.68
RUVBL2	1.68
EEF1D	1.65
GPX4	1.65
LINC00189	1.65
FAAP20	1.64
DCAF7	1.63
MDH2	1.63
GPI;PDCD2L	1.62
BHLHE40	1.59
EIF4G1	1.59
TMED9	1.58
KRTCAP2	1.57
TTC13	1.57
RPS21	1.56
CYC1	1.55
CDC37;MIR1181	1.54
EIF3H	1.54
EEF2	1.51
HSPA5	1.51
CALM3	1.5
PARP1	1.5
TOMM20	1.5
ECH1	1.47
RPS2	1.46
EWSR1	1.44
HSP90AA1	1.44
DAP	1.43
ACTG1	1.42
ARIH1;MIR630	1.41
CDK11A;CDK11B	1.39
FTH1	1.39

BST2	1.37
P4HB	1.35
GAPDH	1.34
PTK2	1.34
SMC1A	1.33
PPIB	1.32
SEPHS2	1.25
NFE2L1	1.24
CASC19;CCAT1;PCAT2	1.2

Supplementary Table 9. List of regulatory genes in HCV related group

ABAT	CENPF	ERRFI1	ITGA6	PRKDC
ACAA2	CENPL	EZH2	ITGA9	PSMD4
ACLY	CENPM	FASN	ITGB3	PYCR1
ACSL1	CFL1	FGFR2	KIF23	RACGAP1
ACSM1	CFTR	FOS	KIF2C	RECQL4
ACSM3	CHEK1	FOXM1	LAMA3	SERPINH1
ACSM6	COL14A1	FXYD1	LAMC1	SHBG
ACTN2	COL19A1	FXYD3	LMNB1	SKA1
ADRA1B	COL4A3	GABARAPL1	LMNB2	SLC27A2
AGXT	COL7A1	GAD1	MCM2	SLC38A2
AKR1B10	COLEC10	GLYAT	MCM3	SLC5A1
ALDH3A1	CXCL12	GLYATL1	MCM4	SMC4
ALDOA	CYP17A1	GNAO1	MCM6	SQSTM1
ALX1	CYP19A1	GOT1	MCM8	SSRP1
AURKA	CYP1A2	GREB1	MKI67	STIP1

AURKB	CYP2B6	GRIN2B	MOGAT2	TAT
BCHE	CYP2C18	GSTA1	NAT1	TFAP2A
BIRC5	CYP2C9	HGF	NEK2	THBS1
BRCA1	CYP2E1	HGS	NME1	TKT
BRIP1	CYP3A4	HMGA1	NQO1	TOP2A
BUB1B	CYP3A43	HSD17B2	OGDHL	TPX2
CACNA1E	DCN	IGF1	P4HA2	TRPC3
CCNB1	DGAT2	IGF2	PCK1	TRPV6
CCT3	DLAT	IGF2BP1	PDGFRA	TTR
CDC25B	DNAJC6	IGF2BP3	PKM	TUBA1A
CDC25C	DYNC1I1	IGFBP3	PLA2G5	TUBA1C
CDC45	EGF	IL4I1	PLG	TUBB
CDK1	EGR1	INCENP	PPFIA4	UBE2C
CENPE	ENO1	ITGA5	PRC1	ZWINT

Supplementary Table 10. List of regulatory genes in non-viral group

A2M	DLG2	HSPA5	RUVBL2
ACTB	DYNC1I1	IGF2BP1	SEC13
ACTG1	EEF1D	IGSF8	SLC6A2
ACTN2	EEF2	IKBKG	SLC8A1
AGO2	EGR1	INPPL1	SMARCA4
AKR1B10	EIF3H	LAMA3	SNRPB
ALDH3A1	EIF3K	LMNA	SQSTM1
ALDOA	EMD	MAFG	TALDO1
AP1B1	EPN1	MCM2	TCF4
AP1M1	EWSR1	MCM4	TK1
ARF1	EZH2	MYH9	TP73
ATP1A1	F2R	NCL	TRAP1
ATP6V0D1	FANCA	NFE2L1	TUBB
AURKB	FASN	NFKB2	TWF2
BAG3	FLNC	NQO1	U2AF2
BCAR1	FOXM1	OAT	UBA1
BSG	FTH1	PARP1	
C5	GAPDH	PIK3R2	

CAMK2A	GLUD1	PRKDC	
CDC37	GNB2	PTBP1	
CDK11A	GNB4	PTK2	
COL1A1	GSTT1	RARA	
COL5A3	HGF	RECQL4	
COPE	HGS	RPL18A	
CP	HMGA1	RPL8	
CTTN	HNRNPA1	RPLP1	
CXCL8	HSF1	RPS16	
CYP17A1	HSP90AA1	RPS17	
CYP2E1	HSP90AB1	RPS2	
DCAF7	HSPA1A	RPS21	

Supplementary Table 11. Clustering of immune gene data by ClueGO

ClueGOResults

Clusters	GOID	Term	Term Pvalue, Bonferroni step down
Cluster1	GO:0002280	monocyte activation involved in immune response	0.17470
Cluster1	GO:0002780	antibacterial peptide biosynthetic process	0.24500
Cluster1	GO:0010729	positive regulation of hydrogen peroxide biosynthetic process	0.38884
Cluster1	GO:0015204	urea transmembrane transporter activity	0.42971
Cluster1	GO:0015250	water channel activity	0.19817
Cluster1	GO:0015254	glycerol channel activity	0.41472
Cluster1	GO:0015911	long-chain fatty acid import across plasma membrane	0.42740
Cluster1	GO:0021930	cerebellar granule cell precursor proliferation	0.19817
Cluster1	GO:0042167	heme catabolic process	0.41027
Cluster1	GO:0042361	menaquinone catabolic process	0.30407
Cluster1	GO:0042376	phylloquinone catabolic process	0.30407
Cluster1	GO:0042822	pyridoxal phosphate metabolic process	0.30407
Cluster1	GO:0046951	ketone body biosynthetic process	0.38884
Cluster1	GO:0050051	leukotriene-B4 20-monooxygenase activity	0.35198
Cluster1	GO:0055062	phosphate ion homeostasis	0.10893
Cluster1	GO:0070295	renal water absorption	0.38884
Cluster1	GO:0090650	cellular response to oxygen-glucose deprivation	0.35198
Cluster1	GO:1904929	coreceptor activity involved in Wnt signaling pathway, planar cell polarity pathway	0.41472
Cluster1	GO:0021533	cell differentiation in hindbrain	0.13625
Cluster1	GO:0045649	regulation of macrophage differentiation	0.13625
Cluster1	GO:1901030	positive regulation of mitochondrial outer membrane permeabilization involved in apoptotic signaling pathway	0.04912
Cluster2	GO:0046631	alpha-beta T cell activation	0.00000
Cluster2	GO:0002503	peptide antigen assembly with MHC class II protein complex	0.07648
Cluster2	GO:0002507	tolerance induction	0.00163
Cluster2	GO:0002578	negative regulation of antigen processing and presentation	0.03229
Cluster2	GO:0002643	regulation of tolerance induction	0.00749
Cluster2	GO:0002666	positive regulation of T cell tolerance induction	0.03229
Cluster2	GO:0021879	forebrain neuron differentiation	0.05618
Cluster2	GO:0021902	commitment of neuronal cell to specific neuron type in forebrain	0.01579
Cluster2	GO:0031295	T cell costimulation	0.00023
Cluster2	GO:0032660	regulation of interleukin-17 production	0.04084
Cluster2	GO:0032663	regulation of interleukin-2 production	0.00141
Cluster2	GO:0032722	positive regulation of chemokine production	0.00257
Cluster2	GO:0032743	positive regulation of interleukin-2 production	0.03240
Cluster2	GO:0032753	positive regulation of interleukin-4 production	0.01783
Cluster2	GO:0033634	positive regulation of cell-cell adhesion mediated by integrin	0.01579
Cluster2	GO:0045058	T cell selection	0.00002
Cluster2	GO:0045061	thymic T cell selection	0.00031
Cluster2	GO:0045589	regulation of regulatory T cell differentiation	0.03189
Cluster2	GO:0045954	positive regulation of natural killer cell mediated cytotoxicity	0.15046
Cluster2	GO:0046633	alpha-beta T cell proliferation	0.05525
Cluster2	GO:0046635	positive regulation of alpha-beta T cell activation	0.00204
Cluster2	GO:0046641	positive regulation of alpha-beta T cell proliferation	0.01241
Cluster2	GO:0050854	regulation of antigen receptor-mediated signaling pathway	0.01643
Cluster2	GO:0051712	positive regulation of killing of cells of another organism	0.02683
Cluster2	GO:0070229	negative regulation of lymphocyte apoptotic process	0.03936
Cluster2	GO:0070232	regulation of T cell apoptotic process	0.04523
Cluster2	GO:0071639	positive regulation of monocyte chemotactic protein-1 production	0.05690
Cluster2	GO:2000318	positive regulation of T-helper 17 type immune response	0.07111
Cluster2	GO:2000514	regulation of CD4-positive, alpha-beta T cell activation	0.00301
Cluster2	GO:2000516	positive regulation of CD4-positive, alpha-beta T cell activation	0.04523
Cluster2	GO:0002228	natural killer cell mediated immunity	0.00000
Cluster2	GO:0002715	regulation of natural killer cell mediated immunity	0.00034
Cluster3	GO:0072178	nephric duct morphogenesis	0.05562
Cluster3	GO:0001631	cysteinyl leukotriene receptor activity	0.17470
Cluster3	GO:0002003	angiotensin maturation	0.41027
Cluster3	GO:0004875	complement receptor activity	0.38260
Cluster3	GO:0004914	interleukin-5 receptor activity	0.17470
Cluster3	GO:0030862	positive regulation of polarized epithelial cell differentiation	0.10401
Cluster3	GO:0039008	pronephric nephron tubule morphogenesis	0.10401
Cluster3	GO:0045725	positive regulation of glycogen biosynthetic process	0.26740
Cluster3	GO:0045959	negative regulation of complement activation, classical pathway	0.43391
Cluster3	GO:0048633	positive regulation of skeletal muscle tissue growth	0.38884
Cluster3	GO:0060720	spongiotrophoblast cell proliferation	0.17470
Cluster3	GO:0070986	left/right axis specification	0.33646
Cluster3	GO:0071377	cellular response to glucagon stimulus	0.38260
Cluster3	GO:0090191	negative regulation of branching involved in ureteric bud morphogenesis	0.17470
Cluster3	GO:0090721	primary adaptive immune response involving T cells and B cells	0.10401
Cluster3	GO:1900025	negative regulation of substrate adhesion-dependent cell spreading	0.36998
Cluster3	GO:1905665	positive regulation of calcium ion import across plasma membrane	0.38260
Cluster3	GO:2000467	positive regulation of glycogen (starch) synthase activity	0.35198

Supplementary Figure 1. Histology (hematoxylin-eosin staining) for the HCC patients (non-viral, HBV and HCV etiology)

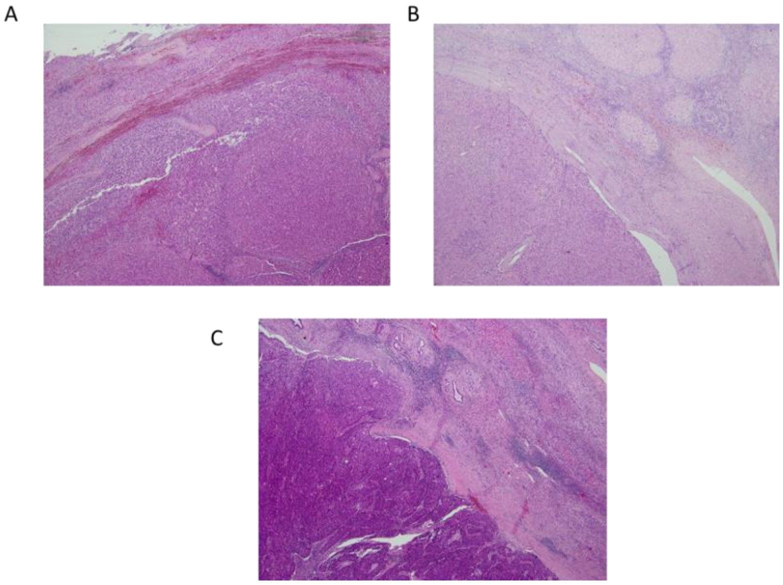

A-Non-viral HCC; B- HBV HCC; C- HCV HCC (40X magnification)

All patients enrolled in this study were diagnosed with HCC. Liver tumor samples were collected at the time of surgery. Histopathological parameters and tumor staging at diagnosis were determined (AJCC 7th ed.) and combined with surgical records and perioperative imaging. Tumor grading was performed according to the Edmondson–Steiner classification.

Supplementary Figure 2. Tuxedo pipeline

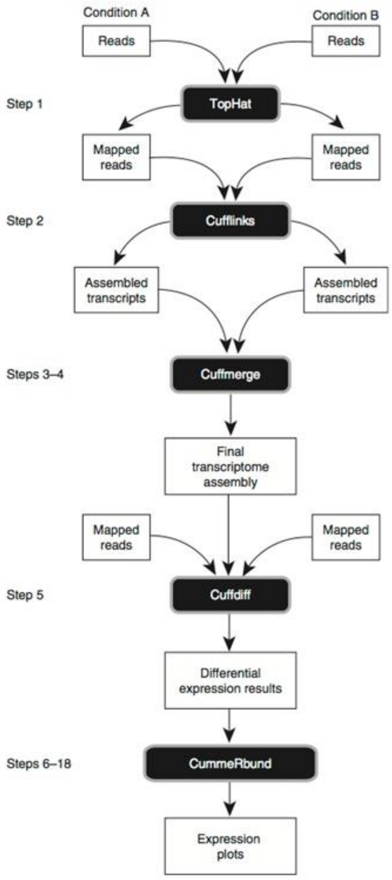

Supplementary Figure 3. HCV HUB and moonlighting genes enrichment

HCV HUB genes

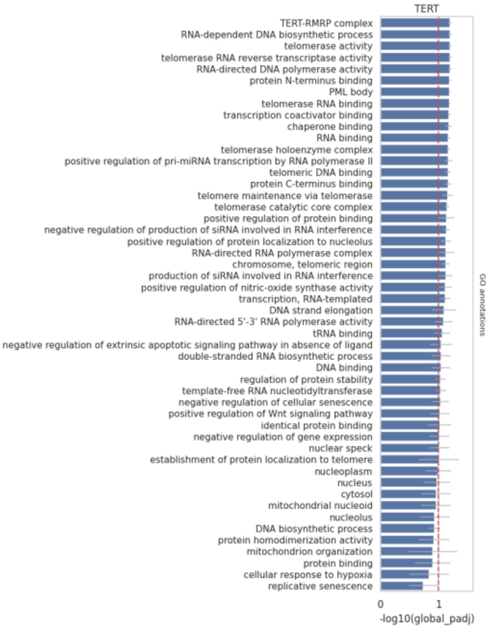

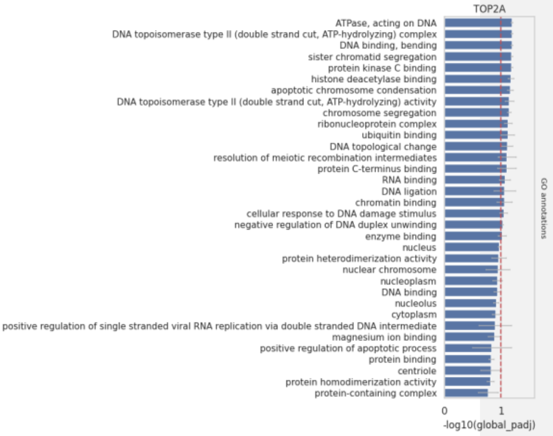

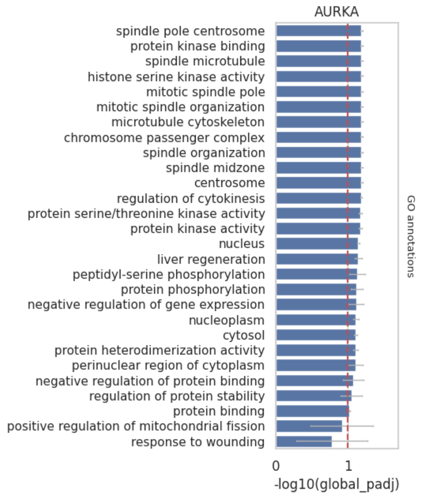
AURKA
spindle pole centrosome
protein kinase binding
spindle microtubule
histone serine kinase activity
mitotic spindle pole
mitotic spindle organization
microtubule cytoskeleton
chromosome passenger complex
spindle organization
spindle midzone
centrosome
regulation of cytokinesis
protein serine/threonine kinase activity
protein kinase activity
nucleus
liver regeneration
peptidyl-serine phosphorylation
protein phosphorylation
negative regulation of gene expression
nucleoplasm
cytosol
protein heterodimerization activity
perinuclear region of cytoplasm
negative regulation of protein binding
regulation of protein stability
protein binding
positive regulation of mitochondrial fission
response to wounding
0
1
-log10(global_padj)
GO annotations

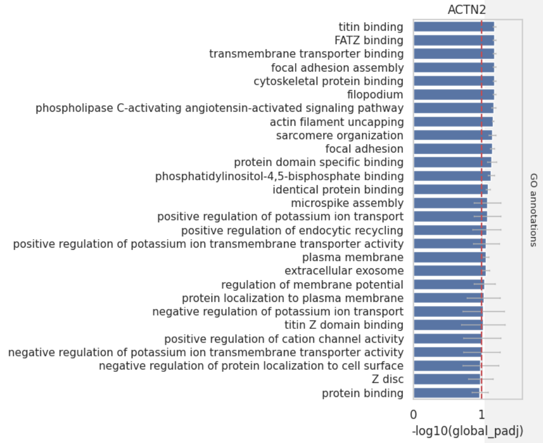
ACTN2
titin binding
FATZ binding
transmembrane transporter binding
focal adhesion assembly
cytoskeletal protein binding
filopodium
phospholipase C-activating angiotensin-activated signaling pathway
actin filament uncapping
sarcomere organization
focal adhesion
protein domain specific binding
phosphatidylinositol-4,5-bisphosphate binding
identical protein binding
microspike assembly
positive regulation of potassium ion transport
positive regulation of endocytic recycling
positive regulation of potassium ion transmembrane transporter activity
plasma membrane
extracellular exosome
regulation of membrane potential
protein localization to plasma membrane
negative regulation of potassium ion transport
titin Z domain binding
positive regulation of cation channel activity
negative regulation of potassium ion transmembrane transporter activity
negative regulation of protein localization to cell surface
Z disc
protein binding
0
1
-log10(global_padj)
GO annotations

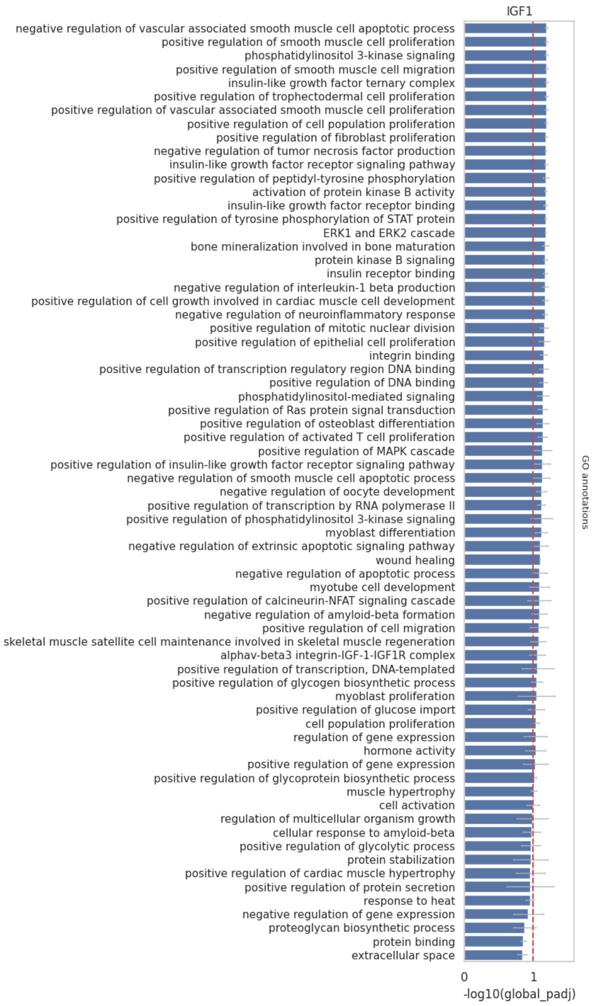
IGF1
negative regulation of vascular associated smooth muscle cell apoptotic process
positive regulation of smooth muscle cell proliferation
phosphatidylinositol 3-kinase signaling
positive regulation of smooth muscle cell migration
insulin-like growth factor ternary complex
positive regulation of trophectodermal cell proliferation
positive regulation of vascular associated smooth muscle cell proliferation
positive regulation of cell population proliferation
positive regulation of fibroblast proliferation
negative regulation of tumor necrosis factor production
insulin-like growth factor receptor signaling pathway
positive regulation of peptidyl-tyrosine phosphorylation
activation of protein kinase B activity
insulin-like growth factor receptor binding
positive regulation of tyrosine phosphorylation of STAT protein
ERK1 and ERK2 cascade
bone mineralization involved in bone maturation
protein kinase B signaling
insulin receptor binding
negative regulation of interleukin-1 beta production
positive regulation of cell growth involved in cardiac muscle cell development
negative regulation of neuroinflammatory response
positive regulation of mitotic nuclear division
positive regulation of epithelial cell proliferation
integrin binding
positive regulation of transcription regulatory region DNA binding
positive regulation of DNA binding
phosphatidylinositol-mediated signaling
positive regulation of Ras protein signal transduction
positive regulation of osteoblast differentiation
positive regulation of activated T cell proliferation
positive regulation of MAPK cascade
positive regulation of insulin-like growth factor receptor signaling pathway
negative regulation of smooth muscle cell apoptotic process
negative regulation of oocyte development
positive regulation of transcription by RNA polymerase II
positive regulation of phosphatidylinositol 3-kinase signaling
myoblast differentiation
negative regulation of extrinsic apoptotic signaling pathway
wound healing
negative regulation of apoptotic process
myotube cell development
positive regulation of calcineurin-NFAT signaling cascade
negative regulation of amyloid-beta formation
positive regulation of cell migration
skeletal muscle satellite cell maintenance involved in skeletal muscle regeneration
alphav-beta3 integrin-IGF-1-IGF1R complex
positive regulation of transcription, DNA-templated
positive regulation of glycogen biosynthetic process
myoblast proliferation
positive regulation of glucose import
cell population proliferation
regulation of gene expression
hormone activity
positive regulation of gene expression
positive regulation of glycoprotein biosynthetic process
muscle hypertrophy
cell activation
regulation of multicellular organism growth
cellular response to amyloid-beta
positive regulation of glycolytic process
protein stabilization
positive regulation of cardiac muscle hypertrophy
positive regulation of protein secretion
response to heat
negative regulation of gene expression
proteoglycan biosynthetic process
protein binding
extracellular space
GO annotations
0
1
-log10(global_padj)

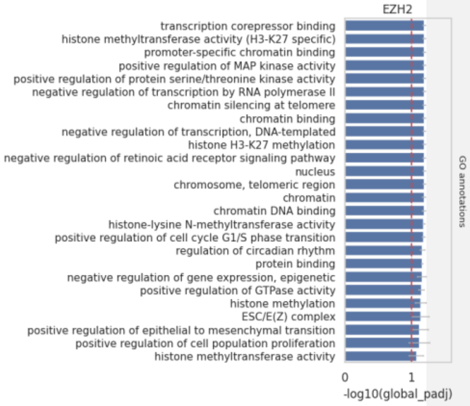
EZH2
transcription corepressor binding
histone methyltransferase activity (H3-K27 specific)
promoter-specific chromatin binding
positive regulation of MAP kinase activity
positive regulation of protein serine/threonine kinase activity
negative regulation of transcription by RNA polymerase II
chromatin silencing at telomere
chromatin binding
negative regulation of transcription, DNA-templated
histone H3-K27 methylation
negative regulation of retinoic acid receptor signaling pathway
nucleus
chromosome, telomeric region
chromatin
chromatin DNA binding
histone-lysine N-methyltransferase activity
positive regulation of cell cycle G1/S phase transition
regulation of circadian rhythm
protein binding
negative regulation of gene expression, epigenetic
positive regulation of GTPase activity
histone methylation
ESC/E(Z) complex
positive regulation of epithelial to mesenchymal transition
positive regulation of cell population proliferation
histone methyltransferase activity
GO annotations
0
1
-log10(global_padj)

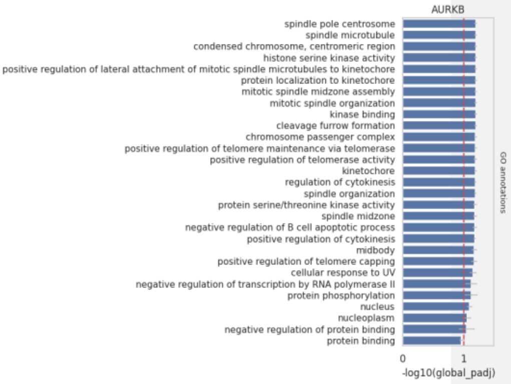
AURKB
spindle pole centrosome
spindle microtubule
condensed chromosome, centromeric region
histone serine kinase activity
positive regulation of lateral attachment of mitotic spindle microtubules to kinetochore
protein localization to kinetochore
mitotic spindle midzone assembly
mitotic spindle organization
kinase binding
cleavage furrow formation
chromosome passenger complex
positive regulation of telomere maintenance via telomerase
positive regulation of telomerase activity
kinetochore
regulation of cytokinesis
spindle organization
protein serine/threonine kinase activity
spindle midzone
negative regulation of B cell apoptotic process
positive regulation of cytokinesis
midbody
positive regulation of telomere capping
cellular response to UV
negative regulation of transcription by RNA polymerase II
protein phosphorylation
nucleus
nucleoplasm
negative regulation of protein binding
protein binding
GO annotations
0
1
-log10(global_padj)

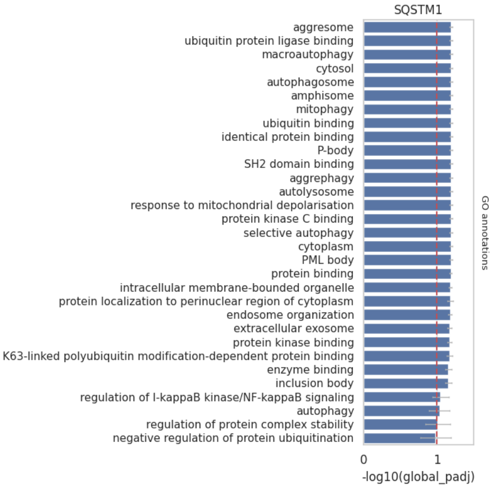
SQSTM1
aggresome
ubiquitin protein ligase binding
macroautophagy
cytosol
autophagosome
amphisome
mitophagy
ubiquitin binding
identical protein binding
P-body
SH2 domain binding
aggrephagy
autolysosome
response to mitochondrial depolarisation
protein kinase C binding
selective autophagy
cytoplasm
PML body
protein binding
intracellular membrane-bounded organelle
protein localization to perinuclear region of cytoplasm
endosome organization
extracellular exosome
protein kinase binding
K63-linked polyubiquitin modification-dependent protein binding
enzyme binding
inclusion body
regulation of I-kappaB kinase/NF-kappaB signaling
autophagy
regulation of protein complex stability
negative regulation of protein ubiquitination
GO annotations
0
1
-log10(global_padj)

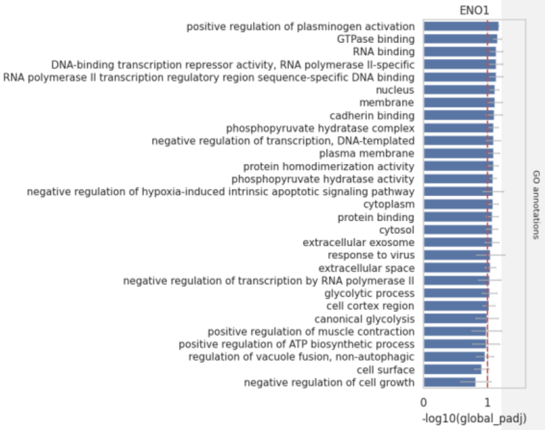
ENO1
positive regulation of plasminogen activation
GTPase binding
RNA binding
DNA-binding transcription repressor activity, RNA polymerase II-specific
RNA polymerase II transcription regulatory region sequence-specific DNA binding
nucleus
membrane
cadherin binding
phosphopyruvate hydratase complex
negative regulation of transcription, DNA-templated
plasma membrane
protein homodimerization activity
phosphopyruvate hydratase activity
negative regulation of hypoxia-induced intrinsic apoptotic signaling pathway
cytoplasm
protein binding
cytosol
extracellular exosome
response to virus
extracellular space
negative regulation of transcription by RNA polymerase II
glycolytic process
cell cortex region
canonical glycolysis
positive regulation of muscle contraction
positive regulation of ATP biosynthetic process
regulation of vacuole fusion, non-autophagic
cell surface
negative regulation of cell growth
GO annotations
0
1
-log10(global_padj)

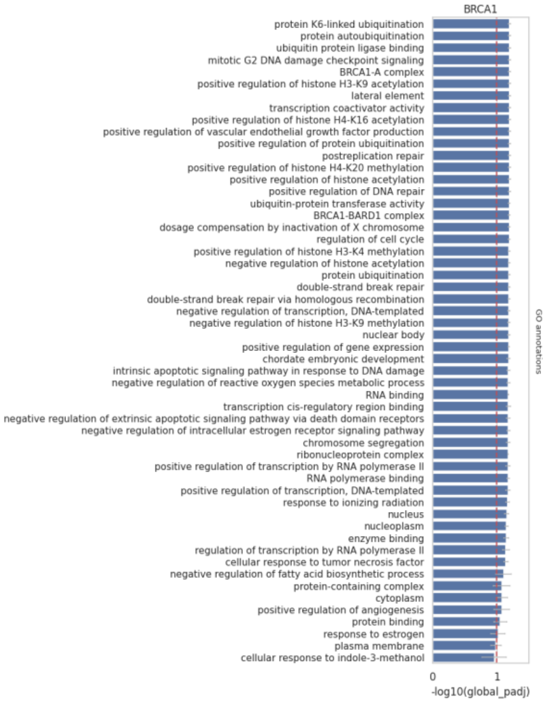
BRCA1
protein K6-linked ubiquitination
protein autoubiquitination
ubiquitin protein ligase binding
mitotic G2 DNA damage checkpoint signaling
BRCA1-A complex
positive regulation of histone H3-K9 acetylation
lateral element
transcription coactivator activity
positive regulation of histone H4-K16 acetylation
positive regulation of vascular endothelial growth factor production
positive regulation of protein ubiquitination
postreplication repair
positive regulation of histone H4-K20 methylation
positive regulation of histone acetylation
positive regulation of DNA repair
ubiquitin-protein transferase activity
BRCA1-BARD1 complex
dosage compensation by inactivation of X chromosome
regulation of cell cycle
positive regulation of histone H3-K4 methylation
negative regulation of histone acetylation
protein ubiquitination
double-strand break repair
double-strand break repair via homologous recombination
negative regulation of transcription, DNA-templated
negative regulation of histone H3-K9 methylation
nuclear body
positive regulation of gene expression
chordate embryonic development
intrinsic apoptotic signaling pathway in response to DNA damage
negative regulation of reactive oxygen species metabolic process
RNA binding
transcription cis-regulatory region binding
negative regulation of extrinsic apoptotic signaling pathway via death domain receptors
negative regulation of intracellular estrogen receptor signaling pathway
chromosome segregation
ribonucleoprotein complex
positive regulation of transcription by RNA polymerase II
RNA polymerase binding
positive regulation of transcription, DNA-templated
response to ionizing radiation
nucleus
nucleoplasm
enzyme binding
regulation of transcription by RNA polymerase II
cellular response to tumor necrosis factor
negative regulation of fatty acid biosynthetic process
protein-containing complex
cytoplasm
positive regulation of angiogenesis
protein binding
response to estrogen
plasma membrane
cellular response to indole-3-methanol
GO annotations
0
1
-log10(global_padj)

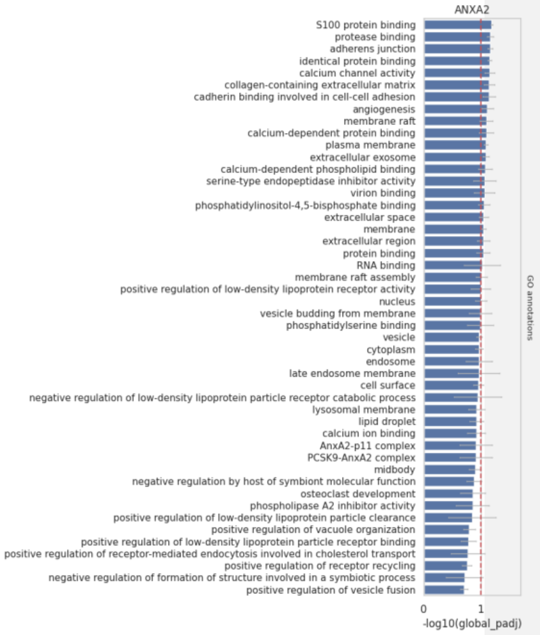
ANXA2
S100 protein binding
protease binding
adherens junction
identical protein binding
calcium channel activity
collagen-containing extracellular matrix
cadherin binding involved in cell-cell adhesion
angiogenesis
membrane raft
calcium-dependent protein binding
plasma membrane
extracellular exosome
calcium-dependent phospholipid binding
serine-type endopeptidase inhibitor activity
virion binding
phosphatidylinositol-4,5-bisphosphate binding
extracellular space
membrane
extracellular region
protein binding
RNA binding
membrane raft assembly
positive regulation of low-density lipoprotein receptor activity
nucleus
vesicle budding from membrane
phosphatidylserine binding
vesicle
cytoplasm
endosome
late endosome membrane
cell surface
negative regulation of low-density lipoprotein particle receptor catabolic process
lysosomal membrane
lipid droplet
calcium ion binding
AnxA2-p11 complex
PCSK9-AnxA2 complex
midbody
negative regulation by host of symbiont molecular function
osteoclast development
phospholipase A2 inhibitor activity
positive regulation of low-density lipoprotein particle clearance
positive regulation of vacuole organization
positive regulation of low-density lipoprotein particle receptor binding
positive regulation of receptor-mediated endocytosis involved in cholesterol transport
positive regulation of receptor recycling
negative regulation of formation of structure involved in a symbiotic process
positive regulation of vesicle fusion
GO annotations
0
1
-log10(global_padj)

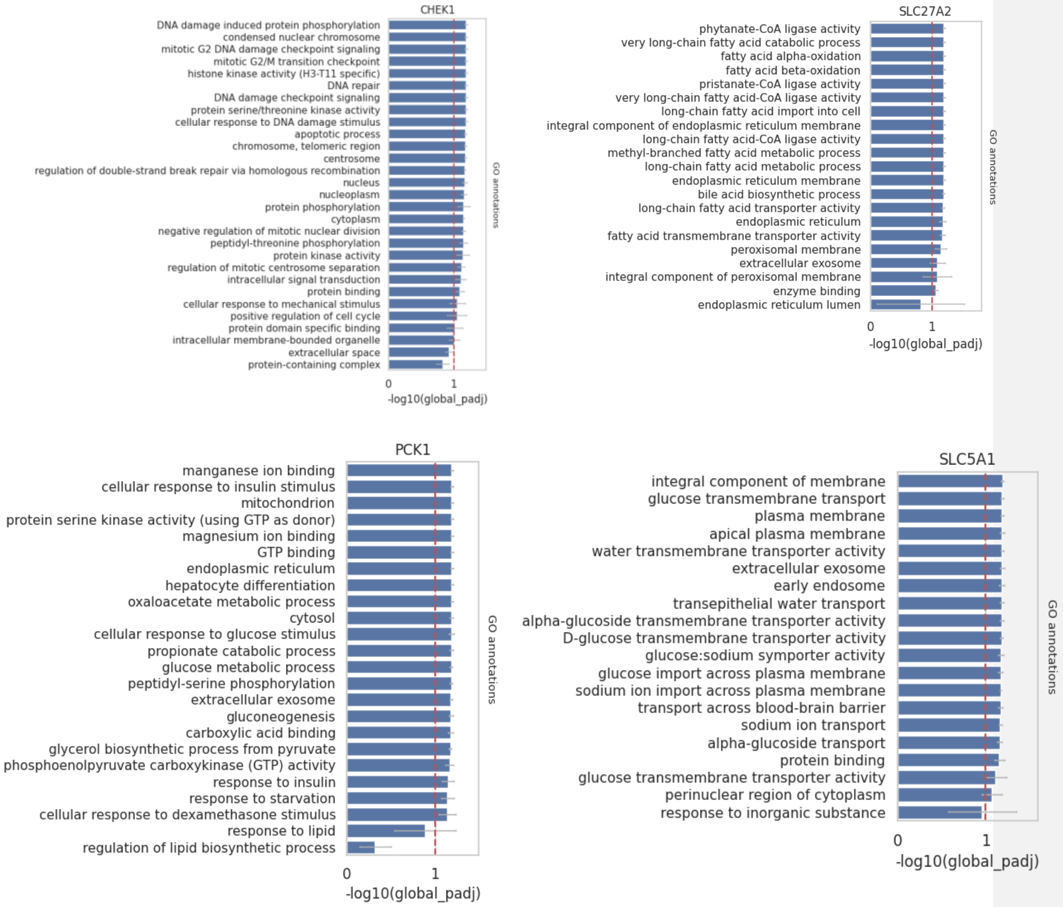
CHEK1
DNA damage induced protein phosphorylation
condensed nuclear chromosome
mitotic G2 DNA damage checkpoint signaling
mitotic G2/M transition checkpoint
histone kinase activity (H3-T11 specific)
DNA repair
DNA damage checkpoint signaling
protein serine/threonine kinase activity
cellular response to DNA damage stimulus
apoptotic process
chromosome, telomeric region
centrosome
regulation of double-strand break repair via homologous recombination
nucleus
nucleoplasm
protein phosphorylation
cytoplasm
negative regulation of mitotic nuclear division
peptidyl-threonine phosphorylation
protein kinase activity
regulation of mitotic centrosome separation
intracellular signal transduction
protein binding
cellular response to mechanical stimulus
positive regulation of cell cycle
protein domain specific binding
intracellular membrane-bounded organelle
extracellular space
protein-containing complex
0
1
-log10(global_padj)
GO annotations
SLC27A2
phytanate-CoA ligase activity
very long-chain fatty acid catabolic process
fatty acid alpha-oxidation
fatty acid beta-oxidation
pristanate-CoA ligase activity
very long-chain fatty acid-CoA ligase activity
long-chain fatty acid import into cell
integral component of endoplasmic reticulum membrane
long-chain fatty acid-CoA ligase activity
methyl-branched fatty acid metabolic process
long-chain fatty acid metabolic process
endoplasmic reticulum membrane
bile acid biosynthetic process
long-chain fatty acid transporter activity
endoplasmic reticulum
fatty acid transmembrane transporter activity
peroxisomal membrane
extracellular exosome
integral component of peroxisomal membrane
enzyme binding
endoplasmic reticulum lumen
0
1
-log10(global_padj)
GO annotations
PCK1
manganese ion binding
cellular response to insulin stimulus
mitochondrion
protein serine kinase activity (using GTP as donor)
magnesium ion binding
GTP binding
endoplasmic reticulum
hepatocyte differentiation
oxaloacetate metabolic process
cytosol
cellular response to glucose stimulus
propionate catabolic process
glucose metabolic process
peptidyl-serine phosphorylation
extracellular exosome
gluconeogenesis
carboxylic acid binding
glycerol biosynthetic process from pyruvate
phosphoenolpyruvate carboxykinase (GTP) activity
response to insulin
response to starvation
cellular response to dexamethasone stimulus
response to lipid
regulation of lipid biosynthetic process
0
1
-log10(global_padj)
GO annotations
SLC5A1
integral component of membrane
glucose transmembrane transport
plasma membrane
apical plasma membrane
water transmembrane transporter activity
extracellular exosome
early endosome
transepithelial water transport
alpha-glucoside transmembrane transporter activity
D-glucose transmembrane transporter activity
glucose:sodium symporter activity
glucose import across plasma membrane
sodium ion import across plasma membrane
transport across blood-brain barrier
sodium ion transport
alpha-glucoside transport
protein binding
glucose transmembrane transporter activity
perinuclear region of cytoplasm
response to inorganic substance
0
1
-log10(global_padj)
GO annotations

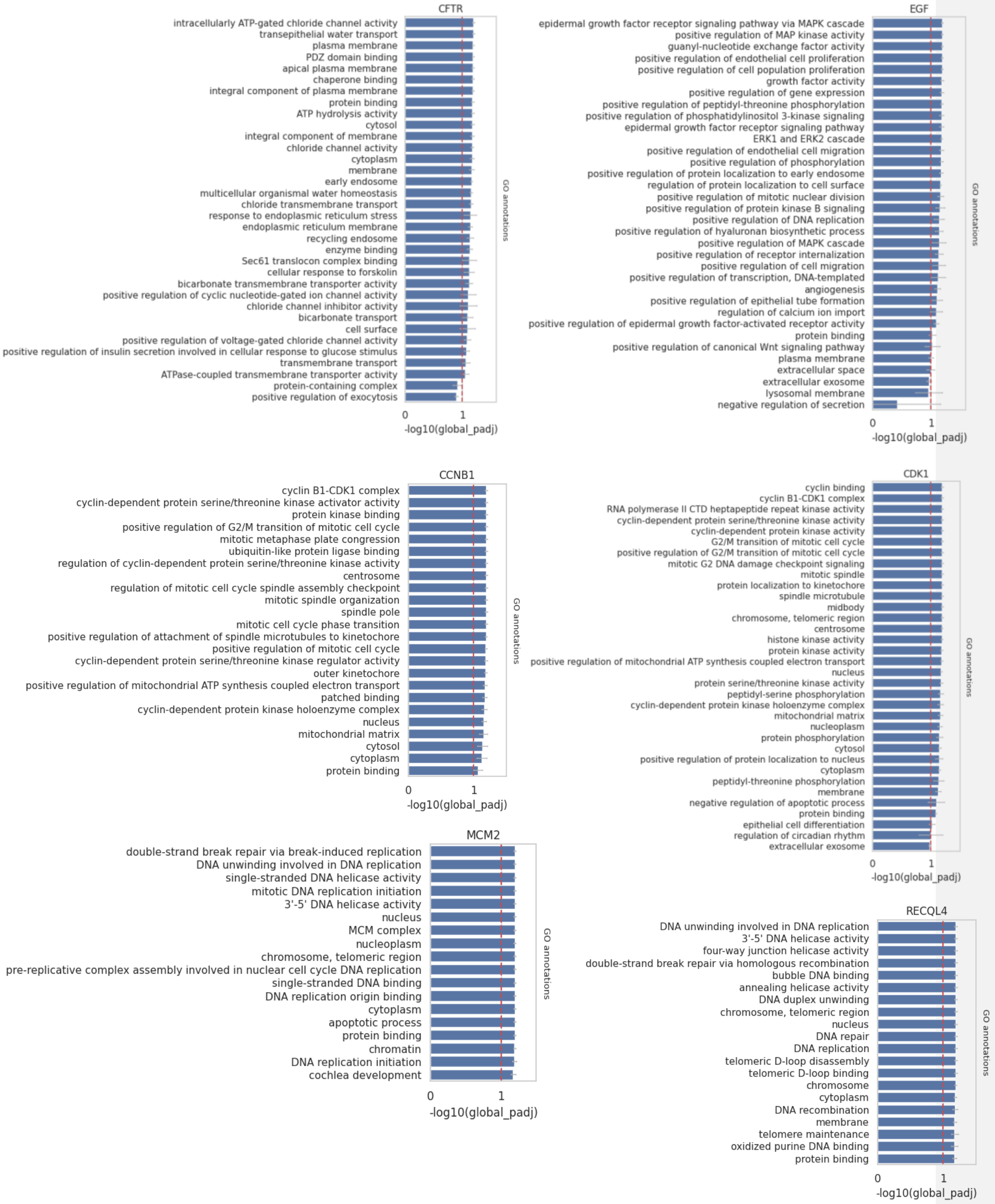
CFTR
intracellularly ATP-gated chloride channel activity
transepithelial water transport
plasma membrane
PDZ domain binding
apical plasma membrane
chaperone binding
integral component of plasma membrane
protein binding
ATP hydrolysis activity
cytosol
integral component of membrane
chloride channel activity
cytoplasm
membrane
early endosome
multicellular organismal water homeostasis
chloride transmembrane transport
response to endoplasmic reticulum stress
endoplasmic reticulum membrane
recycling endosome
enzyme binding
Sec61 translocon complex binding
cellular response to forskolin
bicarbonate transmembrane transporter activity
positive regulation of cyclic nucleotide-gated ion channel activity
chloride channel inhibitor activity
bicarbonate transport
cell surface
positive regulation of voltage-gated chloride channel activity
positive regulation of insulin secretion involved in cellular response to glucose stimulus
transmembrane transport
ATPase-coupled transmembrane transporter activity
protein-containing complex
positive regulation of exocytosis
0
1
-log10(global_padj)
GO annotations
EGF
epidermal growth factor receptor signaling pathway via MAPK cascade
positive regulation of MAP kinase activity
guanyl-nucleotide exchange factor activity
positive regulation of endothelial cell proliferation
positive regulation of cell population proliferation
growth factor activity
positive regulation of gene expression
positive regulation of peptidyl-threonine phosphorylation
positive regulation of phosphatidylinositol 3-kinase signaling
epidermal growth factor receptor signaling pathway
ERK1 and ERK2 cascade
positive regulation of endothelial cell migration
positive regulation of phosphorylation
positive regulation of protein localization to early endosome
regulation of protein localization to cell surface
positive regulation of mitotic nuclear division
positive regulation of protein kinase B signaling
positive regulation of DNA replication
positive regulation of hyaluronan biosynthetic process
positive regulation of MAPK cascade
positive regulation of receptor internalization
positive regulation of cell migration
positive regulation of transcription, DNA-templated
angiogenesis
positive regulation of epithelial tube formation
regulation of calcium ion import
positive regulation of epidermal growth factor-activated receptor activity
protein binding
positive regulation of canonical Wnt signaling pathway
plasma membrane
extracellular space
extracellular exosome
lysosomal membrane
negative regulation of secretion
0
1
-log10(global_padj)
GO annotations
CCNB1
cyclin B1-CDK1 complex
cyclin-dependent protein serine/threonine kinase activator activity
protein kinase binding
positive regulation of G2/M transition of mitotic cell cycle
mitotic metaphase plate congression
ubiquitin-like protein ligase binding
regulation of cyclin-dependent protein serine/threonine kinase activity
centrosome
regulation of mitotic cell cycle spindle assembly checkpoint
mitotic spindle organization
spindle pole
mitotic cell cycle phase transition
positive regulation of attachment of spindle microtubules to kinetochore
positive regulation of mitotic cell cycle
cyclin-dependent protein serine/threonine kinase regulator activity
outer kinetochore
positive regulation of mitochondrial ATP synthesis coupled electron transport
patched binding
cyclin-dependent protein kinase holoenzyme complex
nucleus
mitochondrial matrix
cytosol
cytoplasm
protein binding
0
1
-log10(global_padj)
GO annotations
CDK1
cyclin binding
cyclin B1-CDK1 complex
RNA polymerase II CTD heptapeptide repeat kinase activity
cyclin-dependent protein serine/threonine kinase activity
cyclin-dependent protein kinase activity
G2/M transition of mitotic cell cycle
positive regulation of G2/M transition of mitotic cell cycle
mitotic G2 DNA damage checkpoint signaling
mitotic spindle
protein localization to kinetochore
spindle microtubule
midbody
chromosome, telomeric region
centrosome
histone kinase activity
protein kinase activity
positive regulation of mitochondrial ATP synthesis coupled electron transport
nucleus
protein serine/threonine kinase activity
peptidyl-serine phosphorylation
cyclin-dependent protein kinase holoenzyme complex
mitochondrial matrix
nucleoplasm
protein phosphorylation
cytosol
positive regulation of protein localization to nucleus
cytoplasm
peptidyl-threonine phosphorylation
membrane
negative regulation of apoptotic process
protein binding
epithelial cell differentiation
regulation of circadian rhythm
extracellular exosome
0
1
-log10(global_padj)
GO annotations
MCM2
double-strand break repair via break-induced replication
DNA unwinding involved in DNA replication
single-stranded DNA helicase activity
mitotic DNA replication initiation
3'-5' DNA helicase activity
nucleus
MCM complex
nucleoplasm
chromosome, telomeric region
pre-replicative complex assembly involved in nuclear cell cycle DNA replication
single-stranded DNA binding
DNA replication origin binding
cytoplasm
apoptotic process
protein binding
chromatin
DNA replication initiation
cochlea development
0
1
-log10(global_padj)
GO annotations
RECQL4
DNA unwinding involved in DNA replication
3'-5' DNA helicase activity
four-way junction helicase activity
double-strand break repair via homologous recombination
bubble DNA binding
annealing helicase activity
DNA duplex unwinding
chromosome, telomeric region
nucleus
DNA repair
DNA replication
telomeric D-loop disassembly
telomeric D-loop binding
chromosome
cytoplasm
DNA recombination
membrane
telomere maintenance
oxidized purine DNA binding
protein binding
0
1
-log10(global_padj)
GO annotations

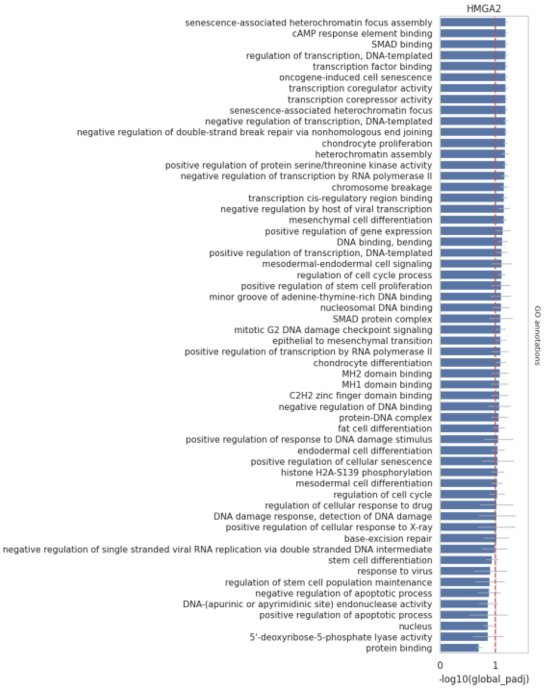
HMGA2
senescence-associated heterochromatin focus assembly
cAMP response element binding
SMAD binding
regulation of transcription, DNA-templated
transcription factor binding
oncogene-induced cell senescence
transcription coregulator activity
transcription corepressor activity
senescence-associated heterochromatin focus
negative regulation of transcription, DNA-templated
negative regulation of double-strand break repair via nonhomologous end joining
chondrocyte proliferation
heterochromatin assembly
positive regulation of protein serine/threonine kinase activity
negative regulation of transcription by RNA polymerase II
chromosome breakage
transcription cis-regulatory region binding
negative regulation by host of viral transcription
mesenchymal cell differentiation
positive regulation of gene expression
DNA binding, bending
positive regulation of transcription, DNA-templated
mesodermal-endodermal cell signaling
regulation of cell cycle process
positive regulation of stem cell proliferation
minor groove of adenine-thymine-rich DNA binding
nucleosomal DNA binding
SMAD protein complex
mitotic G2 DNA damage checkpoint signaling
epithelial to mesenchymal transition
positive regulation of transcription by RNA polymerase II
chondrocyte differentiation
MH2 domain binding
MH1 domain binding
C2H2 zinc finger domain binding
negative regulation of DNA binding
protein-DNA complex
fat cell differentiation
positive regulation of response to DNA damage stimulus
endodermal cell differentiation
positive regulation of cellular senescence
histone H2A-S139 phosphorylation
mesodermal cell differentiation
regulation of cell cycle
regulation of cellular response to drug
DNA damage response, detection of DNA damage
positive regulation of cellular response to X-ray
base-excision repair
negative regulation of single stranded viral RNA replication via double stranded DNA intermediate
stem cell differentiation
response to virus
regulation of stem cell population maintenance
negative regulation of apoptotic process
DNA-(apurinic or apyrimidinic site) endonuclease activity
positive regulation of apoptotic process
nucleus
5'-deoxyribose-5-phosphate lyase activity
protein binding
0
1
-log10(global_padj)
GO annotations

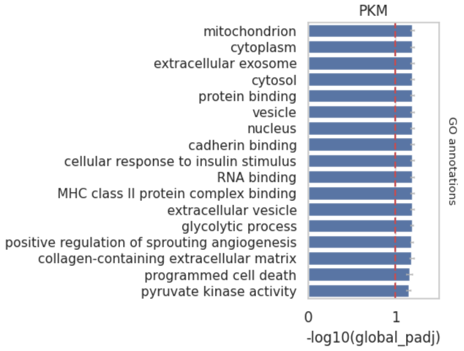
PKM
mitochondrion
cytoplasm
extracellular exosome
cytosol
protein binding
vesicle
nucleus
cadherin binding
cellular response to insulin stimulus
RNA binding
MHC class II protein complex binding
extracellular vesicle
glycolytic process
positive regulation of sprouting angiogenesis
collagen-containing extracellular matrix
programmed cell death
pyruvate kinase activity
GO annotations
0
1
-log10(global_padj)

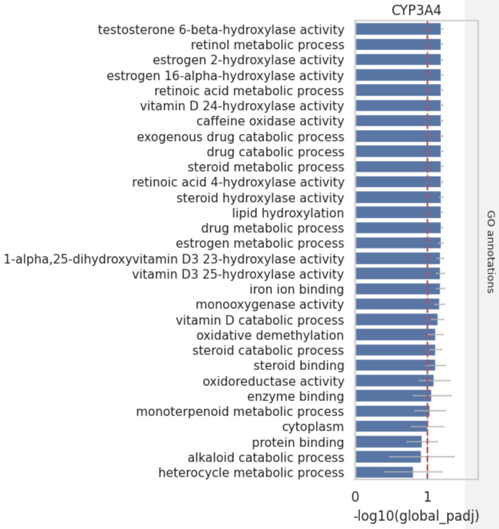
CYP3A4
testosterone 6-beta-hydroxylase activity
retinol metabolic process
estrogen 2-hydroxylase activity
estrogen 16-alpha-hydroxylase activity
retinoic acid metabolic process
vitamin D 24-hydroxylase activity
caffeine oxidase activity
exogenous drug catabolic process
drug catabolic process
steroid metabolic process
retinoic acid 4-hydroxylase activity
steroid hydroxylase activity
lipid hydroxylation
drug metabolic process
estrogen metabolic process
1-alpha,25-dihydroxyvitamin D3 23-hydroxylase activity
vitamin D3 25-hydroxylase activity
iron ion binding
monooxygenase activity
vitamin D catabolic process
oxidative demethylation
steroid catabolic process
steroid binding
oxidoreductase activity
enzyme binding
monoterpenoid metabolic process
cytoplasm
protein binding
alkaloid catabolic process
heterocycle metabolic process
GO annotations
0
1
-log10(global_padj)

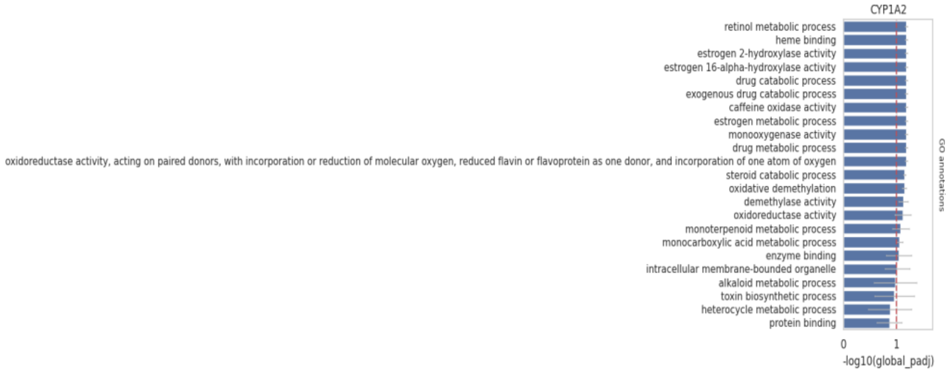
CYP1A2
retinol metabolic process
heme binding
estrogen 2-hydroxylase activity
estrogen 16-alpha-hydroxylase activity
drug catabolic process
exogenous drug catabolic process
caffeine oxidase activity
estrogen metabolic process
monooxygenase activity
drug metabolic process
oxidoreductase activity, acting on paired donors, with incorporation or reduction of molecular oxygen, reduced flavin or flavoprotein as one donor, and incorporation of one atom of oxygen
steroid catabolic process
oxidative demethylation
demethylase activity
oxidoreductase activity
monoterpenoid metabolic process
monocarboxylic acid metabolic process
enzyme binding
intracellular membrane-bounded organelle
alkaloid metabolic process
toxin biosynthetic process
heterocycle metabolic process
protein binding
GO annotations
0
1
-log10(global_padj)

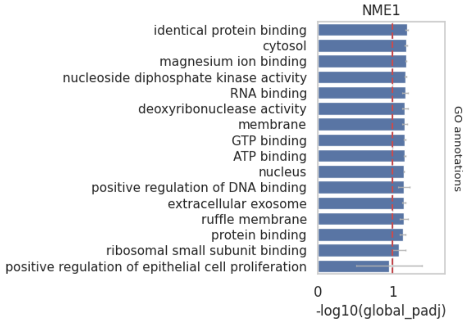
NME1
identical protein binding
cytosol
magnesium ion binding
nucleoside diphosphate kinase activity
RNA binding
deoxyribonuclease activity
membrane
GTP binding
ATP binding
nucleus
positive regulation of DNA binding
extracellular exosome
ruffle membrane
protein binding
ribosomal small subunit binding
positive regulation of epithelial cell proliferation
GO annotations
0
1
-log10(global_padj)

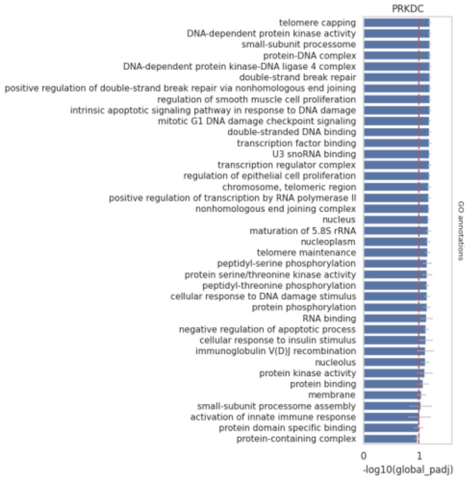
PRKDC
telomere capping
DNA-dependent protein kinase activity
small-subunit processome
protein-DNA complex
DNA-dependent protein kinase-DNA ligase 4 complex
double-strand break repair
positive regulation of double-strand break repair via nonhomologous end joining
regulation of smooth muscle cell proliferation
intrinsic apoptotic signaling pathway in response to DNA damage
mitotic G1 DNA damage checkpoint signaling
double-stranded DNA binding
transcription factor binding
U3 snoRNA binding
transcription regulator complex
regulation of epithelial cell proliferation
chromosome, telomeric region
positive regulation of transcription by RNA polymerase II
nonhomologous end joining complex
nucleus
maturation of 5.8S rRNA
nucleoplasm
telomere maintenance
peptidyl-serine phosphorylation
protein serine/threonine kinase activity
peptidyl-threonine phosphorylation
cellular response to DNA damage stimulus
protein phosphorylation
RNA binding
negative regulation of apoptotic process
cellular response to insulin stimulus
immunoglobulin V(D)J recombination
nucleolus
protein kinase activity
protein binding
membrane
small-subunit processome assembly
activation of innate immune response
protein domain specific binding
protein-containing complex
GO annotations
0
1
-log10(global_padj)

HCV Moonlightning gene

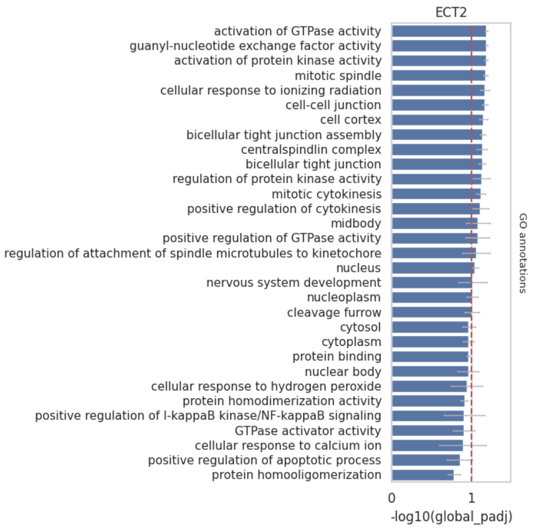

Supplementary Figure 4. nonBnonC HUB and moonlighting genes enrichment

nonBnonC HUB genes

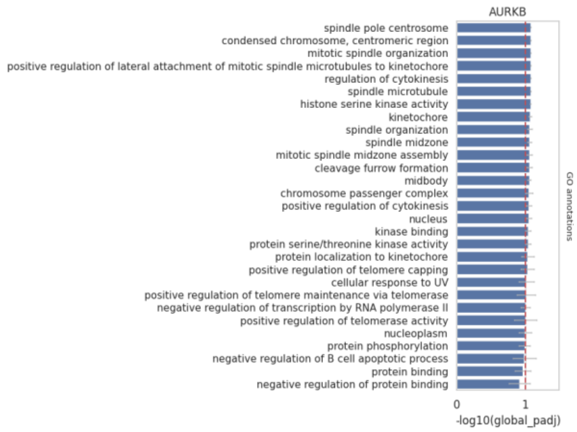

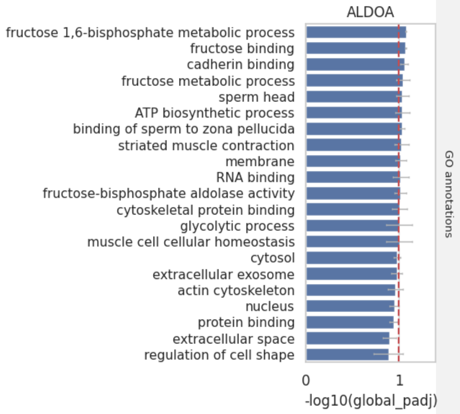

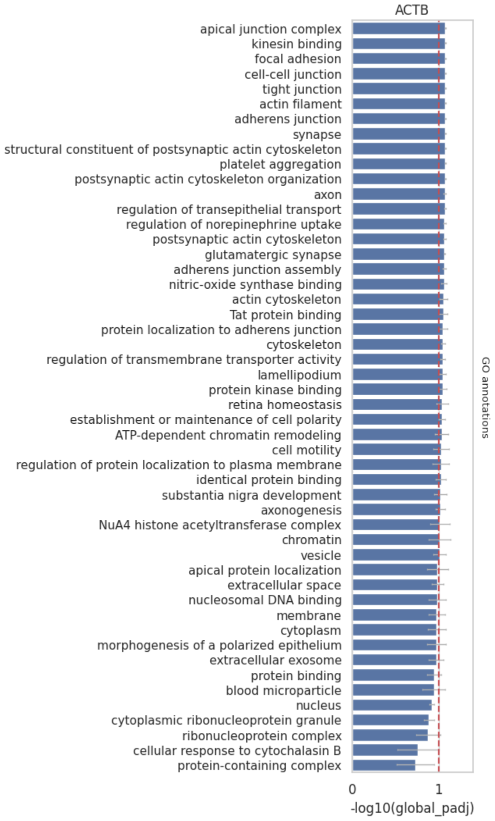
ACTB
apical junction complex
kinesin binding
focal adhesion
cell-cell junction
tight junction
actin filament
adherens junction
synapse
structural constituent of postsynaptic actin cytoskeleton
platelet aggregation
postsynaptic actin cytoskeleton organization
axon
regulation of transepithelial transport
regulation of norepinephrine uptake
postsynaptic actin cytoskeleton
glutamatergic synapse
adherens junction assembly
nitric-oxide synthase binding
actin cytoskeleton
Tat protein binding
protein localization to adherens junction
cytoskeleton
regulation of transmembrane transporter activity
lamellipodium
protein kinase binding
retina homeostasis
establishment or maintenance of cell polarity
ATP-dependent chromatin remodeling
cell motility
regulation of protein localization to plasma membrane
identical protein binding
substantia nigra development
axonogenesis
NuA4 histone acetyltransferase complex
chromatin
vesicle
apical protein localization
extracellular space
nucleosomal DNA binding
membrane
cytoplasm
morphogenesis of a polarized epithelium
extracellular exosome
protein binding
blood microparticle
nucleus
cytoplasmic ribonucleoprotein granule
ribonucleoprotein complex
cellular response to cytochalasin B
protein-containing complex
0
1
-log10(global_padj)
GO annotations

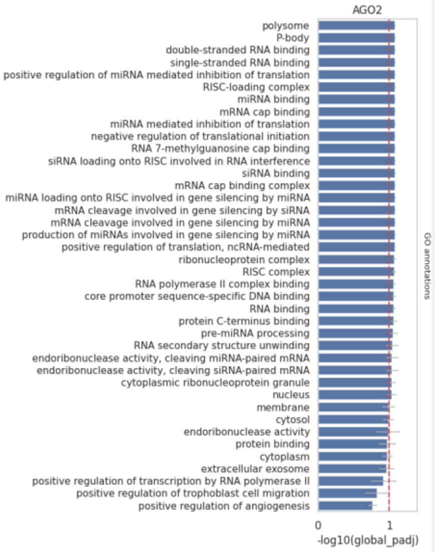
AGO2
polysome
P-body
double-stranded RNA binding
single-stranded RNA binding
positive regulation of miRNA mediated inhibition of translation
RISC-loading complex
miRNA binding
mRNA cap binding
miRNA mediated inhibition of translation
negative regulation of translational initiation
RNA 7-methylguanosine cap binding
siRNA loading onto RISC involved in RNA interference
siRNA binding
mRNA cap binding complex
miRNA loading onto RISC involved in gene silencing by miRNA
mRNA cleavage involved in gene silencing by siRNA
mRNA cleavage involved in gene silencing by miRNA
production of miRNAs involved in gene silencing by miRNA
positive regulation of translation, ncRNA-mediated
ribonucleoprotein complex
RISC complex
RNA polymerase II complex binding
core promoter sequence-specific DNA binding
RNA binding
protein C-terminus binding
pre-miRNA processing
RNA secondary structure unwinding
endoribonuclease activity, cleaving miRNA-paired mRNA
endoribonuclease activity, cleaving siRNA-paired mRNA
cytoplasmic ribonucleoprotein granule
nucleus
membrane
cytosol
endoribonuclease activity
protein binding
cytoplasm
extracellular exosome
positive regulation of transcription by RNA polymerase II
positive regulation of trophoblast cell migration
positive regulation of angiogenesis
0
1
-log10(global_padj)
GO annotations

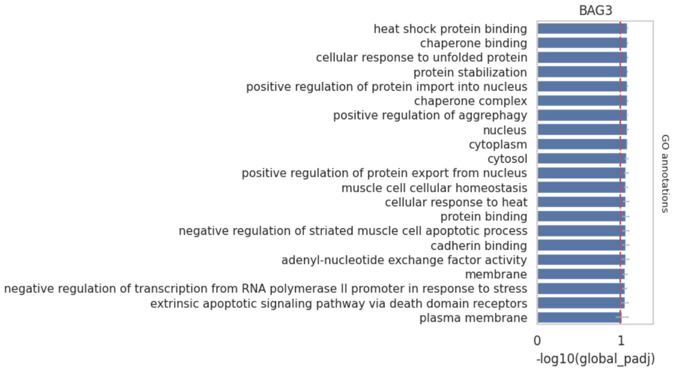
BAG3
heat shock protein binding
chaperone binding
cellular response to unfolded protein
protein stabilization
positive regulation of protein import into nucleus
chaperone complex
positive regulation of aggrephagy
nucleus
cytoplasm
cytosol
positive regulation of protein export from nucleus
muscle cell cellular homeostasis
cellular response to heat
protein binding
negative regulation of striated muscle cell apoptotic process
cadherin binding
adenyl-nucleotide exchange factor activity
membrane
negative regulation of transcription from RNA polymerase II promoter in response to stress
extrinsic apoptotic signaling pathway via death domain receptors
plasma membrane
0
1
-log10(global_padj)
GO annotations

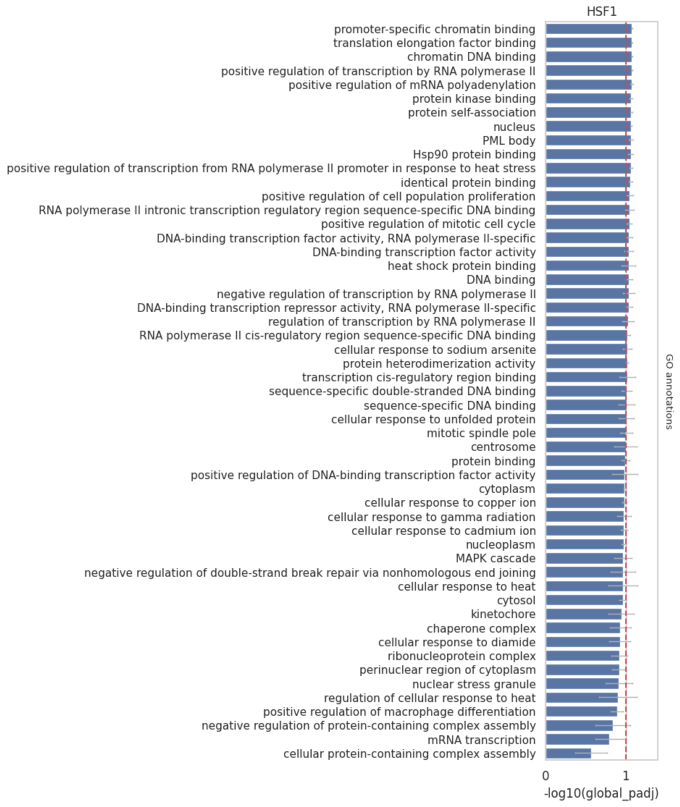
HSF1
promoter-specific chromatin binding
translation elongation factor binding
chromatin DNA binding
positive regulation of transcription by RNA polymerase II
positive regulation of mRNA polyadenylation
protein kinase binding
protein self-association
nucleus
PML body
Hsp90 protein binding
positive regulation of transcription from RNA polymerase II promoter in response to heat stress
identical protein binding
positive regulation of cell population proliferation
RNA polymerase II intronic transcription regulatory region sequence-specific DNA binding
positive regulation of mitotic cell cycle
DNA-binding transcription factor activity, RNA polymerase II-specific
DNA-binding transcription factor activity
heat shock protein binding
DNA binding
negative regulation of transcription by RNA polymerase II
DNA-binding transcription repressor activity, RNA polymerase II-specific
regulation of transcription by RNA polymerase II
RNA polymerase II cis-regulatory region sequence-specific DNA binding
cellular response to sodium arsenite
protein heterodimerization activity
transcription cis-regulatory region binding
sequence-specific double-stranded DNA binding
sequence-specific DNA binding
cellular response to unfolded protein
mitotic spindle pole
centrosome
protein binding
positive regulation of DNA-binding transcription factor activity
cytoplasm
cellular response to copper ion
cellular response to gamma radiation
cellular response to cadmium ion
nucleoplasm
MAPK cascade
negative regulation of double-strand break repair via nonhomologous end joining
cellular response to heat
cytosol
kinetochore
chaperone complex
cellular response to diamide
ribonucleoprotein complex
perinuclear region of cytoplasm
nuclear stress granule
regulation of cellular response to heat
positive regulation of macrophage differentiation
negative regulation of protein-containing complex assembly
mRNA transcription
cellular protein-containing complex assembly
0
1
-log10(global_padj)
GO annotations

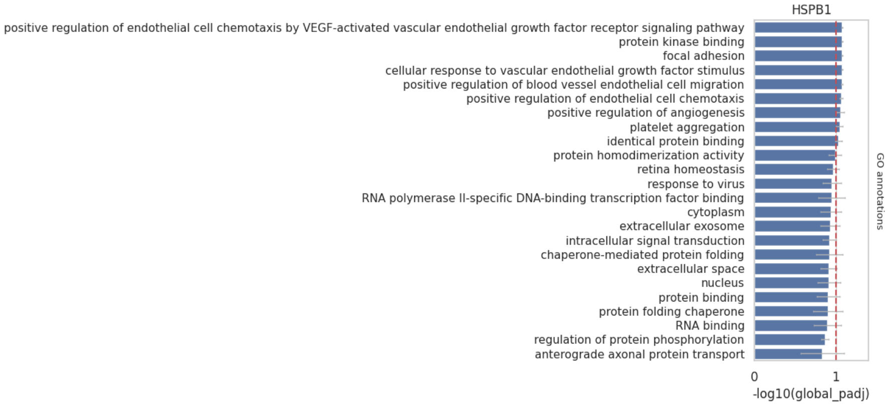
HSPB1
positive regulation of endothelial cell chemotaxis by VEGF-activated vascular endothelial growth factor receptor signaling pathway
protein kinase binding
focal adhesion
cellular response to vascular endothelial growth factor stimulus
positive regulation of blood vessel endothelial cell migration
positive regulation of endothelial cell chemotaxis
positive regulation of angiogenesis
platelet aggregation
identical protein binding
protein homodimerization activity
retina homeostasis
response to virus
RNA polymerase II-specific DNA-binding transcription factor binding
cytoplasm
extracellular exosome
intracellular signal transduction
chaperone-mediated protein folding
extracellular space
nucleus
protein binding
protein folding chaperone
RNA binding
regulation of protein phosphorylation
anterograde axonal protein transport
0
1
-log10(global_padj)
GO annotations

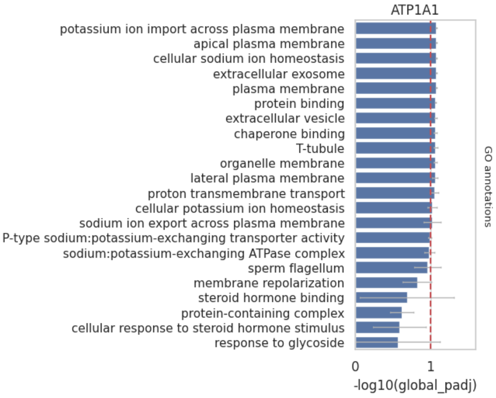
ATP1A1
potassium ion import across plasma membrane
apical plasma membrane
cellular sodium ion homeostasis
extracellular exosome
plasma membrane
protein binding
extracellular vesicle
chaperone binding
T-tubule
organelle membrane
lateral plasma membrane
proton transmembrane transport
cellular potassium ion homeostasis
sodium ion export across plasma membrane
P-type sodium:potassium-exchanging transporter activity
sodium:potassium-exchanging ATPase complex
sperm flagellum
membrane repolarization
steroid hormone binding
protein-containing complex
cellular response to steroid hormone stimulus
response to glycoside
0
1
-log10(global_padj)
GO annotations

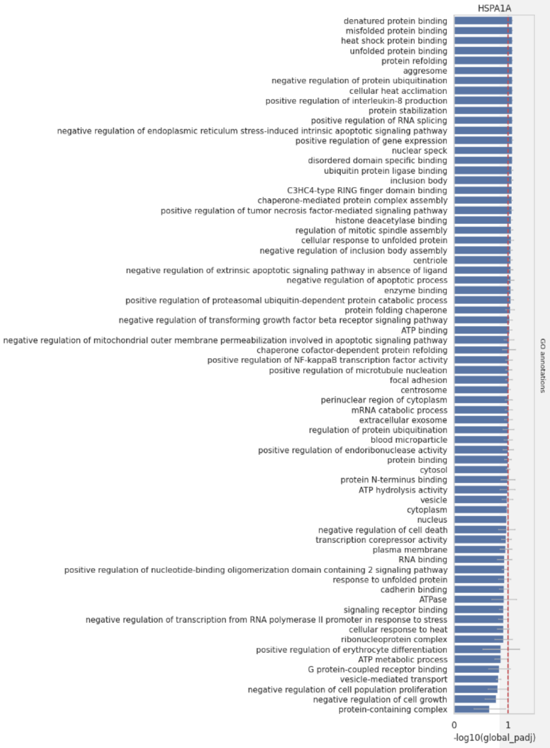
HSPA1A
denatured protein binding
misfolded protein binding
heat shock protein binding
unfolded protein binding
protein refolding
aggresome
negative regulation of protein ubiquitination
cellular heat acclimation
positive regulation of interleukin-8 production
protein stabilization
positive regulation of RNA splicing
negative regulation of endoplasmic reticulum stress-induced intrinsic apoptotic signaling pathway
positive regulation of gene expression
nuclear speck
disordered domain specific binding
ubiquitin protein ligase binding
inclusion body
C3HC4-type RING finger domain binding
chaperone-mediated protein complex assembly
positive regulation of tumor necrosis factor-mediated signaling pathway
histone deacetylase binding
regulation of mitotic spindle assembly
cellular response to unfolded protein
negative regulation of inclusion body assembly
centriole
negative regulation of extrinsic apoptotic signaling pathway in absence of ligand
negative regulation of apoptotic process
enzyme binding
positive regulation of proteasomal ubiquitin-dependent protein catabolic process
protein folding chaperone
negative regulation of transforming growth factor beta receptor signaling pathway
ATP binding
negative regulation of mitochondrial outer membrane permeabilization involved in apoptotic signaling pathway
chaperone cofactor-dependent protein refolding
positive regulation of NF-kappaB transcription factor activity
positive regulation of microtubule nucleation
focal adhesion
centrosome
perinuclear region of cytoplasm
mRNA catabolic process
extracellular exosome
regulation of protein ubiquitination
blood microparticle
positive regulation of endoribonuclease activity
protein binding
cytosol
protein N-terminus binding
ATP hydrolysis activity
vesicle
cytoplasm
nucleus
negative regulation of cell death
transcription corepressor activity
plasma membrane
RNA binding
positive regulation of nucleotide-binding oligomerization domain containing 2 signaling pathway
response to unfolded protein
cadherin binding
ATPase
signaling receptor binding
negative regulation of transcription from RNA polymerase II promoter in response to stress
cellular response to heat
ribonucleoprotein complex
positive regulation of erythrocyte differentiation
ATP metabolic process
G protein-coupled receptor binding
vesicle-mediated transport
negative regulation of cell population proliferation
negative regulation of cell growth
protein-containing complex
0
1
-log10(global_padj)
GO annotations

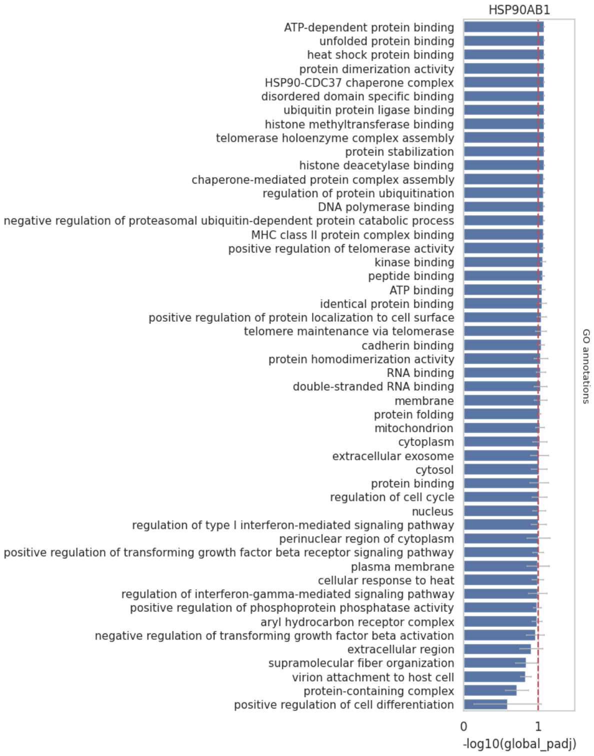
HSP90AB1
ATP-dependent protein binding
unfolded protein binding
heat shock protein binding
protein dimerization activity
HSP90-CDC37 chaperone complex
disordered domain specific binding
ubiquitin protein ligase binding
histone methyltransferase binding
telomerase holoenzyme complex assembly
protein stabilization
histone deacetylase binding
chaperone-mediated protein complex assembly
regulation of protein ubiquitination
DNA polymerase binding
negative regulation of proteasomal ubiquitin-dependent protein catabolic process
MHC class II protein complex binding
positive regulation of telomerase activity
kinase binding
peptide binding
ATP binding
identical protein binding
positive regulation of protein localization to cell surface
telomere maintenance via telomerase
cadherin binding
protein homodimerization activity
RNA binding
double-stranded RNA binding
membrane
protein folding
mitochondrion
cytoplasm
extracellular exosome
cytosol
protein binding
regulation of cell cycle
nucleus
regulation of type I interferon-mediated signaling pathway
perinuclear region of cytoplasm
positive regulation of transforming growth factor beta receptor signaling pathway
plasma membrane
cellular response to heat
regulation of interferon-gamma-mediated signaling pathway
positive regulation of phosphoprotein phosphatase activity
aryl hydrocarbon receptor complex
negative regulation of transforming growth factor beta activation
extracellular region
supramolecular fiber organization
virion attachment to host cell
protein-containing complex
positive regulation of cell differentiation
GO annotations
0
1
-log10(global_padj)

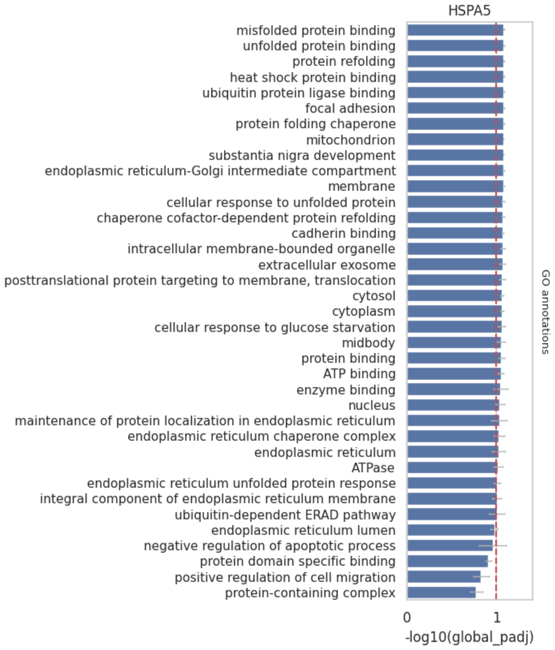
HSPA5
misfolded protein binding
unfolded protein binding
protein refolding
heat shock protein binding
ubiquitin protein ligase binding
focal adhesion
protein folding chaperone
mitochondrion
substantia nigra development
endoplasmic reticulum-Golgi intermediate compartment
membrane
cellular response to unfolded protein
chaperone cofactor-dependent protein refolding
cadherin binding
intracellular membrane-bounded organelle
extracellular exosome
posttranslational protein targeting to membrane, translocation
cytosol
cytoplasm
cellular response to glucose starvation
midbody
protein binding
ATP binding
enzyme binding
nucleus
maintenance of protein localization in endoplasmic reticulum
endoplasmic reticulum chaperone complex
endoplasmic reticulum
ATPase
endoplasmic reticulum unfolded protein response
integral component of endoplasmic reticulum membrane
ubiquitin-dependent ERAD pathway
endoplasmic reticulum lumen
negative regulation of apoptotic process
protein domain specific binding
positive regulation of cell migration
protein-containing complex
GO annotations
0
1
-log10(global_padj)

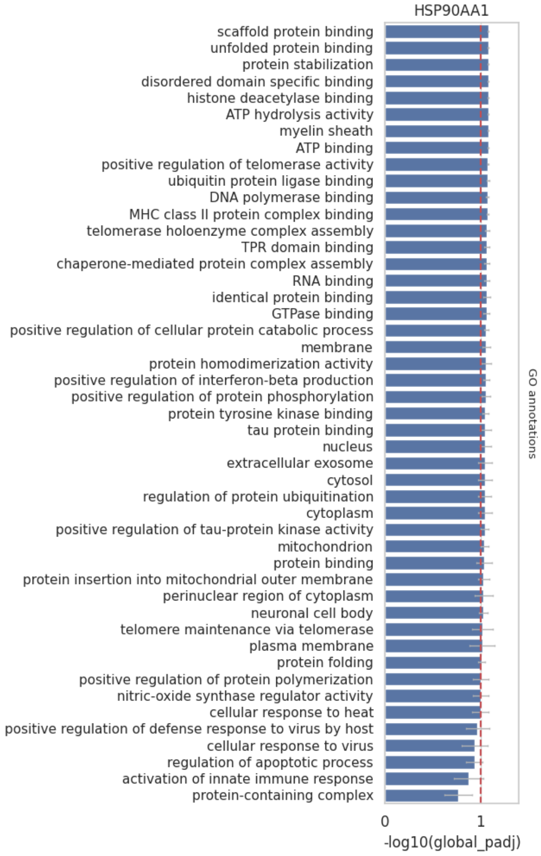
HSP90AA1
scaffold protein binding
unfolded protein binding
protein stabilization
disordered domain specific binding
histone deacetylase binding
ATP hydrolysis activity
myelin sheath
ATP binding
positive regulation of telomerase activity
ubiquitin protein ligase binding
DNA polymerase binding
MHC class II protein complex binding
telomerase holoenzyme complex assembly
TPR domain binding
chaperone-mediated protein complex assembly
RNA binding
identical protein binding
GTPase binding
positive regulation of cellular protein catabolic process
membrane
protein homodimerization activity
positive regulation of interferon-beta production
positive regulation of protein phosphorylation
protein tyrosine kinase binding
tau protein binding
nucleus
extracellular exosome
cytosol
regulation of protein ubiquitination
cytoplasm
positive regulation of tau-protein kinase activity
mitochondrion
protein binding
protein insertion into mitochondrial outer membrane
perinuclear region of cytoplasm
neuronal cell body
telomere maintenance via telomerase
plasma membrane
protein folding
positive regulation of protein polymerization
nitric-oxide synthase regulator activity
cellular response to heat
positive regulation of defense response to virus by host
cellular response to virus
regulation of apoptotic process
activation of innate immune response
protein-containing complex
0
1
-log10(global_padj)
GO annotations

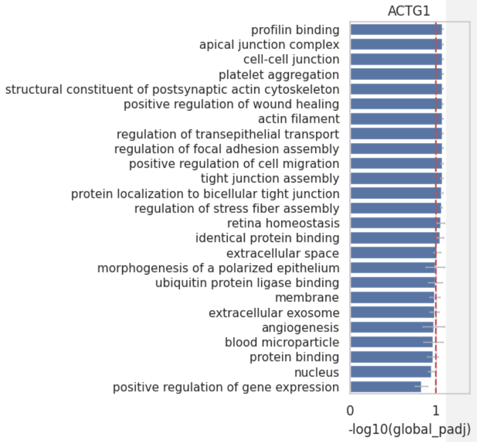
ACTG1
profilin binding
apical junction complex
cell-cell junction
platelet aggregation
structural constituent of postsynaptic actin cytoskeleton
positive regulation of wound healing
actin filament
regulation of transepithelial transport
regulation of focal adhesion assembly
positive regulation of cell migration
tight junction assembly
protein localization to bicellular tight junction
regulation of stress fiber assembly
retina homeostasis
identical protein binding
extracellular space
morphogenesis of a polarized epithelium
ubiquitin protein ligase binding
membrane
extracellular exosome
angiogenesis
blood microparticle
protein binding
nucleus
positive regulation of gene expression
0
1
-log10(global_padj)
GO annotations

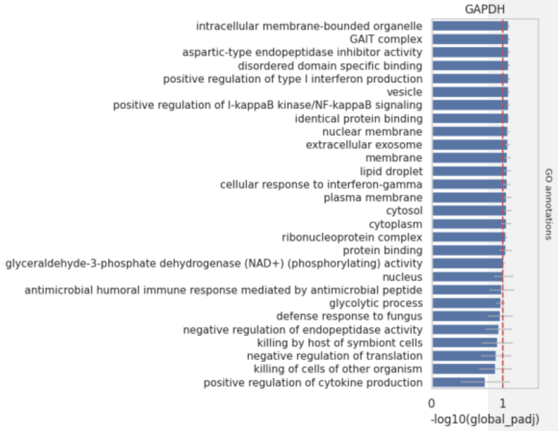
GAPDH
intracellular membrane-bounded organelle
GAIT complex
aspartic-type endopeptidase inhibitor activity
disordered domain specific binding
positive regulation of type I interferon production
vesicle
positive regulation of I-kappaB kinase/NF-kappaB signaling
identical protein binding
nuclear membrane
extracellular exosome
membrane
lipid droplet
cellular response to interferon-gamma
plasma membrane
cytosol
cytoplasm
ribonucleoprotein complex
protein binding
glyceraldehyde-3-phosphate dehydrogenase (NAD+) (phosphorylating) activity
nucleus
antimicrobial humoral immune response mediated by antimicrobial peptide
glycolytic process
defense response to fungus
negative regulation of endopeptidase activity
killing by host of symbiont cells
negative regulation of translation
killing of cells of other organism
positive regulation of cytokine production
0
1
-log10(global_padj)
GO annotations

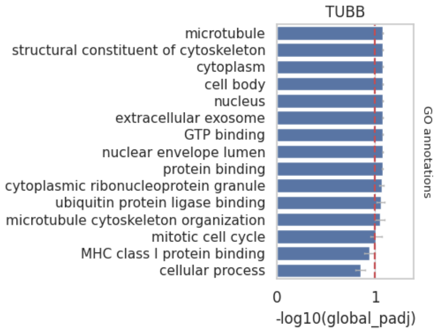
TUBB
microtubule
structural constituent of cytoskeleton
cytoplasm
cell body
nucleus
extracellular exosome
GTP binding
nuclear envelope lumen
protein binding
cytoplasmic ribonucleoprotein granule
ubiquitin protein ligase binding
microtubule cytoskeleton organization
mitotic cell cycle
MHC class I protein binding
cellular process
0
1
-log10(global_padj)
GO annotations

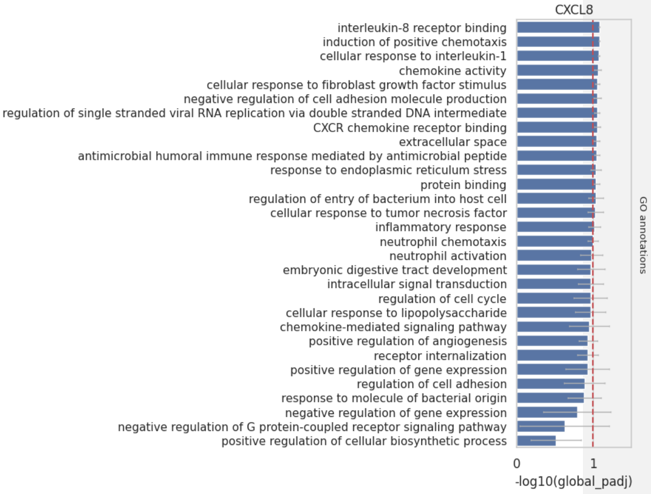
CXCL8
interleukin-8 receptor binding
induction of positive chemotaxis
cellular response to interleukin-1
chemokine activity
cellular response to fibroblast growth factor stimulus
negative regulation of cell adhesion molecule production
regulation of single stranded viral RNA replication via double stranded DNA intermediate
CXCR chemokine receptor binding
extracellular space
antimicrobial humoral immune response mediated by antimicrobial peptide
response to endoplasmic reticulum stress
protein binding
regulation of entry of bacterium into host cell
cellular response to tumor necrosis factor
inflammatory response
neutrophil chemotaxis
neutrophil activation
embryonic digestive tract development
intracellular signal transduction
regulation of cell cycle
cellular response to lipopolysaccharide
chemokine-mediated signaling pathway
positive regulation of angiogenesis
receptor internalization
positive regulation of gene expression
regulation of cell adhesion
response to molecule of bacterial origin
negative regulation of gene expression
negative regulation of G protein-coupled receptor signaling pathway
positive regulation of cellular biosynthetic process
0
1
-log10(global_padj)
GO annotations

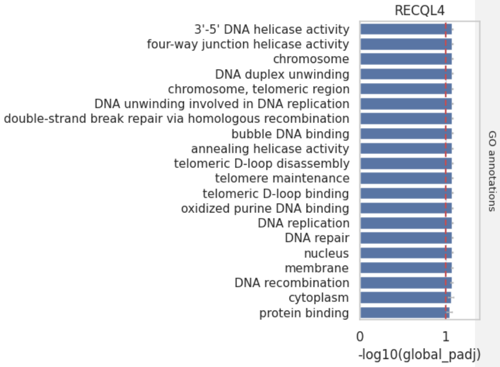
RECQL4
3'-5' DNA helicase activity
four-way junction helicase activity
chromosome
DNA duplex unwinding
chromosome, telomeric region
DNA unwinding involved in DNA replication
double-strand break repair via homologous recombination
bubble DNA binding
annealing helicase activity
telomeric D-loop disassembly
telomere maintenance
telomeric D-loop binding
oxidized purine DNA binding
DNA replication
DNA repair
nucleus
membrane
DNA recombination
cytoplasm
protein binding
0
1
-log10(global_padj)
GO annotations

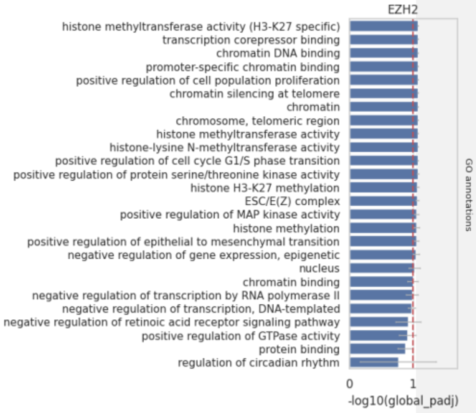
EZH2
histone methyltransferase activity (H3-K27 specific)
transcription corepressor binding
chromatin DNA binding
promoter-specific chromatin binding
positive regulation of cell population proliferation
chromatin silencing at telomere
chromatin
chromosome, telomeric region
histone methyltransferase activity
histone-lysine N-methyltransferase activity
positive regulation of cell cycle G1/S phase transition
positive regulation of protein serine/threonine kinase activity
histone H3-K27 methylation
ESC/E(Z) complex
positive regulation of MAP kinase activity
histone methylation
positive regulation of epithelial to mesenchymal transition
negative regulation of gene expression, epigenetic
nucleus
chromatin binding
negative regulation of transcription by RNA polymerase II
negative regulation of transcription, DNA-templated
negative regulation of retinoic acid receptor signaling pathway
positive regulation of GTPase activity
protein binding
regulation of circadian rhythm
0
1
-log10(global_padj)
GO annotations

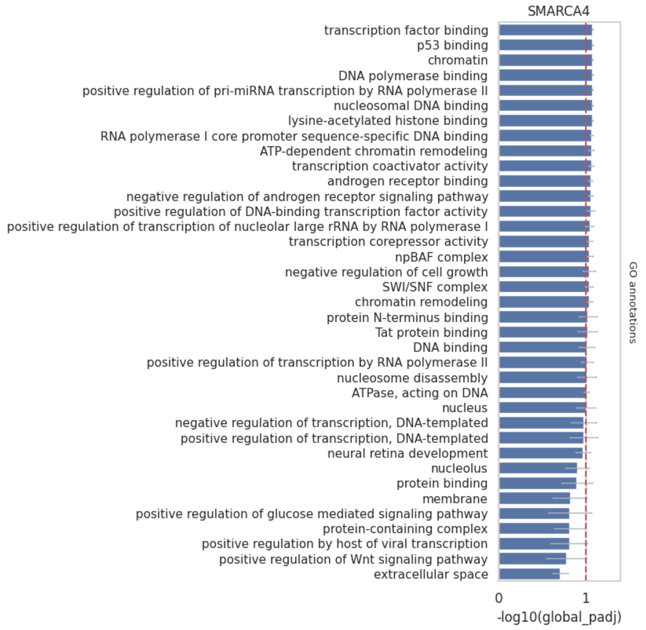
SMARCA4
transcription factor binding
p53 binding
chromatin
DNA polymerase binding
positive regulation of pri-miRNA transcription by RNA polymerase II
nucleosomal DNA binding
lysine-acetylated histone binding
RNA polymerase I core promoter sequence-specific DNA binding
ATP-dependent chromatin remodeling
transcription coactivator activity
androgen receptor binding
negative regulation of androgen receptor signaling pathway
positive regulation of DNA-binding transcription factor activity
positive regulation of transcription of nucleolar large rRNA by RNA polymerase I
transcription corepressor activity
npBAF complex
negative regulation of cell growth
SWI/SNF complex
chromatin remodeling
protein N-terminus binding
Tat protein binding
DNA binding
positive regulation of transcription by RNA polymerase II
nucleosome disassembly
ATPase, acting on DNA
nucleus
negative regulation of transcription, DNA-templated
positive regulation of transcription, DNA-templated
neural retina development
nucleolus
protein binding
membrane
positive regulation of glucose mediated signaling pathway
protein-containing complex
positive regulation by host of viral transcription
positive regulation of Wnt signaling pathway
extracellular space
0
1
-log10(global_padj)
GO annotations

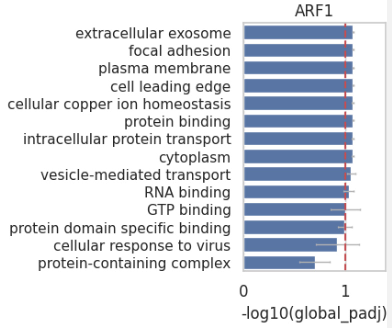
ARF1
extracellular exosome
focal adhesion
plasma membrane
cell leading edge
cellular copper ion homeostasis
protein binding
intracellular protein transport
cytoplasm
vesicle-mediated transport
RNA binding
GTP binding
protein domain specific binding
cellular response to virus
protein-containing complex
0
1
-log10(global_padj)
GO annotations

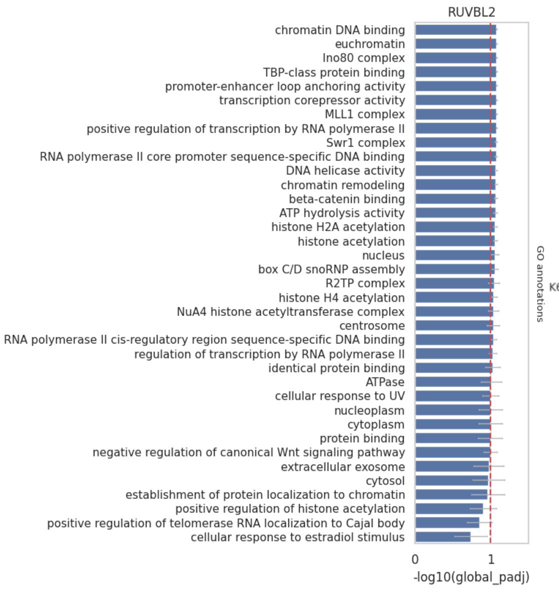
RUVBL2
chromatin DNA binding
euchromatin
Ino80 complex
TBP-class protein binding
promoter-enhancer loop anchoring activity
transcription corepressor activity
MLL1 complex
positive regulation of transcription by RNA polymerase II
Swr1 complex
RNA polymerase II core promoter sequence-specific DNA binding
DNA helicase activity
chromatin remodeling
beta-catenin binding
ATP hydrolysis activity
histone H2A acetylation
histone acetylation
nucleus
box C/D snoRNP assembly
R2TP complex
histone H4 acetylation
NuA4 histone acetyltransferase complex
centrosome
RNA polymerase II cis-regulatory region sequence-specific DNA binding
regulation of transcription by RNA polymerase II
identical protein binding
ATPase
cellular response to UV
nucleoplasm
cytoplasm
protein binding
negative regulation of canonical Wnt signaling pathway
extracellular exosome
cytosol
establishment of protein localization to chromatin
positive regulation of histone acetylation
positive regulation of telomerase RNA localization to Cajal body
cellular response to estradiol stimulus
0
1
-log10(global_padj)
GO annotations

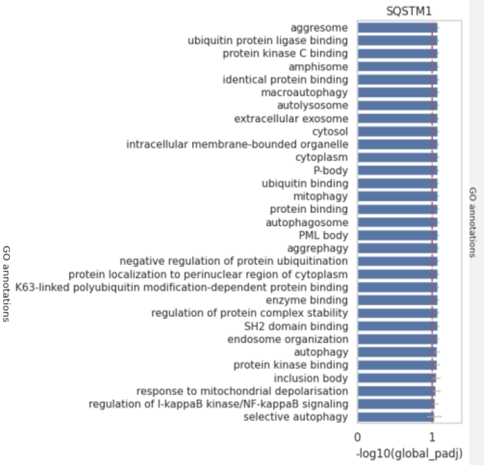
SQSTM1
aggresome
ubiquitin protein ligase binding
protein kinase C binding
amphisome
identical protein binding
macroautophagy
autolysosome
extracellular exosome
cytosol
intracellular membrane-bounded organelle
cytoplasm
P-body
ubiquitin binding
mitophagy
protein binding
autophagosome
PML body
aggrephagy
negative regulation of protein ubiquitination
protein localization to perinuclear region of cytoplasm
K63-linked polyubiquitin modification-dependent protein binding
enzyme binding
regulation of protein complex stability
SH2 domain binding
endosome organization
autophagy
protein kinase binding
inclusion body
response to mitochondrial depolarisation
regulation of I-kappaB kinase/NF-kappaB signaling
selective autophagy
0
1
-log10(global_padj)
GO annotations

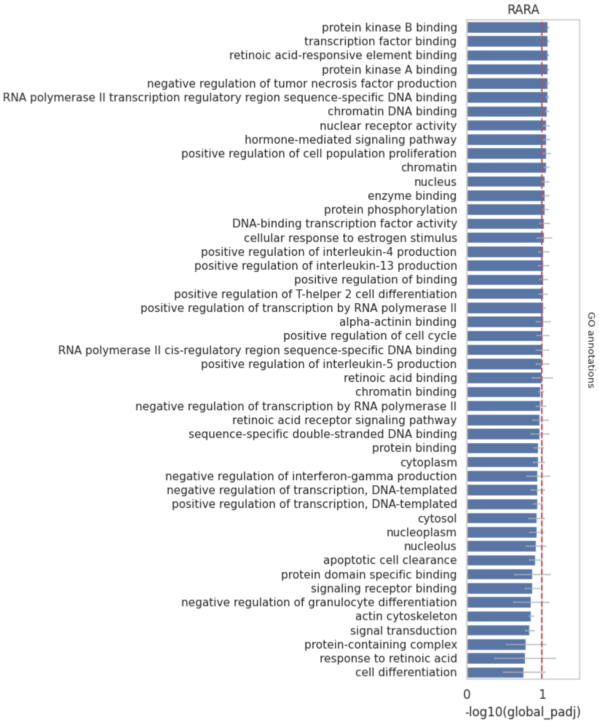
RARA
protein kinase B binding
transcription factor binding
retinoic acid-responsive element binding
protein kinase A binding
negative regulation of tumor necrosis factor production
RNA polymerase II transcription regulatory region sequence-specific DNA binding
chromatin DNA binding
nuclear receptor activity
hormone-mediated signaling pathway
positive regulation of cell population proliferation
chromatin
nucleus
enzyme binding
protein phosphorylation
DNA-binding transcription factor activity
cellular response to estrogen stimulus
positive regulation of interleukin-4 production
positive regulation of interleukin-13 production
positive regulation of binding
positive regulation of T-helper 2 cell differentiation
positive regulation of transcription by RNA polymerase II
alpha-actinin binding
positive regulation of cell cycle
RNA polymerase II cis-regulatory region sequence-specific DNA binding
positive regulation of interleukin-5 production
retinoic acid binding
chromatin binding
negative regulation of transcription by RNA polymerase II
retinoic acid receptor signaling pathway
sequence-specific double-stranded DNA binding
protein binding
cytoplasm
negative regulation of interferon-gamma production
negative regulation of transcription, DNA-templated
positive regulation of transcription, DNA-templated
cytosol
nucleoplasm
nucleolus
apoptotic cell clearance
protein domain specific binding
signaling receptor binding
negative regulation of granulocyte differentiation
actin cytoskeleton
signal transduction
protein-containing complex
response to retinoic acid
cell differentiation
0
1
-log10(global_padj)
GO annotations

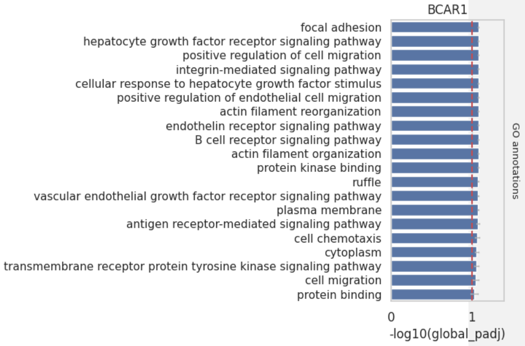
BCAR1
focal adhesion
hepatocyte growth factor receptor signaling pathway
positive regulation of cell migration
integrin-mediated signaling pathway
cellular response to hepatocyte growth factor stimulus
positive regulation of endothelial cell migration
actin filament reorganization
endothelin receptor signaling pathway
B cell receptor signaling pathway
actin filament organization
protein kinase binding
ruffle
vascular endothelial growth factor receptor signaling pathway
plasma membrane
antigen receptor-mediated signaling pathway
cell chemotaxis
cytoplasm
transmembrane receptor protein tyrosine kinase signaling pathway
cell migration
protein binding
0
1
-log10(global_padj)
GO annotations

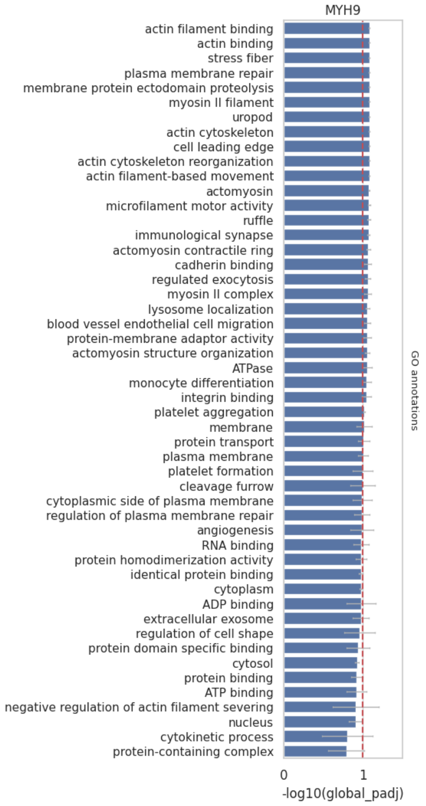
MYH9
actin filament binding
actin binding
stress fiber
plasma membrane repair
membrane protein ectodomain proteolysis
myosin II filament
uropod
actin cytoskeleton
cell leading edge
actin cytoskeleton reorganization
actin filament-based movement
actomyosin
microfilament motor activity
ruffle
immunological synapse
actomyosin contractile ring
cadherin binding
regulated exocytosis
myosin II complex
lysosome localization
blood vessel endothelial cell migration
protein-membrane adaptor activity
actomyosin structure organization
ATPase
monocyte differentiation
integrin binding
platelet aggregation
membrane
protein transport
plasma membrane
platelet formation
cleavage furrow
cytoplasmic side of plasma membrane
regulation of plasma membrane repair
angiogenesis
RNA binding
protein homodimerization activity
identical protein binding
cytoplasm
ADP binding
extracellular exosome
regulation of cell shape
protein domain specific binding
cytosol
protein binding
ATP binding
negative regulation of actin filament severing
nucleus
cytokinetic process
protein-containing complex
0
1
-log10(global_padj)
GO annotations

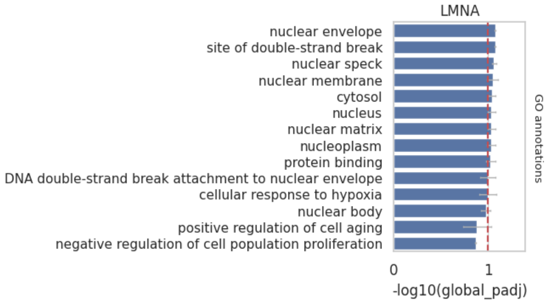
LMNA
nuclear envelope
site of double-strand break
nuclear speck
nuclear membrane
cytosol
nucleus
nuclear matrix
nucleoplasm
protein binding
DNA double-strand break attachment to nuclear envelope
cellular response to hypoxia
nuclear body
positive regulation of cell aging
negative regulation of cell population proliferation
0
1
-log10(global_padj)
GO annotations

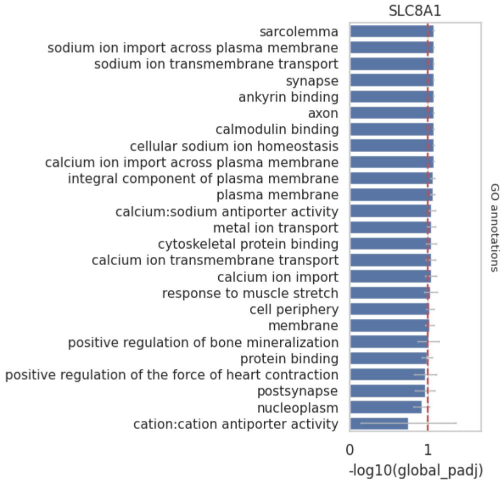
SLC8A1
sarcolemma
sodium ion import across plasma membrane
sodium ion transmembrane transport
synapse
ankyrin binding
axon
calmodulin binding
cellular sodium ion homeostasis
calcium ion import across plasma membrane
integral component of plasma membrane
plasma membrane
calcium:sodium antiporter activity
metal ion transport
cytoskeletal protein binding
calcium ion transmembrane transport
calcium ion import
response to muscle stretch
cell periphery
membrane
positive regulation of bone mineralization
protein binding
positive regulation of the force of heart contraction
postsynapse
nucleoplasm
cation:cation antiporter activity
0
1
-log10(global_padj)
GO annotations

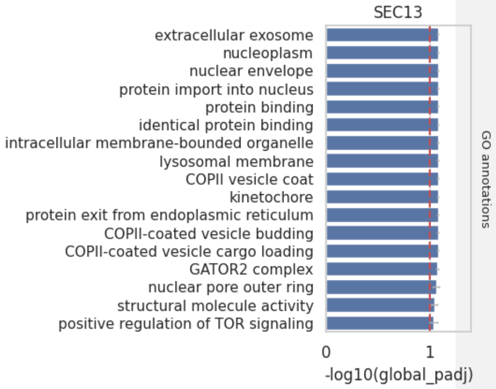
SEC13
extracellular exosome
nucleoplasm
nuclear envelope
protein import into nucleus
protein binding
identical protein binding
intracellular membrane-bounded organelle
lysosomal membrane
COPII vesicle coat
kinetochore
protein exit from endoplasmic reticulum
COPII-coated vesicle budding
COPII-coated vesicle cargo loading
GATOR2 complex
nuclear pore outer ring
structural molecule activity
positive regulation of TOR signaling
0
1
-log10(global_padj)
GO annotations

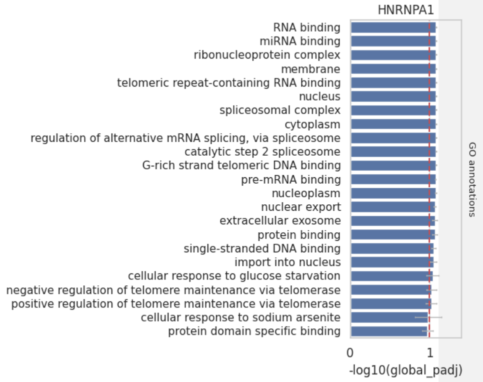
HNRNPA1
RNA binding
miRNA binding
ribonucleoprotein complex
membrane
telomeric repeat-containing RNA binding
nucleus
spliceosomal complex
cytoplasm
regulation of alternative mRNA splicing, via spliceosome
catalytic step 2 spliceosome
G-rich strand telomeric DNA binding
pre-mRNA binding
nucleoplasm
nuclear export
extracellular exosome
protein binding
single-stranded DNA binding
import into nucleus
cellular response to glucose starvation
negative regulation of telomere maintenance via telomerase
positive regulation of telomere maintenance via telomerase
cellular response to sodium arsenite
protein domain specific binding
0
1
-log10(global_padj)
GO annotations

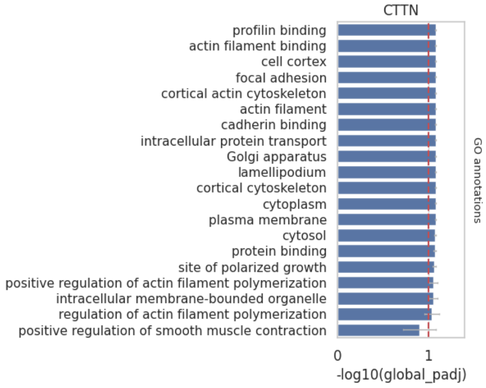
CTTN
profilin binding
actin filament binding
cell cortex
focal adhesion
cortical actin cytoskeleton
actin filament
cadherin binding
intracellular protein transport
Golgi apparatus
lamellipodium
cortical cytoskeleton
cytoplasm
plasma membrane
cytosol
protein binding
site of polarized growth
positive regulation of actin filament polymerization
intracellular membrane-bounded organelle
regulation of actin filament polymerization
positive regulation of smooth muscle contraction
0
1
-log10(global_padj)
GO annotations

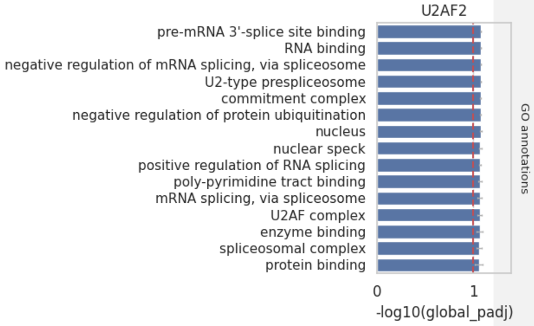
U2AF2
pre-mRNA 3'-splice site binding
RNA binding
negative regulation of mRNA splicing, via spliceosome
U2-type prespliceosome
commitment complex
negative regulation of protein ubiquitination
nucleus
nuclear speck
positive regulation of RNA splicing
poly-pyrimidine tract binding
mRNA splicing, via spliceosome
U2AF complex
enzyme binding
spliceosomal complex
protein binding
0
1
-log10(global_padj)
GO annotations

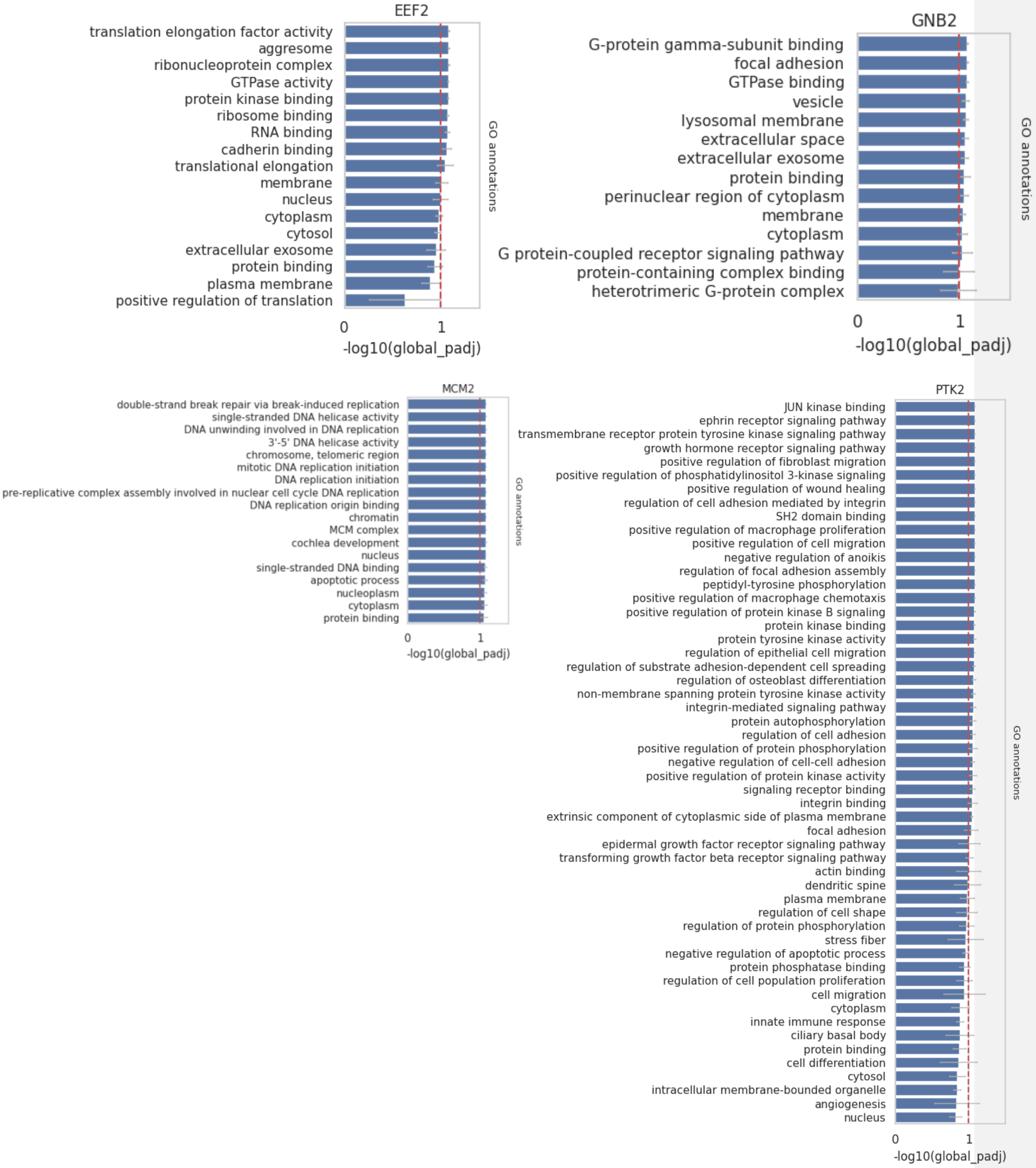
EEF2
translation elongation factor activity
aggresome
ribonucleoprotein complex
GTPase activity
protein kinase binding
ribosome binding
RNA binding
cadherin binding
translational elongation
membrane
nucleus
cytoplasm
cytosol
extracellular exosome
protein binding
plasma membrane
positive regulation of translation
GO annotations
0
1
-log10(global_padj)
GNB2
G-protein gamma-subunit binding
focal adhesion
GTPase binding
vesicle
lysosomal membrane
extracellular space
extracellular exosome
protein binding
perinuclear region of cytoplasm
membrane
cytoplasm
G protein-coupled receptor signaling pathway
protein-containing complex binding
heterotrimeric G-protein complex
GO annotations
0
1
-log10(global_padj)
MCM2
double-strand break repair via break-induced replication
single-stranded DNA helicase activity
DNA unwinding involved in DNA replication
3'-5' DNA helicase activity
chromosome, telomeric region
mitotic DNA replication initiation
DNA replication initiation
pre-replicative complex assembly involved in nuclear cell cycle DNA replication
DNA replication origin binding
chromatin
MCM complex
cochlea development
nucleus
single-stranded DNA binding
apoptotic process
nucleoplasm
cytoplasm
protein binding
GO annotations
0
1
-log10(global_padj)
PTK2
JUN kinase binding
ephrin receptor signaling pathway
transmembrane receptor protein tyrosine kinase signaling pathway
growth hormone receptor signaling pathway
positive regulation of fibroblast migration
positive regulation of phosphatidylinositol 3-kinase signaling
positive regulation of wound healing
regulation of cell adhesion mediated by integrin
SH2 domain binding
positive regulation of macrophage proliferation
positive regulation of cell migration
negative regulation of anoikis
regulation of focal adhesion assembly
peptidyl-tyrosine phosphorylation
positive regulation of macrophage chemotaxis
positive regulation of protein kinase B signaling
protein kinase binding
protein tyrosine kinase activity
regulation of epithelial cell migration
regulation of substrate adhesion-dependent cell spreading
regulation of osteoblast differentiation
non-membrane spanning protein tyrosine kinase activity
integrin-mediated signaling pathway
protein autophosphorylation
regulation of cell adhesion
positive regulation of protein phosphorylation
negative regulation of cell-cell adhesion
positive regulation of protein kinase activity
signaling receptor binding
integrin binding
extrinsic component of cytoplasmic side of plasma membrane
focal adhesion
epidermal growth factor receptor signaling pathway
transforming growth factor beta receptor signaling pathway
actin binding
dendritic spine
plasma membrane
regulation of cell shape
regulation of protein phosphorylation
stress fiber
negative regulation of apoptotic process
protein phosphatase binding
regulation of cell population proliferation
cell migration
cytoplasm
innate immune response
ciliary basal body
protein binding
cell differentiation
cytosol
intracellular membrane-bounded organelle
angiogenesis
nucleus
GO annotations
0
1
-log10(global_padj)

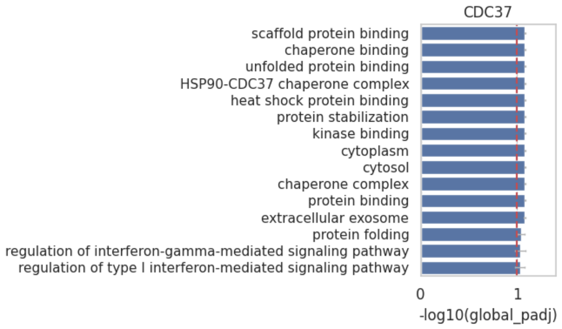

Non viral Moonlightning genes

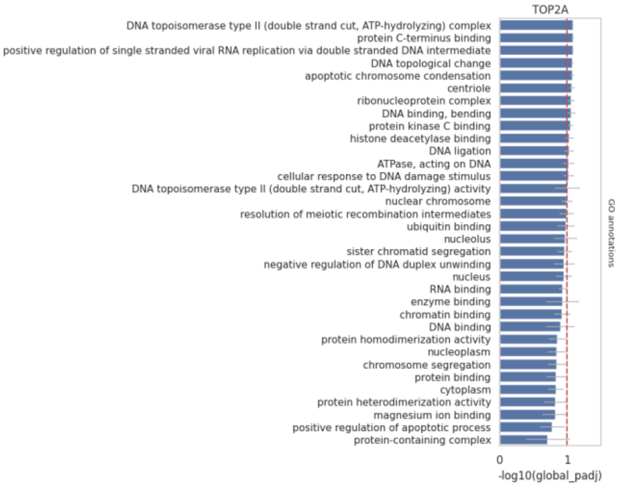

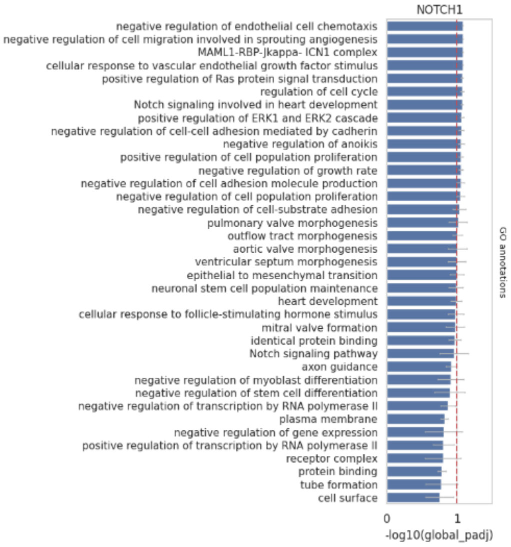
NOTCH1
negative regulation of endothelial cell chemotaxis
negative regulation of cell migration involved in sprouting angiogenesis
MAML1-RBP-Jkappa- ICN1 complex
cellular response to vascular endothelial growth factor stimulus
positive regulation of Ras protein signal transduction
regulation of cell cycle
Notch signaling involved in heart development
positive regulation of ERK1 and ERK2 cascade
negative regulation of cell-cell adhesion mediated by cadherin
negative regulation of anoikis
positive regulation of cell population proliferation
negative regulation of growth rate
negative regulation of cell adhesion molecule production
negative regulation of cell population proliferation
negative regulation of cell-substrate adhesion
pulmonary valve morphogenesis
outflow tract morphogenesis
aortic valve morphogenesis
ventricular septum morphogenesis
epithelial to mesenchymal transition
neuronal stem cell population maintenance
heart development
cellular response to follicle-stimulating hormone stimulus
mitral valve formation
identical protein binding
Notch signaling pathway
axon guidance
negative regulation of myoblast differentiation
negative regulation of stem cell differentiation
negative regulation of transcription by RNA polymerase II
plasma membrane
negative regulation of gene expression
positive regulation of transcription by RNA polymerase II
receptor complex
protein binding
tube formation
cell surface
0
1
-log10(global_padj)
GO annotations

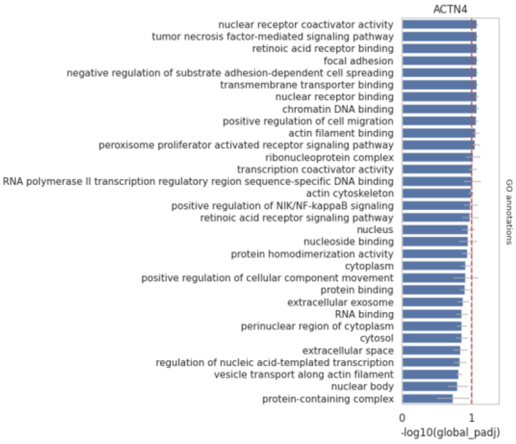
ACTN4
nuclear receptor coactivator activity
tumor necrosis factor-mediated signaling pathway
retinoic acid receptor binding
focal adhesion
negative regulation of substrate adhesion-dependent cell spreading
transmembrane transporter binding
nuclear receptor binding
chromatin DNA binding
positive regulation of cell migration
actin filament binding
peroxisome proliferator activated receptor signaling pathway
ribonucleoprotein complex
transcription coactivator activity
RNA polymerase II transcription regulatory region sequence-specific DNA binding
actin cytoskeleton
positive regulation of NIK/NF-kappaB signaling
retinoic acid receptor signaling pathway
nucleus
nucleoside binding
protein homodimerization activity
cytoplasm
positive regulation of cellular component movement
protein binding
extracellular exosome
RNA binding
perinuclear region of cytoplasm
cytosol
extracellular space
regulation of nucleic acid-templated transcription
vesicle transport along actin filament
nuclear body
protein-containing complex
0
1
-log10(global_padj)
GO annotations

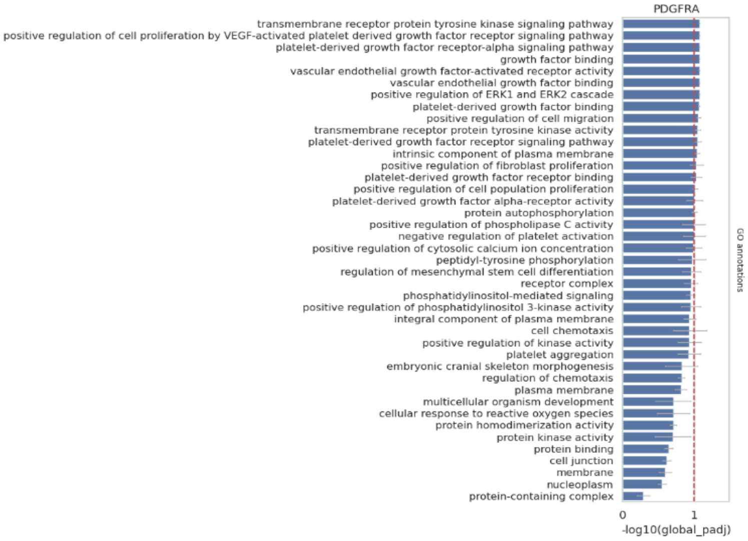
PDGFRA
transmembrane receptor protein tyrosine kinase signaling pathway
positive regulation of cell proliferation by VEGF-activated platelet derived growth factor receptor signaling pathway
platelet-derived growth factor receptor-alpha signaling pathway
growth factor binding
vascular endothelial growth factor-activated receptor activity
vascular endothelial growth factor binding
positive regulation of ERK1 and ERK2 cascade
platelet-derived growth factor binding
positive regulation of cell migration
transmembrane receptor protein tyrosine kinase activity
platelet-derived growth factor receptor signaling pathway
intrinsic component of plasma membrane
positive regulation of fibroblast proliferation
platelet-derived growth factor receptor binding
positive regulation of cell population proliferation
platelet-derived growth factor alpha-receptor activity
protein autophosphorylation
positive regulation of phospholipase C activity
negative regulation of platelet activation
positive regulation of cytosolic calcium ion concentration
peptidyl-tyrosine phosphorylation
regulation of mesenchymal stem cell differentiation
receptor complex
phosphatidylinositol-mediated signaling
positive regulation of phosphatidylinositol 3-kinase activity
integral component of plasma membrane
cell chemotaxis
positive regulation of kinase activity
platelet aggregation
embryonic cranial skeleton morphogenesis
regulation of chemotaxis
plasma membrane
multicellular organism development
cellular response to reactive oxygen species
protein homodimerization activity
protein kinase activity
protein binding
cell junction
membrane
nucleoplasm
protein-containing complex
0
1
-log10(global_padj)
GO annotations

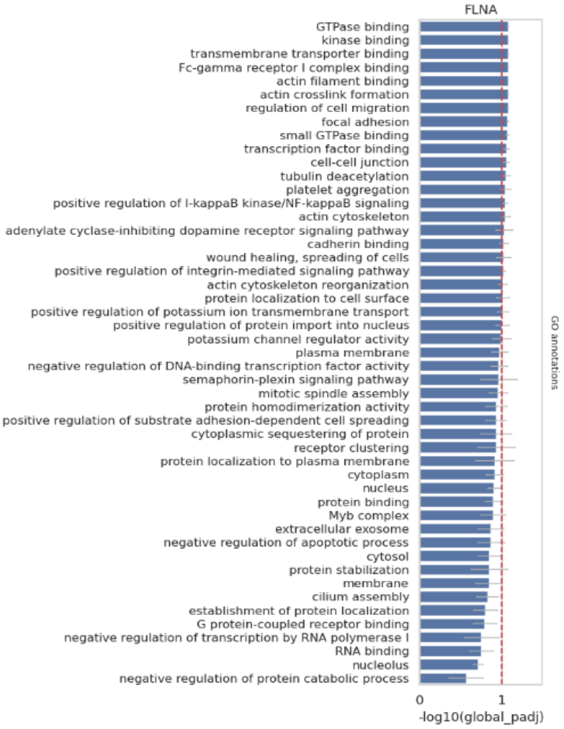
FLNA
GTPase binding
kinase binding
transmembrane transporter binding
Fc-gamma receptor I complex binding
actin filament binding
actin crosslink formation
regulation of cell migration
focal adhesion
small GTPase binding
transcription factor binding
cell-cell junction
tubulin deacetylation
platelet aggregation
positive regulation of I-kappaB kinase/NF-kappaB signaling
actin cytoskeleton
adenylate cyclase-inhibiting dopamine receptor signaling pathway
cadherin binding
wound healing, spreading of cells
positive regulation of integrin-mediated signaling pathway
actin cytoskeleton reorganization
protein localization to cell surface
positive regulation of potassium ion transmembrane transport
positive regulation of protein import into nucleus
potassium channel regulator activity
plasma membrane
negative regulation of DNA-binding transcription factor activity
semaphorin-plexin signaling pathway
mitotic spindle assembly
protein homodimerization activity
positive regulation of substrate adhesion-dependent cell spreading
cytoplasmic sequestering of protein
receptor clustering
protein localization to plasma membrane
cytoplasm
nucleus
protein binding
Myb complex
extracellular exosome
negative regulation of apoptotic process
cytosol
protein stabilization
membrane
cilium assembly
establishment of protein localization
G protein-coupled receptor binding
negative regulation of transcription by RNA polymerase I
RNA binding
nucleolus
negative regulation of protein catabolic process
0
1
-log10(global_padj)
GO annotations

Supplementary Figure 5. Immunome analysis in tumor tissues versus non-tumoral tissues and in TCGA cohort

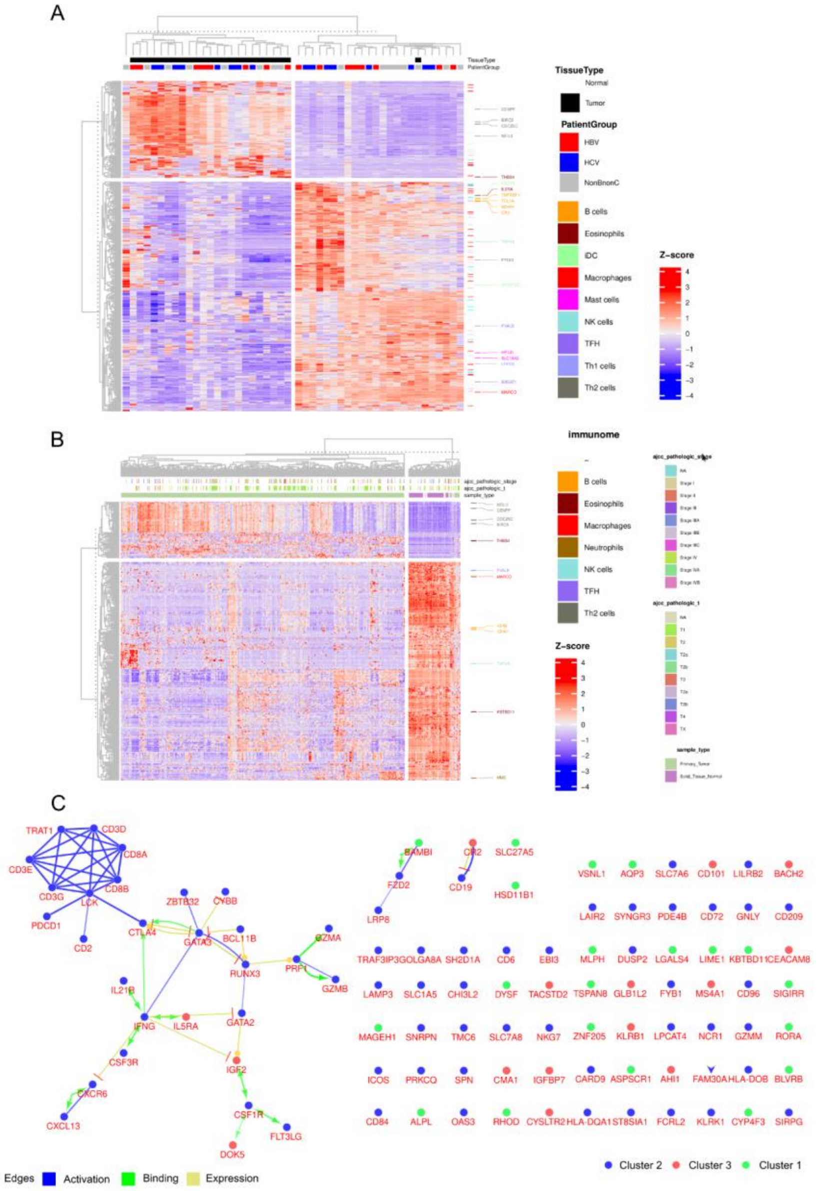

The heatmap with genes differentially expressed in tumors compared to normal tissue samples from Bucuresti cohort (A) and TCGA cohort (B). Data was normalized and hierarchically clustered (Euclidean distance). Z-score spans between -4 (blue) and 4 (red). Immunome markers are highlighted. (A) Fold change >2.5 and adjusted pvalue <0.005. (B) Fold change >3, adjusted pvalue <0.001. (C) CluePedia scores for markers from Cluster 1 (green), Cluster 2 (blue) and Cluster 3 (red). Interactions are based on data from STRING (v11) database. Edges show activation (green), binding (blue), expression (yellow) scores.
